# Asymmetry of safeguarding regional air and water nitrogen boundaries in China

**DOI:** 10.1093/nsr/nwag113

**Published:** 2026-02-16

**Authors:** Yiyang Zou, Xiuming Zhang, Xin Xu, Jiami Wu, Luxi Cheng, Xinpeng Xu, Ouping Deng, Yuanyuan Chen, Chen Wang, Peiying He, Sitong Wang, Mengru Wang, Wilfried Winiwarter, Baojing Gu

**Affiliations:** State Key Laboratory of Soil Pollution Control and Safety, Zhejiang University, Hangzhou 310058, China; College of Environmental & Resource Sciences, Zhejiang University, Hangzhou 310058, China; Pollution Management Group, International Institute for Applied Systems Analysis, Laxenburg 2361, Austria; State Key Laboratory of Soil Pollution Control and Safety, Zhejiang University, Hangzhou 310058, China; College of Environmental & Resource Sciences, Zhejiang University, Hangzhou 310058, China; College of Environmental & Resource Sciences, Zhejiang University, Hangzhou 310058, China; State Key Laboratory of Soil Pollution Control and Safety, Zhejiang University, Hangzhou 310058, China; College of Environmental & Resource Sciences, Zhejiang University, Hangzhou 310058, China; School of Earth Sciences, Zhejiang University, Hangzhou 310058, China; College of Resources, Sichuan Agricultural University, Chengdu 611130, China; State Key Laboratory of Soil Pollution Control and Safety, Zhejiang University, Hangzhou 310058, China; College of Environmental & Resource Sciences, Zhejiang University, Hangzhou 310058, China; State Key Laboratory of Soil Pollution Control and Safety, Zhejiang University, Hangzhou 310058, China; College of Environmental & Resource Sciences, Zhejiang University, Hangzhou 310058, China; State Key Laboratory of Soil Pollution Control and Safety, Zhejiang University, Hangzhou 310058, China; College of Environmental & Resource Sciences, Zhejiang University, Hangzhou 310058, China; State Key Laboratory of Soil Pollution Control and Safety, Zhejiang University, Hangzhou 310058, China; College of Environmental & Resource Sciences, Zhejiang University, Hangzhou 310058, China; Earth Systems and Global Change Group, Wageningen University & Research, Wageningen PB6708, The Netherlands; Pollution Management Group, International Institute for Applied Systems Analysis, Laxenburg 2361, Austria; Institute of Environmental Engineering, University of Zielona Góra, Zielona Góra 65-417, Poland; State Key Laboratory of Soil Pollution Control and Safety, Zhejiang University, Hangzhou 310058, China; College of Environmental & Resource Sciences, Zhejiang University, Hangzhou 310058, China; Policy Simulation Laboratory, Zhejiang University, Hangzhou 310058, China

**Keywords:** regional boundaries, nitrogen management, cost–benefit analysis, mitigation potential, water pollution

## Abstract

Human activities have significantly disrupted the global nitrogen cycle, positioning it as one of the most severely surpassed planetary boundaries. As the country with the largest nitrogen flux, China faces numerous environmental challenges due to excessive losses of reactive nitrogen (N_r_) to both air and water from various sources. By quantifying the regional nitrogen boundaries for air and water at the county level, we found that the aggregated regional safe boundaries in China for the atmospheric release of N_r_, nitrogen runoff to surface water and leaching to groundwater are 14.6, 5.2 and 4.8 million tonnes per year, respectively. In 2020, the cumulative N_r_ losses exceeded these boundaries by 54%, 262% and 258%, respectively. Implementing cross-system technical mitigation measures could potentially halve the total N_r_ losses to both air and water, yielding benefits that are ∼2.5 times greater than the net implementation costs. Despite most counties being capable of meeting the emission boundary for the atmospheric release of N_r_ after abatement, the boundaries for surface water and groundwater remain exceeded in over half of the counties. This highlights a significant asymmetry in nitrogen-pollution control between air and water, further necessitating socioeconomic transformations to effectively address the persistent issue of water pollution in China.

## Teaser

This study provides a whole-system, county-level assessment of nitrogen pollution exceedance in China. It revealed a pronounced air-water asymmetry, with water pollution substantially harder to bring within safe limits than atmospheric emissions, underscoring the need to couple mitigation with broader socioeconomic transformations to address persistent water pollution.

## INTRODUCTION

Nitrogen serves as a core element in Earth’s biogeochemical cycles. Since the early twentieth century, the Haber–Bosch process has doubled the natural nitrogen cycle by intentionally converting atmospheric nitrogen gas (N_2_) into reactive nitrogen (N_r_) forms [1,2], thereby feeding more than half the world’s population through synthetic nitrogen fertilizers [2–4]. Although agricultural productivity and food security rely heavily on nitrogen availability, excessive N_r_ inputs under intensive fertilization practices have discharged substantial quantities of N_r_ into both air and water [5]. These losses trigger a cascade of environmental impacts, including biodiversity loss, air pollution, eutrophication, nitrate contamination of groundwater, stratospheric ozone depletion and climate change, all of which threaten ecosystems and human health [6–11]. Meanwhile, these issues will directly or indirectly hinder progress toward several United Nations Sustainable Development Goals (SDGs) [12], particularly SDG 2 on zero hunger, SDG 3 on good health and well-being, SDG 13 on climate action, SDG 14 on life below water and SDG 15 on life on land. These interconnected linkages highlight the pressing need for the integrated and effective management of N_r_.

The planetary boundary framework offers a paradigm by providing a ‘Safe Operating Space’ for human activities at the global scale [13]. Among the nine originally identified boundaries, the nitrogen cycle stands out as one of the most surpassed, drawing significant attention to the evaluation of safe nitrogen-loss levels to the environment [14]. A global ‘nitrogen planetary boundary’ [13,14] has been set at 62 million tonnes (Tg) N yr^−1^ for industrial and intentional biological nitrogen fixation. However, unlike other global environmental concerns such as ocean acidification and atmospheric ozone depletion, the regional impacts of excessive N_r_ losses far outweigh the global consequences [15]. Given the pronounced spatial heterogeneity in N_r_ emissions and their associated effects, it is imperative to estimate nitrogen boundaries at regional scales to inform targeted management options. Moreover, achieving food-security goals is closely linked to nitrogen use efficiency (NUE), defined as the ratio of the total nitrogen output in harvested products to the total nitrogen input [3]. A higher NUE results in reduced pollution, indicating that higher nitrogen inputs can be permitted without compromising environmental quality. Therefore, the safe nitrogen boundary should not be based solely on the total nitrogen inputs into the terrestrial ecosystem, but rather on N_r_ losses to the environment after fulfilling human nitrogen requirements through activities such as industrial and agricultural production (Table S1). While numerous studies have attempted to estimate the regional safe nitrogen boundaries [16–20], most of these efforts have focused primarily on N_r_ pollution from agriculture, with less attention given to pollution discharges from other sources, such as households and industries. This focus may have led to limitations in assessing regional boundary exceedances, particularly with the advocacy for overly ambitious reduction rates in nitrogen inputs, which could entail prohibitive implementation costs, face significant barriers and potentially jeopardize food security.

As the world’s largest producer and consumer of nitrogen fertilizer, China has substantially increased its food-production capacity over recent decades [21]. However, excessive N_r_ losses have far exceeded the environmental thresholds for both air and water, resulting in a cascade of detrimental ecological and human health consequences [17,22]. Balancing the imperative to safeguard regional nitrogen boundaries while ensuring stable food production therefore represents a critical challenge for China. Here, we first established regional safe nitrogen boundaries separately for each of the 2847 counties in China based on three environmental thresholds: (i) atmospheric N_r_ release rates aimed at preserving terrestrial biodiversity and protecting human health, (ii) nitrogen concentrations in surface water aimed at preventing eutrophication and (iii) nitrogen levels in groundwater consistent with the World Health Organization (WHO) drinking-water standard. The degree to which regional nitrogen boundaries were exceeded was quantified through an assessment of critical loads and current (2020) N_r_ losses from all sources. Second, we compiled the available technical measures across sectors from 734 peer-reviewed publications and quantified their mitigation potential in the Chinese context. We also estimated their implementation costs and associated social benefits to evaluate the overall feasibility. Finally, for each county, we developed a Cross-System nitrogen Management (CSM) scenario integrating coordinated technical mitigation measures targeting all major sources of N_r_-loss pathways, in order to determine whether the regional safe nitrogen boundaries could be respected through the adoption of technical measures alone. The insights derived from this analysis can support the development of effective, region-specific strategies for nitrogen-pollution control and provide valuable guidance for policymakers. Detailed model descriptions and associated uncertainties are elucidated in Sections S2 and S3.

## RESULTS AND DISCUSSION

### Regional nitrogen boundaries

**Figure 1.**
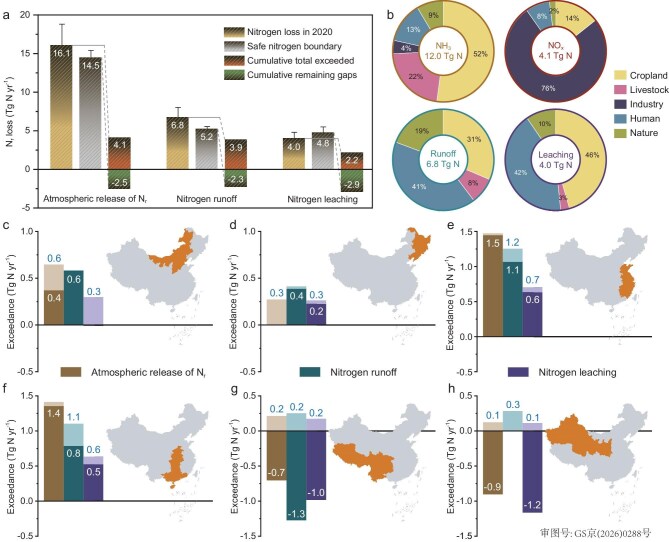
N_r_ budget, safe nitrogen boundary and the exceedance in 2020. (a) The nitrogen loss in 2020 and the safe nitrogen boundary for atmospheric release of N_r_, nitrogen runoff, and nitrogen leaching are given together with their associated uncertainties. Also included are the cumulative total exceedances across all counties in which the respective boundary is exceeded, as well as the cumulative remaining gaps between actual N_r_ loss and the boundary for the counties in which boundary has not been exceeded. It is worth noting that, for each boundary, the difference between the nitrogen loss and the safe nitrogen boundary equates to the difference between the cumulative total exceedances and the cumulative remaining gaps. (b) Sectoral contributions of four types of N_r_ losses in 2020. (c–h) The darker-shaded bars with white numbers represent the total exceedance for the corresponding boundary in each subregion. The lighter-shaded bars with blue numbers indicate the cumulative exceedance for the corresponding boundary in which the respective threshold is exceeded in each subregion. Values of <0.1 are not labeled due to their negligible magnitude. All data shown in the figures were aggregated at the county level.

The aggregated boundary values in China concerning the atmospheric release of N_r_, nitrogen runoff to surface water and leaching to groundwater are 14.6, 5.2 and 4.8 Tg N yr^−1^, respectively (Fig. 1a and Fig. S1). Notably, the estimated national boundary for nitrogen runoff aligns well with previous assessments of 4.4, 5.2 and 6.5 Tg N yr^−1^, all of which applied the same surface-water quality standard but employed different methodological approaches [16,17,20]. In 2020, intensive agricultural activities in China, together with excessive fertilizer application rates, resulted in a cropland NUE of ∼40%, which is substantially lower than the global average of ∼55% [23]. The combination of low agricultural NUE and an insufficient waste-treatment infrastructure led to substantial releases of N_r_ into the atmosphere and aquatic systems (Fig. 1b and Figs S2–S5) that exceeded the safe nitrogen boundaries across many regions (Fig. S3). Interestingly, due to considerable spatial heterogeneity in boundary exceedance, the cumulative remaining gaps from regions with relatively low N_r_ loss contribute to the national aggregated boundary value, thereby creating a ‘falsely’ elevated boundary at the national scale (Fig. 1a). That is to say, when county-level boundaries are aggregated, counties that do not exceed their boundaries still add their remaining unexploited boundary ‘gaps’, further inflating the aggregated value. As a result, the accumulated national boundary value appears larger than what is ecologically or practically meaningful at this scale. Restricting the analysis to only those regions in which the safe nitrogen boundaries were exceeded in 2020 reveals much more stringent limits: the aggregate safe boundaries for the atmospheric release of N_r_, nitrogen runoff and leaching decrease to 12.0, 2.9 and 1.8 Tg N yr^−1^, respectively. In these exceeded regions, the magnitude of transgression is particularly pronounced: cumulative N_r_ losses in 2020 were 54%, 262% and 258% higher than the corresponding aggregate safe boundaries for atmospheric release, runoff and leaching, respectively.

China’s terrestrial ecosystems exhibit lower sensitivity to acidification and eutrophication from atmospheric N_r_ deposition compared with those in Europe and the USA [24]. This reduced sensitivity is supported by several empirically documented mechanisms. First, the base cation deposition in China is reported to be an order of magnitude higher than those in Europe and the USA due to inputs from natural mineral dust and anthropogenic sources, providing substantial buffering capacity [24,25]. Second, long-term exposure to elevated nitrogen deposition has enhanced vegetation nitrogen uptake and increased plant nitrogen concentrations across major ecosystem types, thereby strengthening the overall nutrient retention and buffering capacity [26]. Third, soils in China’s ecosystems display stronger sulfate retention and higher denitrification capacity [24], further mitigating the ecological impacts of atmospheric N_r_ deposition. As a result, the critical loads established for terrestrial ecosystems in China, which are used to safeguard biodiversity, are higher than those reported in comparable studies elsewhere (Table S2 and Fig. S1). Nonetheless, in many regions with intensive agricultural or human activities, atmospheric N_r_ emissions still exceed their regional boundaries. Among the counties in which the atmospheric boundaries are surpassed, a reduction of ∼35% is required to bring emissions within the safe boundary (from 11.7 to 7.6 Tg N yr^−1^).

Compared with the atmospheric boundary, the regional boundaries for surface water and groundwater show far more severe exceedances due to both stricter environmental quality standards and high N_r_ losses (Figs S1b and c, and S5). These combined pressures impose a much greater challenge for maintaining water quality, with ∼85% of the population residing in areas where boundaries for surface water and groundwater are exceeded. Achieving compliance with regional water boundaries in these regions requires far greater reductions than those for air pollution—specifically, a 72% reduction in nitrogen runoff (from 5.3 to 1.5 Tg N yr^−1^) and a similar 73% reduction in nitrogen leaching (from 3.0 to 0.8 Tg N yr^−1^). The reduction required in China far exceeds those in other regions with intensive nitrogen use: central Europe requires a 54% reduction, India 44% and the USA 33%, while the global average reduction needed is only ∼15% [16].

The exceedance of all three nitrogen boundaries poses widespread and profound risks to natural ecosystems and human health. We found that 69% of China’s land area exceeds at least one of the three boundaries and 96% of the population (that is, 1.3 billion) resides within these affected regions (Fig. S3). Even more concerning is that areas in which all three boundaries are simultaneously exceeded are highly clustered in the North China Plain and the southeastern coastal zone. Although these hotspots account for only 17% of the national land area, they support ∼70% of the population (that is, 1.0 billion) (Fig. S3). This striking spatial concentration of multiple boundary exceedances highlights the urgent need for targeted and region-specific mitigation strategies. To further characterize the regional disparities in N_r_ losses, county-level results were categorized into six subregions based on geographical locations and administrative divisions. Substantial differences in the exceedance of safe nitrogen boundaries were observed between subregions (Fig. 1c–h), reflecting strong spatial variations in both N_r_ losses (Figs S4 and S5) and safe nitrogen boundaries (Fig. S1). In eastern and central-southern China, excessive fertilizer application in cropland, combined with high population density and the consequently large quantities of waste generated, leads to substantial N_r_ losses and significant exceedance of all three boundaries (Fig. 1e and f). An exception is noted for nitrogen runoff in central-southern China, where abundant precipitation and consequently high runoff yield elevated the critical loads for nitrogen runoff to surface water, resulting in some areas not exceeding this boundary even under high-discharge conditions (Fig. 1f). Additionally, southwestern and northwestern China are vast yet sparsely populated, leading to much lower exceedance levels. Figure 1g and h shows that the majority of the national remaining gap between current N_r_ losses and the safe nitrogen boundaries originates from these two regions.

### Mitigation potential and cost–benefit analysis

**Figure 2.**
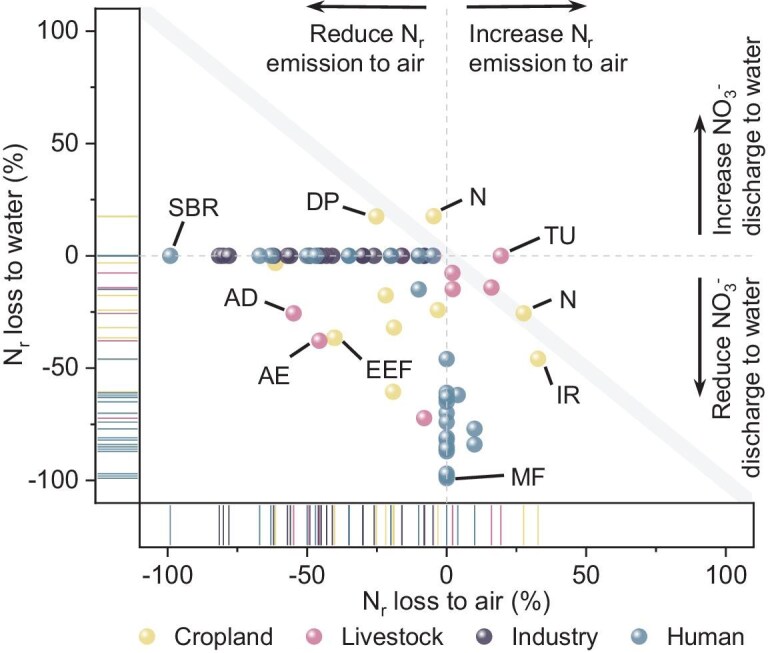
Abatement rates of all technical mitigation measures in each sector. Abatement rates for specific forms of N_r_ losses of each measure can be found in Table S5. SBR, Nitrification/Denitrification Sequencing Batch Reactor, AD: acidifiers or additives, AE: adjusted aeration modes; EEF, enhanced efficiency fertilizers; DP, deep placement; N, new cultivar; MF, membrane filtration; TU, turning of manure; IR, irrigation.

To identify feasible strategies for safeguarding regional nitrogen boundaries, we examined a wide range of technical mitigation measures, assessed their potential to reduce N_r_ emissions and evaluated their cost-effectiveness. A comprehensive set of 72 key technical N_r_-mitigation measures has been developed, encompassing four sectors and seven systems (Fig. 2 and Tables S4 and S5). Overall, most of the selected measures can synergistically reduce N_r_ emissions to both air and water by 10%–80% when implemented appropriately (Fig. 2). Several measures, including enhanced efficiency fertilizers (EEF), nitrification/denitrification sequencing batch reactors (SBRs), adjusted aeration modes (AE) and membrane filtration (MF), demonstrate particularly high mitigation performance. For example, EEF can reduce total N_r_ emissions by 40% while increasing crop yields by 7% and improving NUE by 44% (Figs S6 and S8). However, a subset of measures, such as no-tillage (T), deep placement (DP), new cultivars (N) and turning of manure piles (TU) that were introduced to address one pollution problem, may inadvertently exacerbate another [3], resulting in pollution swapping between air and water matrices [27,28]. For instance, deep placement of fertilizer can substantially reduce NH_3_ emissions by ∼64%, yet may concurrently increase nitrogen leaching to groundwater by ∼9% (Fig. 2). Such measures were therefore adopted with caution to avoid unintended trade-offs and environmental shifts, particularly in regions where the corresponding boundary values had already been exceeded (Section S1.6).

**Figure 3.**
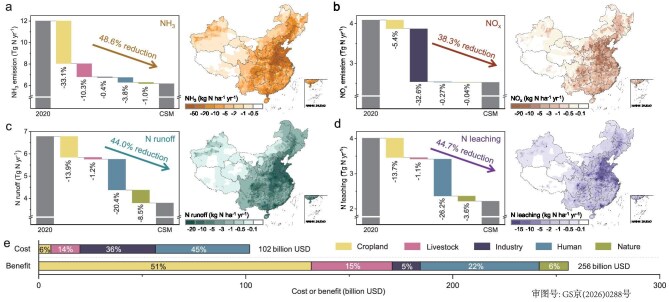
Mitigation potentials and cost–benefit analysis of cross-system technical abatement measures. For waterfall plots, total (a) NH_3_ emissions, (b) NO*_x_* emissions, (c) nitrogen runoff and (d) leaching in 2020 and under the CSM are shown, together with the reductions in N_r_ losses contributed by different sectors. The spatial distribution of N_r_ reductions is illustrated in the accompanying maps. (e) Total costs and benefits of mitigation measures across different sectors in China.

The overall N_r_-mitigation potential across China in 2020 was determined by using the abatement efficiency and the implementation rate of each measure. To ensure feasibility, we adjusted the implementation rates of various measures based on the extent of boundary exceedance and regional socioeconomic conditions (Section S1.6). By integrating all cross-system technical N_r_-mitigation measures into the coupled human and natural systems (CHANS) nitrogen-cycling model [29], we found that the N_r_ pollution in China could be reduced by an impressive 46% (12.2 Tg N yr^−1^) through the CSM (Fig. 3a–d). Among all sectors, the cropland sector shows the greatest mitigation potential, with the combination of all selected measures including 4R nutrient stewardship, nitrification inhibitors, cropping system adjustments and improved biophysical management capable of reducing N_r_ emissions by 53%, which accounts for 21% of the total N_r_ emissions. Similarly, mitigation measures can reduce N_r_ emissions from the livestock sector by 41%, equivalent to 5% of the total N_r_ emissions. These emission reductions are achieved alongside modest gains in agricultural productivity, with crop and animal yields in China projected to increase by 2.2% and 2.1%, respectively (Fig. S8). The industry and human sectors play crucial roles in reducing NO*_x_* emissions and NO_3_^−^ discharge to water, respectively, contributing to a 5% and 11% reduction in total N_r_ losses. Meanwhile, in the industry sector, measures including combustion modification and process improvement aimed at reducing NO*_x_* emissions also have the potential to boost industrial capacity, thereby increasing output and generating additional benefits. Additionally, a reduction in the atmospheric release of N_r_ under the CSM leads to a significant decline in nitrogen deposition on natural systems such as grasslands, forests and surface waters, resulting in a 3% reduction in nitrogen runoff from the nature sector (Fig. 3c and d). It is noteworthy that the reduction in N_r_ emissions varied spatially across China (Fig. 3a–d): the largest reductions (>50 kg N ha^−1^) were observed in the North China Plain, the Lower Yangtze River Plain, the Sichuan Basin and the Pearl River Delta, indicating regions of extremely high N_r_ losses. In contrast, the Qinghai-Tibet Plateau experienced lower reductions (<10 kg N ha^−1^) due to minimal human activity and N_r_ emissions in the area.

We estimated the net mitigation costs of the CSM, including material, labor and operational expenses, to be 102 billion USD. However, measures under the CSM can yield 245 billion USD of benefits (avoided damage costs) to ecosystems, human health and climate change from reduced N_r_ emissions, alongside an additional 11 billion USD yield in gains from the cropland and livestock sectors (Fig. 3e and Fig. S9). Spatially, the implementation costs are highest in densely populated regions such as the Yangtze River Delta and the Pearl River Delta, whereas the hotspots of monetized benefits are concentrated in coastal provinces including Shandong, Jiangsu and Guangdong (Fig. S10). Notably, these benefit hotspots show substantial spatial overlap with areas of high implementation costs. Overall, the total benefits are ∼2.5 times greater than the abatement expenses, demonstrating that the implementation of these mitigation measures is economically viable.

A sectoral comparison further highlights substantial differences in cost-effectiveness. Mitigation measures in the cropland sector alone generate a considerable benefit of 132 billion USD at the expense of only 6 billion USD. By contrast, further reductions in NO*_x_* emissions from the industrial sector are more expensive. Due to the extensive policy and regulatory frameworks governing industrial emissions in China, many cost-effective mitigation opportunities have already been implemented [30], meaning that additional reductions require disproportionately higher costs. Specifically, the industrial sector accounts for 36% of the total abatement expenses (36 billion USD), but delivers only 5% of the overall mitigation benefits (14 billion USD), underscoring the challenges faced in achieving further N_r_ reductions within the industrial sector. Moreover, for the human sector, mitigation measures primarily involve reducing N_r_ losses associated with waste disposal. The benefits mainly arise from two sources: (i) lower N_r_ discharge due to higher treatment efficiency and (ii) reduced treatment costs enabled by advanced technologies and equipment. Importantly, except for the industrial sector, all of the other sectors generate benefits that fully offset their mitigation costs.

### Different effects on air and water boundaries

**Figure 4.**
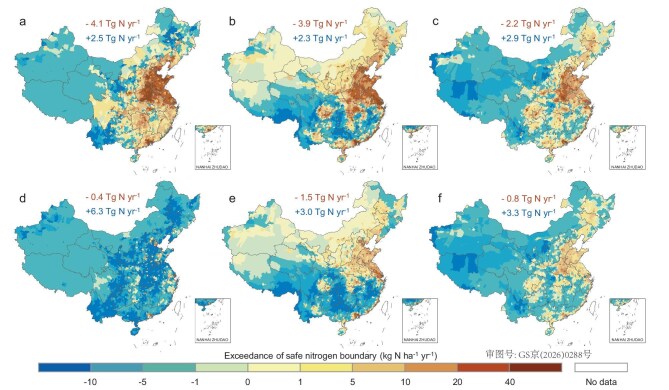
Spatial variation of safe nitrogen boundary exceedances at the county level in 2020 and under the CSM. (a–c) Exceedance of critical atmospheric release of N_r_, nitrogen runoff to surface water and leaching to groundwater in 2020, respectively; (d–f) corresponding exceedances under the CSM. Numbers above maps indicate the cumulative total exceedance where the respective boundary is exceeded (upper value) and the cumulative total remaining gaps where the boundary remains unexceeded (lower value).

While safeguarding regional nitrogen boundaries under the CSM, the exceedance patterns of air and water boundaries reveal a marked asymmetry (Fig. 4 and Fig. S11). In 2020, all three regional nitrogen boundaries were widely exceeded, with the atmospheric N_r_ emission boundary showing the most severe transgression across most regions. For instance, the exceedance for the atmospheric release of N_r_ reached >40 kg N ha^−1^ yr^−1^ in the North China Plain (Fig. 4a), whereas the reduction required for nitrogen runoff and leaching to comply with their corresponding boundaries was only ∼20 kg N ha^−1^ yr^−1^ (Fig. 4b and c). However, this situation becomes starkly different under the CSM scenario. Owing to more stringent criteria, nitrogen losses to surface water and groundwater remain seriously above their safe boundaries (exceedances of >5 kg N ha^−1^ yr^−1^) in many regions after reduction, particularly in densely populated or highly urbanized areas (Fig. 4e and f). In contrast, the same set of mitigation measures can successfully bring atmospheric N_r_ release below the regional boundaries in most parts of the country (Fig. 4d). This contrast suggests that, while air-pollution control appears achievable under the CSM, far greater challenges persist for improving the water quality in China.

**Figure 5.**
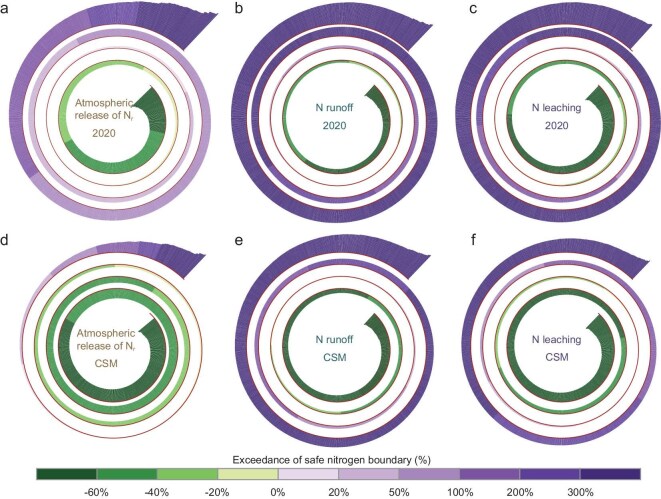
Number of counties exceeding safe nitrogen boundaries in 2020 and under the CSM. (a–c) Exceedance of critical atmospheric release of N_r_, nitrogen runoff to surface water and leaching to groundwater in 2020, respectively; (d–f) corresponding exceedances under the CSM. Each thin column represents a county; columns for counties that do not exceed the boundary point inward, while columns for counties that exceed the boundary point extend outward. The varying lengths of the columns signify different levels of exceedance.

As counties are the smallest administrative units in China and serve as the primary level for policy implementation and environmental management, quantifying safe boundaries at the county level rather than using watersheds helps to clarify more detailed environmental issues and local management options. Among 2847 counties, extensive exceedances of all three boundaries occurred in most of them (∼70% of the total) in 2020, particularly regarding nitrogen runoff to surface water (Fig. 5a and c). A total of 1263 counties even discharged N_r_ into surface water at levels that were more than three times the regional boundaries. Notably, under the CSM, N_r_ emissions could be reduced below the regional atmospheric boundary in 2251 counties, with only ∼20% failing to meet the safe limit, mainly due to exceptionally high population densities (Fig. 5d). However, for surface water and groundwater, boundary exceedances persist in over half of the counties even after reduction (Fig. 5e and f). There are 1316 and 1019 counties exceeding the surface-water and groundwater boundaries by >1-fold, respectively. This persistent exceedance implies that substantial additional efforts are imperative in these counties to meet the prescribed boundaries for water-pollution control.

### Co-benefits of safeguarding nitrogen boundaries

Efforts to safeguard nitrogen boundaries not only protect ecosystems and environmental quality, but also generate numerous co-benefits related to other planetary boundaries. While this study primarily focused on the mitigation potential of N_r_-loss forms associated with regional nitrogen boundaries, we also estimated a reduction of 0.12 Tg N yr^−1^ (11%) in N_2_O emissions under the CSM (Table S5). This reduction will contribute not only to mitigating global warming, but also preserving the stratospheric ozone layer [31,32]. Additionally, the intricate coupling of the carbon and nitrogen cycles implies that numerous measures aimed at reducing N_r_ losses also hold the potential to mitigate carbon-based greenhouse gas (GHG) emissions, primarily CO_2_ and CH_4_ [33]. For instance, transitioning to new-energy vehicles can simultaneously reduce NO*_x_* and CO_2_ emissions by replacing decentralized fossil-fuel combustion with green electricity. These synergistic effects and co-benefits of reducing GHG emissions through N_r_-abatement measures can help decelerate climate change, supporting the goals of limiting global warming to 1.5°C above preindustrial levels and achieving carbon neutrality [34,35].

Similar to the nitrogen cycle, the phosphorus cycle represents another component of biogeochemical flows that has been severely transgressed at both global and regional scales [14,36]. The N_r_-abatement measures examined in this study also hold substantial potential for reducing phosphorus losses to aquatic environments. For example, the practice of ‘4R nutrient stewardship’ in the cropland sector aligns phosphorus fertilizer application to crop requirements, thereby decreasing phosphorus loss from soils [37]. Likewise, ‘feeding strategies’ and improved ‘manure management’ in the livestock sector reduce phosphorus inputs by tailoring feed composition to animal nutritional needs, which in turn lowers the transfer of phosphorus from manure to the environment [36]. Therefore, efforts to safeguard regional nitrogen boundaries are also instrumental in maintaining regional phosphorus pollution within safe limits and reducing the risk of eutrophication in lakes, rivers and coastal areas.

Last but not least, the reduction in N_r_ losses to air and water under the CSM also helps alleviate pressures on three additional planetary boundaries, bringing multiple benefits: (i) decreasing atmospheric nitrogen deposition supports the protection of terrestrial biodiversity, thereby indirectly enhancing biosphere integrity; (ii) the reduction in N_r_ gas emissions (NH_3_ + NO*_x_*) limits the formation of PM_2.5_ by controlling key precursor substances [22], which is directly relevant to the planetary boundary concerning atmosphere aerosol loading [38]; (iii) improving surface water and groundwater quality enhances water availability by reducing blue water scarcity caused by pollution or eutrophication [39]. Taken together, these co-benefits demonstrate that the CSM plays an essential role in maintaining the resilience and stability of the Earth system while safeguarding multiple planetary boundaries [13,38].

### Policy implications

Effective nitrogen governance requires policy frameworks that are firmly grounded in a scientific understanding of spatial heterogeneity and environmental thresholds. The pronounced regional variation identified in this study underscores the importance of defining nitrogen boundaries at spatial scales relevant to biophysical constraints. County-level nitrogen boundaries provide a practical and policy-relevant basis for differentiated management, allowing mitigation requirements to be aligned with local environmental pressures and implementation capacity, and avoiding measures that are either overly stringent or insufficiently ambitious. Moreover, defining boundaries based on nitrogen losses rather than inputs directly targets environmentally harmful nitrogen fluxes and reduces the ambiguity arising from regional differences in NUE (Table S1), thereby offering a clearer foundation for policy design.

As one of the leading global risk factors for mortality, air pollution affects millions of people worldwide and is associated with severe cardiovascular and respiratory diseases, cancers and other health conditions [40]. To mitigate air pollution and its associated health risks, China has progressively strengthened its air-quality governance since the 1980s, with particularly stringent policy actions implemented over the past decade, including the Cleaner Production Promotion Law in 2012 [41], the Atmospheric Ten Actions in 2013 [42], the Zero Increase Action Plan for fertilizer use since 2015 [43] and the 3-year Action Plan for Winning the Blue-Sky Defense Battle in 2018 [44]. These policies, together with the enforcement of ambient air-quality standards [45,46], have been effective in reducing NO*_x_* emissions from the energy, industry and transport sectors [30,47], leading to marked declines in nitrogen deposition and PM_2.5_ concentrations as documented by monitoring networks, emission inventories and model simulations [45,48–51]. Notably, agricultural NH_3_ emissions have recently been incorporated into mitigation targets in several hotspot regions, reflecting the growing recognition of their role in air-quality improvement [42,52,53], and this inclusion has been shown to further reduce atmospheric PM_2.5_ concentrations [54,55]. Against this backdrop, the substantial mitigation potential identified under the CSM, particularly in the cropland and livestock sectors, outlines a clear pathway for reducing atmospheric N_r_ emissions to within safe boundaries. Realizing this potential will require continued efforts to remove socioeconomic barriers and to promote the widespread implementation of effective mitigation measures and related policies.

In contrast to the relatively greater feasibility of air-quality improvement, China’s experience highlights the need for more comprehensive and transformative strategies to safeguard water-related nitrogen boundaries. Despite the implementation of policies such as the Law of China on Prevention and Control of Water Pollution [56] and the National Groundwater Pollution Prevention and Control Plan [57], maintaining water pollution within safe limits remains difficult in many regions, with unintended consequences for aquatic ecosystems and human health. One important institutional constraint is that responsibilities for controlling agricultural runoff, rural sanitation and industrial wastewater are often dispersed across multiple agencies and governance levels, which hampers coordination and weakens the effectiveness of integrated nitrogen control. Moreover, the hydrological characteristics of nitrogen transport further exacerbate the difficulty of managing aqueous nitrogen pollution. Nitrogen moves through multiple flow paths, with residence times ranging from rapid surface runoff to groundwater transport spanning years or even decades. Consequently, the present nitrogen loads in many rivers partly reflect inputs from past decades, creating substantial time lags between mitigation actions and measurable improvements in water quality [58]. These legacy effects and long travel times make it inherently more challenging to bring aqueous nitrogen within safe boundaries compared with atmospheric nitrogen, underscoring the need for long-term, integrated water-management strategies.

To thoroughly address China’s water-pollution challenges, socioeconomic transformations are indispensable complements to technical measures, especially in regions where the CSM alone cannot bring nitrogen losses within the regional nitrogen boundaries. Firstly, such a transformation requires shifts in dietary habits, particularly in densely populated urban areas, by encouraging the reduced consumption of animal protein [59,60]. Secondly, reducing food waste, curbing overconsumption and minimizing waste along the supply chain can effectively lower N_r_ release from human activities [61]. Additionally, in regions with intensive agricultural activities that contribute large amounts of N_r_ to water, recoupling croplands and livestock can mitigate N_r_ losses and reduce the costs associated with storing and transporting animal manure. Furthermore, the coordinated management of atmospheric N_r_ emissions is essential for synergistically reducing nitrogen losses to water originating from nitrogen deposition [47,62]. Establishing an integrated nitrogen-management system that links air and water governance is critical for safeguarding all regional nitrogen boundaries. At the same time, such an integrated approach must also pay attention to avoiding unintended trade-offs across coupled biogeochemical cycles, ensuring that progress in nitrogen control does not inadvertently exacerbate issues related to carbon, phosphorus, acidity, soil-nutrient accumulation or other environmental pressures.

The remarkable mitigation potential revealed by the N_r_-mitigation measures in this study offers a promising blueprint for strengthening pollution control in China. Nonetheless, the thorough implementation of these measures remains difficult because of multiple socioeconomic barriers. In the cropland sector, although many management practices have already demonstrated high cost-effectiveness, their adoption remains limited. This limited adoption is largely due to the prevalence of small-scale farming [63] and the aging rural population [64], which together constrain the capacity for the widespread application of improved nutrient management. Furthermore, it is important to acknowledge potential rebound effects, as the enhanced crop yields from management practices could inadvertently incentivize agricultural expansion or intensification, which may erode a portion of the projected nitrogen-reduction gains [65,66]. In the livestock sector, these challenges are further compounded by the persistently low manure-recovery rate in many regions, where insufficient collection and recycling infrastructure continues to contribute to substantial nutrient losses [67]. Also, for industrial and human sectors, early progress in controlling point-source pollution has already captured many of the low-cost mitigation opportunities [68,69]. Further reductions increasingly depend on technological innovation, which often potentially requires more substantial financial investment and higher implementation costs [70].

Beyond these socioeconomic and implementation-related constraints, several sources of uncertainty and limitation in the analysis itself should also be acknowledged. First, nitrogen budget calculations carry inherent uncertainties because they rely on the integration of multiple datasets and require the simplification of complex biogeochemical processes. These simplifications may obscure substantial regional differences in soils, climate and agricultural practices, which can lead to either overestimation or underestimation of the nitrogen inputs and losses. Second, additional uncertainties arise when estimating nitrogen losses under the CSM scenario. Evidence from meta-analyses and literature reviews demonstrates that nearly all mitigation measures exhibit considerable variability across soil and climate conditions, equipment availability and management practices, particularly for those targeting the cropland and livestock sectors (Figs S6 and S7, and Table S5). These inherent regional differences naturally translate into pronounced spatial heterogeneity during real-world implementation. Third, the derivation of safe nitrogen boundaries involves uncertainty because ecosystems differ markedly in their sensitivity to nitrogen enrichment and it remains difficult to apply threshold values across diverse terrestrial and aquatic environments in which ecological responses vary substantially. Fourth, estimating nitrogen trajectories through atmospheric and hydrological pathways remains challenging because they involve complex chemical transformations, spatially variable deposition patterns and hydrological uncertainties that are particularly pronounced in regions with limited activity data. Taken together, these uncertainties highlight the importance of improving region-specific observations, expanding high-quality datasets and strengthening adaptive management frameworks to refine future nitrogen-mitigation assessments.

Overall, the regional nitrogen boundaries quantified in this study, together with the revealed asymmetry of safeguarding regional air and water nitrogen boundaries, provide a robust scientific foundation for guiding China’s transition toward sustainable nitrogen management. By linking biophysical thresholds with county-level assessments of mitigation potential and costs, this framework supports the design of mitigation pathways that are both environmentally effective and economically feasible. More importantly, the regional targets proposed here can anchor a long-term governance system that aligns national development priorities with global sustainability commitments, thereby contributing to the stabilization of Earth-system processes and advancing the collective pursuit of a safe operating space for humanity.

## METHODS

The assessment of the regional nitrogen boundary exceedances was conducted by comparing the county-level nitrogen losses and county-level safe nitrogen boundaries. Data sources are described in Section S1.1.

### Regional nitrogen boundaries

County-scale safe nitrogen boundaries were established based on four criteria (Section S1.2): (i) critical nitrogen-deposition rates to prevent terrestrial biodiversity loss, (ii) the atmospheric NH₃ emission threshold (31 kg NH₃-N ha^−1^ yr^−1^, Section S1.3), (iii) the surface-water nitrogen concentrations limit (1.0 mg N L^−1^) to prevent eutrophication [10,15] and (iv) the groundwater NO_3_^−^ concentration limit was set at 11.3 mg NO_3_^−^-N L⁻¹, following both WHO and Chinese drinking-water standards [71,72]. Given that the focus of this study was on regional impacts, boundaries for N_2_O emissions were excluded, as N_2_O exerts global rather than regional effects and is more pertinent to climate and ozone boundaries than to the nitrogen cycle [15].

### Nitrogen budget estimation

Nitrogen losses to air and water were estimated for 2847 counties in China by using the CHANS model (Section S2), which simulates nitrogen flows across 14 subsystems (e.g. cropland, livestock, forest, industry) based on mass balance principles. The calculation is described by Equation (1):


(1) \begin{eqnarray*}
\mathop \sum \limits_{k{\mathrm{ = 1}}}^p AC{C}_k = \mathop \sum \limits_{{\mathrm{h = 1}}}^m I{N}_{\mathrm{h}}{\mathrm{-}}\mathop \sum \limits_{g{\mathrm{ = 1}}}^n OU\!{T}_g.
\end{eqnarray*}

Detailed calculations are provided in Sections S1.4 and S2.

### CSM

A comprehensive library of nitrogen-mitigation measures was compiled through a systematic literature review (Section S1.5). In total, 4110 observations from 734 references were categorized into 22 strategies comprising 72 measures spanning the cropland, livestock, industry and human sectors (Table S4). Measures were selected based on their high abatement efficiency, co-benefits, low implementation costs and practical feasibility.

To estimate the regional N_r_-mitigation potential, a mitigation scenario referred to as the CSM was constructed. The CSM framework integrates coordinated technical measures across all sectors to tackle the major sources of N_r_ losses. It adjusts the implementation rates in response to regional boundary exceedances and economic affordability (Section S1.6). The abatement potential was quantified by incorporating nitrogen fluxes, measure-specific mitigation efficiencies and adoption rates reflecting realistic feasibility into the CHANS model.

### Cost–benefit analysis

The implementation costs of the mitigation measures included investment expenditures together with fixed and variable operating costs, drawing upon the Greenhouse Gas and Air Pollution Interactions and Synergies (GAINS) model [73] and recent literature (Table S5). Costs were calculated at the county level by using sector-specific activity data (e.g. cropland area, livestock numbers) and were expressed in constant 2020 USD (Section S1.7).

Benefits encompassed avoided damages to ecosystems, improvements in human health, climate-related gains and increases in agricultural yield (Section S1.7). Ecosystem benefits were estimated by adapting damage costs from the USA using China-specific willingness-to-pay and gross domestic product (GDP) data (Table S8 and S9). Health benefits were quantified based on the avoided mortality associated with reductions in PM_2.5_ concentrations driven by lower N_r_ emissions. Climate benefits reflected the cooling effects associated with reductions in NO*_x_* and NH_3_ emissions. Yield benefits were calculated from projected increases in agricultural production combined with national market-price data.

## Supplementary Material

nwag113_Supplemental_File

## Data Availability

Data supporting the findings of this study are available within the article and its supplementary information files or are available from the corresponding author upon reasonable request.
