# Supplementary material for: Asymmetry of safeguarding regional air and water nitrogen boundaries in China
Source: Natl Sci Rev. 2026 Feb 16;13(6):nwag113. doi: 10.1093/nsr/nwag113 (PMC13032868; doi:10.1093/nsr/nwag113)
Supplement: nwag113_Supplemental_File [file nwag113_supplemental_file.pdf]

Supplementary information to

Asymmetry of safeguarding regional air and water nitrogen boundaries in China

Yiyang Zou[1,2], Xiuming Zhang[3], Xin Xu[1,2], Jiami Wu[2], Luxi Cheng[1,2], Xinpeng Xu[4], Ouping Deng[5], Yuanyuan Chen[1,2], Chen Wang[1,2], Peiying He[1,2], Sitong Wang[1,2], Mengru Wang[6], Wilfried Winiwarter[3,7], Baojing Gu[1,2,8*]

[1] State Key Laboratory of Soil Pollution Control and Safety, Zhejiang University, Hangzhou 310058, China
[2] College of Environmental & Resource Sciences, Zhejiang University, Hangzhou, 310058, China.
[3] International Institute for Applied Systems Analysis, Schlossplatz 1, A-2361, Laxenburg, Austria.
[4] School of Earth Sciences, Zhejiang University, Hangzhou, 310058, China.
[5] College of Resources, Sichuan Agricultural University, Chengdu 611130, China.
[6] Earth Systems and Global Change Group, Wageningen University & Research, Droevendaalsesteeg 3, 6708 PB, Wageningen, the Netherlands
[7] Institute of Environmental Engineering, University of Zielona Góra, Licealna 9, PL 65-417, Zielona Góra, Poland.
[8] Policy Simulation Laboratory, Zhejiang University, Hangzhou, 310058, China.

***Corresponding Author:**
College of Environmental & Resource Sciences, Zhejiang University, 866 Yuhangtang Road, Hangzhou 310058, China. Tel & Fax: +86 571 8820 6926. E-mail: bjgu@zju.edu.cn

S1. Introduction of methods

S1.1 Analytical scale and data sources

The Coupled Human And Natural Systems (CHANS) nitrogen cycling model [1] was employed to calculate the nitrogen budget at the county level in 2020. In determining the minimum analytical unit, this study adopts a county-based approach, differing from many previous studies that used watersheds, river basins, or eco-geographical regions as the smallest research units [2]. The primary rationale for this choice is that counties constitute the smallest and most fundamental administrative units for policy implementation and environmental management in China. While watersheds, basins, or eco-geographical regions are often more consistent with hydrological or ecological processes, they typically encompass multiple counties that operate under distinct governance structures, administrative responsibilities, and management priorities. This administrative heterogeneity complicates the coordination and enforcement of uniform nitrogen management measures across jurisdictions within the same watershed or basin, thereby limiting their direct applicability for policy design and implementation.

By contrast, nitrogen management policies in China, including agricultural regulation, pollution control, and infrastructure investment, are predominantly formulated, implemented, and evaluated at the county level. Accordingly, both nitrogen budget calculations and boundary assessments in this study were conducted using county-wide average values, reflecting the administrative scale at which nitrogen management interventions are realistically implemented. Although this approach may smooth localized variability in hydrological and ecological processes, it enables a consistent, policy-relevant, and nationally comparable assessment of nitrogen dynamics across diverse regions. Given that the primary objective of this study is to identify regional nitrogen boundary exceedances and to inform management frameworks rather than to resolve fine-scale hydrological and ecological processes, the county level was therefore selected as the minimum analytical unit.

For model construction, the required data are as follows: (1) China's national land use cover for 2020 (1km×1km) was derived from the Resource and Environment Data Cloud Platform (REDCP, https://www.resdc.cn/); (2) Activity data that used in CHANS model (e.g., crop yield, fishery production, industrial products) was derived from county-level census datasets published in the China County Yearbook 2021 (https://www.stats.gov.cn/sj/ndsj/); (3) Datasets for N_r loss like total nitrogen discharged from wastewater treatment plant were directly collected from Annual statistic report on environment in China, 2020 (https://www.mee.gov.cn/hjzl/sthjzk/sthjtjnb/index.shtml); (4) County-scale population data was extracted from a 100-m gridded population dataset of China's seventh census in 2020 [3]; (5) County-scale GDP was extracted from a 1km×1km gridded dataset using nighttime lights time series and population images for spatial GDP estimation [4]; (6) The geo-distribution of livestock was sourced from the Agricultural Pollution Source Census of China in 2017 (APSCC2017) [5]; (7) Various nitrogen process parameters (e.g., nitrogen concentrations in grain and straw, nitrogen contents of major nitrogen-containing industrial products, human excretion fate) and N_r emission factors (EFs) used in CHANS model was derived from Gu et al., 2015 [1] and latest published field experiments.

For the estimation of regional nitrogen boundaries at the county level, the required data are as follows: (1) Atmospheric inorganic nitrogen deposition (including dry and wet deposition) was by integrating several dataset and of China's nitrogen deposition fluxes at pixel levels [6-8]. (2) The hydrometeorological data including total precipitation, runoff, and evapotranspiration was obtained from ERA5-Land

(https://cds.climate.copernicus.eu/cdsapp#!/dataset/reanalysis-era5-land-monthly-means?tab=overview) at pixel levels. Given data availability and to represent long-term average conditions, yearly mean values from 1950 to 2022 were used. All pixel-level data were subsequently aggregated to the county scale, and all statistical analyses were conducted at this administrative level.

S1.2 Quantification of regional boundaries

Regional nitrogen boundaries were determined based on the following critical limits: (1) atmospheric nitrogen deposition in natural terrestrial ecosystems to prevent acidification and biodiversity loss. (2) NH_3 emission threshold for protecting human respiratory system and health; (3) nitrogen concentrations limits in surface waters to control eutrophication. (4) nitrogen concentration standards in groundwater aligned with drinking water quality to avoid adverse human health effects. The exceedance of these boundaries was assessed by comparing aggregated emissions against the boundary values at the county level, rather than allocating predetermined thresholds among different sources. This approach ensures a comprehensive evaluation of nitrogen load impacts across regions without artificially dividing limits across sectors.

Critical atmospheric nitrogen deposition rates associated with acidification and biodiversity loss in terrestrial ecosystems were established for each of the 12 vegetation types in China (including emperate coniferous forest, subtropical coniferous forest, warm mixed forest, temperate deciduous forest, subtropical evergreen broad-leaved forest, subtropical monsoon evergreen broad-leaved forest, steppe and grassland, tropical plantation, scrubland, fens, alpine meadow and tundra, and cropland). Excessive nitrogen deposition onto terrestrial ecosystems may induce changes in species composition and soil microorganisms, potentially altering native species and mycorrhizal communities. The sensitivity to nitrogen deposition varies significantly among different vegetation types. In order to account for these differences, critical loads for each vegetation type were based on a synthesis of studies that used empirical values or the steady state mass balance (SSMB) method. In China, critical nitrogen deposition rates range from 7.5 kg N ha^{-1} yr^{-1} for the most sensitive vegetation type to 40 kg N ha^{-1} yr^{-1} for cropland. Specific values for 12 vegetation types and their main responses for exceedance can be found in Table S2. Higher critical loads were established for tropical vegetation types and cropland in this study due to their relative insensitivity to nitrogen deposition [9]. Besides, nitrogen deposition may serve as an important contribution to nutrient budgets in intensively managed croplands [10, 11].

In addition, the atmospheric release of N_r is considered potentially detrimental to air quality and poses significant risks to public health [12, 13]. It was suggested by Rockström et al. [14] that NH_3 can function as a critical air pollutant within the framework of Earth system boundaries. Therefore, we articulated the constraint on air quality through the intensity of NH_3 emissions. In this study, we established a standard for NH_3 emission at 31 kg NH_3-N ha^{-1} yr^{-1} (The detailed derivation process can be found in Section S1.3) by integrating: (1) the secondary standard for $PM_{2.5}$ pollution in China's ambient air quality standards [15] of 35 μg/m^3; (2) criteria related to ammonia scoring aimed at achieving Sustainable Development Goal 2 [16]; and (3) standards employed in published peer-reviewed studies [17, 18]. Regulating NH_3 emission intensity below this threshold can significantly reduce the global burden of respiratory and cardiovascular diseases associated with $PM_{2.5}$ pollution.

Critical levels of atmospheric release of N_r (NH_3+NO_x) were determined by both nitrogen deposition and NH_3 emission threshold (Section S1.3). Since the nitrogen deposition does not

equal emissions due to trans-regional transport [19, 20], we considered the transportation to downwind direction and stimulated this process by introducing a transfer coefficient (t_i) between N_r gas emission and deposition. Furthermore, to aggregate the two standards mentioned above, the contribution of NH_3 to total N_r gas (NH_3+NO_x) emission was assumed to be constant in this study. Critical atmospheric release of N_r for county i are thus calculated as:

$$N_{et,i} = N_{dt,i}/t_i \quad (1)$$

$$N_{em,crit,i} = N_{et,i} \times S_i \quad (2)$$

where $N_{et,i}$ refers to the emission threshold for atmospheric release of N_r (NH_3+NO_x) (kg N ha^{-1} yr^{-1}). The lower limit was set to 5 kg N ha^{-1} yr^{-1} to avoid extreme low values, while the upper limit was constrained by the previously mentioned criterion of 31 kg NH_3-N ha^{-1} yr^{-1} for NH_3 emission (we assumed that changes in NO_x emissions occur proportionally to those of NH_3 emissions within each county); $N_{dt,i}$ denotes critical value for nitrogen deposition threshold (kg N ha^{-1} yr^{-1}) based on the main vegetation type in county i (Table S2), and area-weighted average value was adopted if more than one vegetation types exist within a single county. t_i is the transfer coefficient which simulates the atmospheric transport process based on empirical values of atmospheric release of N_r and total nitrogen deposition (including both dry and wet deposition) in county i [19-21], and this parameter is assumed to remain constant for each specific county. $N_{em,crit,i}$ is the critical load for atmospheric release of N_r (kg N yr^{-1}). S_i refers to the total land area of county i (ha).

For surface water, critical nitrogen concentration to prevent eutrophication was established at 1.0 mg N L^{-1} based on (1) China's water-quality standards (Class III) for Total Nitrogen (TN) [22], (2) a comprehensive literature review focusing on the ecological and toxicological effects of nitrogen pollution [23-25]. Multiple studies have demonstrated that on average 50% of the nitrogen runoff discharged into surface water is removed during transport through retention, sedimentation and denitrification processes [26-29]. Consequently, this study employed a threshold of 2.0 mg N L^{-1} for nitrogen concentration in runoff as a proxy for nitrogen concentration in surface water. Besides, we postulated that if the nitrogen concentration in runoff doesn't exceed the threshold, nitrogen pollution in surface water would not breach the threshold. With the data of total runoff, the critical nitrogen runoff for county i could be calculated using Equation (3):

$$N_{sw,crit,i} = [N]_{runoff} \times W_{runoff,i} \times S_i \quad (3)$$

where $N_{sw,crit,i}$ refers to the critical nitrogen runoff to surface water (kg N yr^{-1}); $[N]_{runoff}$ is the critical nitrogen concentration in runoff to surface water (2.0 mg N L^{-1}); $W_{runoff,i}$ represents the average runoff in county i (unit: m, including surface runoff and sub-surface runoff). To avoid extremely low values for critical nitrogen runoff, we set a minimum runoff at 10% of the precipitation [30]. S_i refers to the total land area of county i (ha).

For groundwater, the critical nitrogen concentration to mitigate potential human health risks was established at 50 mg NO_3^- L^{-1} or 11.3 mg NO_3^--N L^{-1} based on (1) WHO guidelines for drinking water quality [31]; (2) EU Nitrates Directive [32]; (3) National standards for drinking water quality in China [33] (10 mg NO_3^--N L^{-1}, which aligns closely with standards (1) and (2)). The concentration in leachate itself can serve as a proxy for NO_3^- concentration in groundwater. Similar to runoff, we postulated that if the nitrogen concentration in leachate doesn't exceed the threshold, nitrogen pollution in groundwater would also remain below the critical level too. Critical nitrogen leaching for county i was calculated by multiplying the critical nitrogen concentration with the leached water volume, as follows:

$$N_{gw,crit,i} = [N]_{groundwater} \times W_{lea,i} \times S_i \quad (4)$$

$$W_{lea,i} = Pre_i - Evp_i - W_{runoff,i} \quad (5)$$

where $N_{gw,crit,i}$ refers to the critical nitrogen leaching to groundwater (kg N yr^{-1}); $[N]_{groundwater}$ is the critical nitrogen concentration in leaching flux towards groundwater (11.3 mg NO_3^--N L^{-1}); $W_{lea,i}$ represents the average leaching water volume in county i (unit: m), calculated as precipitation minus evapotranspiration and surface runoff [34]; S_i refers to the total land area of county i; Pre_i and Evp_i represent the average total precipitation and evapotranspiration in county i (unit: m); and $W_{runoff,i}$ denotes the average runoff in county i (unit: m).

Considering that terrestrial biodiversity and water quality are the primary focus of this study, the impacts of N_2O emissions were not taken into account for the following two reasons: First, as a relatively inert gas compared to NH_3 and NO_x, N_2O remains stable in the atmosphere for approximately 114 years [35]. Therefore, we assumed that all N_2O would accumulate in the atmosphere post-emission and would not contribute to nitrogen deposition. Second, N_2O has a global effect rather than a regional impact because it mixes uniformly in the atmosphere [24]. It is more closely related to boundaries such as climate change and stratospheric ozone depletion than to the nitrogen cycle. Therefore, calculating a threshold for N_2O emission in China was beyond our scope of discussion. Nonetheless, it is crucial to recognize that as a potent greenhouse gas, the warming potential of N_2O is a critical factor in the overall climate assessment of reactive nitrogen. A previous study has already calculated global critical N_2O-N losses using radiative forcing relative to pre-industry levels of 1 W m^{-2} and 2.6 W m^{-2} (equal 0.8 Tg N_2O-N yr^{-1} and 5.3 Tg N_2O-N yr^{-1}) [24]. While this method provides valuable insights, it may not fully capture the complex interplay between N_2O's warming effect and the potential cooling influences of other reactive N_r species like NH_3 and NO_x [36], necessitating a more nuanced approach to accurately assess the net climate impact.

S1.3 Determination of NH_3 emission thresholds

Aside from referencing the standards employed in published peer-reviewed studies [37, 38], we also considered the impact of NH_3 emissions on $PM_{2.5}$ formation and their implications for Sustainable Development Goal (SDG) scores in establishing the NH_3 emission threshold.

The formation of $PM_{2.5}$ is influenced by a combination of anthropogenic activities and natural environmental factors. Key precursor substances include nitrogen oxides (NO_x), sulfur dioxide (SO_2), volatile organic compounds (VOCs), and ammonia (NH_3) [39]. These compounds undergo complex atmospheric chemical reactions, leading to the secondary formation of $PM_{2.5}$. Additionally, meteorological conditions such as temperature, humidity, wind speed, and precipitation play a crucial role in the dispersion and transformation of $PM_{2.5}$ in the atmosphere [40]. Therefore, inferring the regional threshold for atmospheric release of N_r from the $PM_{2.5}$ concentration standard is challenging. We acknowledge this limitation in our study. However, various studies have demonstrated that NH_3 is a dominant precursor substance in many regions worldwide [41-43]; thus, we utilized the $PM_{2.5}$ concentration standard to derive a preliminary estimate of the holistic NH_3 emission threshold.

The Weather Research and Forecasting model coupled with Chemistry (WRF-Chem) [44] was employed to assess the effects of N_r emissions under the air pollutant concentrations in 2020. Meteorological and chemical initial and lateral boundary conditions were derived from the National Center for Environmental Prediction Final (FNL) Analysis data, as well as the output of the Community Climate Model with chemistry (CAM-chem) [45, 46]. To minimize simulation drift and align with real-world conditions, meteorological fields were nudged every two days. Additionally, the Moderate Resolution Imaging Spectroradiometer (MODIS)

Land Cover Type (MCD12Q1) Version 6 was utilized to update static geographical data, with the base year of 2020. The model incorporates the Carbon-Bond Mechanism version Z (CBMZ) for gas-phase chemistry [47], coupled with the MOSAIC-4 bins aerosol scheme [48]. To enhance the simulation of secondary inorganic aerosols, we applied the heterogeneous sulfate formation scheme proposed by Chen et al. [49]. Emission sources in the model include biomass burning, biogenic emissions, natural dust, and anthropogenic activities. Hourly biomass burning emissions were sourced from the Fire Inventory from NCAR (FINN) version 1.5 [50]. Biogenic and natural dust emissions were calculated online using the Model of Emissions of Gases and Aerosols from Nature (MEGAN) [51] and the Goddard Global Ozone Chemistry Aerosol Transport (GOCART) module [52], respectively. The benchmark for anthropogenic emissions integrates several widely used emission inventories. The 2020 Multi-resolution Emission Inventory for China (MEIC), with a spatial resolution of 0.25°, was employed across Chinese territory, except for NH_3 and NO_x emissions, which were estimated in this study. Emissions in other regions were sourced from the 2010 MIX Asia emission inventory [53]. The $PM_{2.5}$ concentration across China in 2020 obtained through model simulation is shown in Fig. S17a.

To present a preliminary assessment of the relationship between NH_3 emissions and $PM_{2.5}$ concentration, we utilized the average NH_3 emission intensity and $PM_{2.5}$ concentration across all counties for a simplified non-linear regression (quadratic fit) analysis. The regression equation obtained is expressed as $PM_{2.5}$ concentration = -0.001× NH_3 emission intensity^2 + 0.523× NH_3 emission intensity + 19.4 (Fig. S17b). Based on the secondary standard for $PM_{2.5}$ pollution in China's ambient air quality standards of 35 μg/m^3, it can be inferred that the corresponding NH_3 emission intensity is approximately 31 kg NH_3-N ha^{-1} yr^{-1}. Although numerous other environmental factors and atmospheric transport mechanisms exist, it can be reasonably assumed that if ammonia emissions in each region do not exceed this threshold, the risk of $PM_{2.5}$ concentrations exceeding regulatory standards will be significantly reduced, provided that other conditions remain unchanged.

In addition, we referred to the SDG report to establish NH_3 emission thresholds. The European Sustainable Development Report [54] provided data on EU SDG index scores and ammonia emission intensity from agriculture for each country. We utilized data sources that are clearly marked for the years 2015-2021 to ensure accuracy and conducted regression analysis while controlling for country as a fixed effect to establish the relationship between ammonia emissions and SDG scores. The statistical model employed was: ammonia score = -0.69 NH_3 + 87.83 (R^2 = 0.996), where the ammonia score represents an index score indicating SDG 2 — sustainable and resilient food production systems; and NH_3 denotes the amount of ammonia emissions resulting from agricultural production (kg ha^{-1} yr^{-1}). We assumed that the SDG report designated a score of 60 as the threshold for all countries. According to this standard, the threshold for NH_3 emissions can be calculated to be approximately 40 kg NH_3 ha^{-1} yr^{-1} (equivalent to 33 kg NH_3-N ha^{-1} yr^{-1}).

By combining these criteria with the threshold value of 31 kg NH_3-N ha^{-1} yr^{-1} proposed in peer-reviewed studies, we ultimately adopted 31 kg NH_3-N ha^{-1} yr^{-1} as the limit for ammonia emission standards in this study.

S1.4 Nitrogen losses to air and water

To estimate the fate of nitrogen losses in 2020 across 2,847 counties, the CHANS model was used. This model is a nitrogen-flow process-based model that stimulates the biogeochemical processes of nitrogen cycling. The whole system was divided into 14 subsystems (e.g.,

cropland, livestock, grassland, forest, human, industry, etc.) in the CHANS model (Fig. S12), each governed by the principle of mass balance:

$$\sum_{k=1}^{p} ACC_k = \sum_{h=1}^{m} IN_h - \sum_{g=1}^{n} OUT_g \quad (6)$$

where IN_h and OUT_g refer to nitrogen inputs and outputs (Tg N yr^{-1}), respectively, while ACC_k represents nitrogen accumulation (Tg N yr^{-1}). The subscript k denotes the various nitrogen fluxes that contribute to nitrogen accumulation, whereas h and g correspond to the nitrogen fluxes associated with inputs and outputs in each system. The nitrogen dynamics of each system, including accumulation, input, and output, are represented by the summation of several nitrogen fluxes, each indicated by a corresponding subscript. A more comprehensive description of the CHANS model framework and parameter construction could be found in Section S2.

Due to the data availability, some of the N_r losses (NH_3, NO_x, nitrogen runoff and leaching) to air and water were calculated at the provincial level and subsequently assigned to counties using proxy parameters including land use, population and GDP distribution [55]. For example, grassland runoff could be calculated by spatial distribution of grassland; human household wastewater disposal was allocated according to the spatial distribution of the population. Besides, since industrial activities are highly correlated with GDP [55], industrial NH_3 emission was calculated by using the spatial distribution of GDP. All proxy parameters for N_r losses, along with their selection criteria, are detailed in Figs. S13-S16 and Table S3. Specially, the geo-distribution for chemical fertilizer and manure application on cropland was derived from Wang et al. 2021 [56] at county level because of the large amount of N_r losses. In addition, since the distribution of livestock could not be estimated using any proxy parameters, we utilized the county-level results of Zhu et al. 2022 [5] for N_r losses from livestock subsystem. Nitrogen budget for each county is provided in Supplementary Data 1.

S1.5 Compilation of technical mitigation measures

To establish a comprehensive library of technical measures aimed at reducing N_r losses in China, we conducted a literature review of the publications after year 2000 from Web of Science (https://www.webofscience.com/wos/alldb/basic-search) and Google Scholar (https://scholar.google.com). To ensure practical feasibility, we considered only mitigation measures that are already in use. The keywords that we used in research included "nitrogen pollution OR ammonia OR nitrous oxide OR runoff OR leaching" "abatement OR mitigation OR reduction OR control", "cropland OR livestock OR industry OR transport OR wastewater OR household OR landfill". This process yielded 4,110 observations from 734 references and identified 22 strategies comprising 72 detailed measures. Importantly, the mitigation efficiencies summarized from the literature indicate broadly comparable reductions in both atmospheric and aqueous N_r losses, suggesting that the technical literature does not disproportionately emphasize air or water pollution control. All measures mainly aimed to reduce N_r losses from 7 different subsystems (as classified in the CHANS model, including cropland, livestock, industry, traffic, human, garbage and WTP) and were categorized into four sectors, encompassing cropland, livestock, industry and human (Fig. S12). Structural transition options such as low-meat diets and food waste reduction were not included in the CSM, as the scenario focuses exclusively on implementable technical mitigation measures. Detailed descriptions and abbreviations for each measure could be found in Table S4.

While selecting mitigation measures to reduce N_r losses to air (NH_3 and NO_x emissions) and water (nitrogen leaching and runoff), the following four aspects were considered:

1) High abatement efficiency: measures that could significantly reduce one or more kinds of N_r losses were prioritized. For example, adjusting manure aeration modes (AE) can notably

reduce NH_3 emissions by 70% without adverse effects on other emission types.

2) Synergies and limited trade-offs: measures capable of simultaneously reducing N_r losses to both air and water were given precedence. For instance, enhanced efficiency fertilizers (EEF) can synergistically reduce 45% and 29% N_r losses to air and water, respectively, while increasing crop yield by 7%. However, measures like deep placement, new cultivars, and irrigation that are designed to reduce one N_r pollution form may lead to another [57, 58]. To avoid the impact brought by pollution swapping as much as possible, we have sorted the adoption rates of these mitigation measures in descending order when the sensitive boundaries are exceeded (Table S6).

3) Low implementation cost: measures that are less expensive or labor-intensive are favored, such as legume rotation, no-tillage or dietary additive.

4) Practical applicability: measures with limited applicability due to technical, political, or socioeconomic barriers in China were excluded. For example, soil testing for optimized fertilizer use is only suitable for larger-scale farms in the US or Europe, this measure was not considered in this study due to its prohibitive costs for fragmented farmland in China.

For measures in the cropland and livestock sectors, abatement rates were derived from meta-analysis due to the availability of numerous experimental observations for each mitigation measure. The full set of data from the meta-analysis were detailed in Supplementary Data 2. The 10 abatement measures in the cropland sector can be categorized into four types: soil additives, 4R nutrient stewardship, crop species switching, and biophysical management. In the livestock sector, 10 mitigation measures are divided into three strategies: improved feeding, housing adaptation, and manure management. Every strategy includes a combination of two to four mitigation measures or practices.

A comprehensive meta-analysis was conducted on experimental experimental data from both cropland and livestock sectors to evaluate the effectiveness of N_r mitigation measures. Studies were incorporated if they fulfilled all of the following criteria (refer to the PRISMA (Preferred Reporting Items for Systematic Reviews and Meta-Analyses) flow diagram [59] illustrated as Fig. S18): (1) The sample means of nitrogen loss from specified pathways, yield, or NUE were documented for both control and treatment groups; (2) comprehensive details regarding experimental location, design, and conditions were provided to facilitate cross-checking of duplicate publications; and (3) only field experiments were considered relevant. Information pertaining to site locations, experimental settings, and variables was systematically extracted from texts, tables, and figures. Data from figures were extracted utilizing WebPlotDigitizer version 5.2 [60]. The natural logarithm of the response ratio of the observed values [61] ($lnR = ln(x_t/x_c)$, where x_t and x_c represent the mean values of the treatment and control groups, respectively) was used to evaluate the treatment effects of the management practices on nitrogen loss via various pathways (NH_3, NO_x, nitrogen runoff and leaching), crop or livestock yields and nitrogen use efficiency (NUE). Then, the weight of each observation was calculated based on the inverse of pooled variance and the number of experimental replicates [61-63]. For studies lacking reported standard deviations, inferences were made based on other experimental information to maintain data integrity. A randomized resampling approach and bootstrapping (4,999 iterations) [64] was adopted in this study with MetaWin 3.0.15 [65] to calculate the weighted bootstrap mean effect size (ES) and the 95% bootstrap confidence intervals (CIs). The results were presented as the percentage changes ($(ES - 1) \times 100$) under the management practices. Negative percentage changes denote a decrease in the variables due to management practices, while positive percentage changes indicate an increase. If the 95% bootstrap CIs didn't overlap with zero, effects of the management practices were considered significant [66].

Whereas, the effectiveness of mitigation measures for the industry and human sector was directly extracted or synthesized from reported literature results. For the industry sector, abatement measures primarily focus on NO_x reduction through process modification in industrial production or transportation. And measures in the human sector mostly aim to reduce waste generation during human consumption and activities. For example, using advanced toilets, improved landfill, or wastewater treatment techniques are the most commonly implemented measures for waste disposal.

S1.6 Cross-system nitrogen management

To estimate the regional N_r mitigation potential and cost-effectiveness of technical measures, we simulated nitrogen flux under Cross-System nitrogen Management (CSM). CSM integrates coordinated technical mitigation measures to address all major sources of N_r losses originating from various systems and adjusts their implementation rates based on regional socioeconomic factors. In this scenario, socioeconomic factors such as population, GDP, and urbanization rate were assumed to remain consistent with the 2020 baseline. For the estimation, we first parameterized the abatement rates of the mitigation measures and subsequently integrated them into the CHANS model. The calculation of the overall N_r mitigation potential ($\Delta E_{i,j,k}$) in county i is determined by the nitrogen flux, abatement efficiency and implementation rate of each measure:

$$\Delta E_{i,j,k} = NL_{i,j,k} \times \eta_{i,j,k} \times X_{i,j,k} \quad (7)$$

where j represents the form of N_r loss (NH_3, NO_x, nitrogen runoff and leaching); $NL_{i,j,k}$ is the N_r losses in county i in 2020 (Tg N yr^{-1}); $\eta_{i,j,k}$ is the abatement rate of the measure k (Table S5); $X_{i,j,k}$ is the implementation rate of the measure k (%).

Following the confirmation of mitigation measures and their effects, multiple factors influence the feasibility and necessity of their implementation. Given the disparities in emission control progress between developed and developing regions [67], the implementation rate of various measures was determined by: (1) the extent to which the corresponding boundary is exceeded (that means the boundary corresponding to the target N_r forms that can be reduced by emission reduction measure k) [20], which reflects the degree of environmental or regulatory urgency associated with nitrogen pollution control in a given region; (2) the affordability or income level of county i [21], which captures the economic and financial feasibility of adopting the proposed mitigation actions. The equation used for calculation is as follows:

$$X_{i,k} = \alpha_{i,k} \times \beta_{i,k} \quad (8)$$

where $X_{i,k}$ refers to the implementation rate of the measure k in county i (%); $\alpha_{i,k}$ is the adjustment coefficient based on boundary exceedance (Table S6); $\beta_{i,k}$ is the adjustment coefficient based on the income level (Table S6), with per capita gross domestic product (PGDP) used as a proxy in this study.

Moreover, in the industrial and human sectors, numerous mitigation measures exhibit inclusive or overlapping relationships (e.g., Low-NO_x burners and staged combustion), demonstrating comparable effectiveness. We selected the most suitable representative measure for each county rather than implementing all of them. For calculating the overall abatement potential, we employed strategy-combination effectiveness to approximate the integrated effect of all measures.

During the integration process, for measures without interactions, the reduction potential was

summed according to Equation (9) and (10) [68]. For interacting measures, results from experiments combining these measures were used to estimate the aggregate reduction potential:

$$\eta_{A+B} = \eta_A + (1-\eta_A)\eta_B \quad (9)$$

$$\eta_{A+B+C} = \eta_A + (1-\eta_A)\eta_B + [1-(\eta_A + (1-\eta_A)\eta_B)]\eta_C \quad (10)$$

where A, B, C are the different mitigation measures included in the combination; $\eta_{A,B,C}$ denote their respective abatement rates (%).

S1.7 Cost-benefit analysis

This study considers implementation costs from the perspective of strategy-implementing entities, encompassing investment costs (such as construction and equipment), fixed operational expenses, and variable operational costs (such as labor expenditures). Here, we mainly refer to the database and methodology for the assessments from the GAINS (Greenhouse Gas and Air Pollution Interactions and Synergies) model available online to estimate abatement costs at provincial-level. A comprehensive description of the GAINS model and its cost calculation module is provided by Klimont et al. 2011 [69]. Specific costs associated with each measure are derived from diverse reference materials, as detailed in Table S5. All costs in the calculations in this study are measured in constant 2020 USD. What's more, due to the variability in metric selection, corresponding unit costs for selected measures have both maximum and minimum options, with the average cost adopted for calculations. The annual implementation cost in county i (IC_i) is calculated based on Equations (11-13). It is important to note that Equations (11) and (12) represent two separate and alternative approaches, depending on the availability of data for each mitigation measure. Specifically, Equation (11) is applied when only the integrated unit abatement cost is available, while Equation (12) is used for measures where explicit unit implementation costs are provided, such as the cost per unit area of cropland or per head of livestock.

$$IC_{i,j,k} = \Delta E_{i,j,k} \times UAC_{i,k} \quad (11)$$

$$IC_{i,k} = A_i \times UIC_{i,k} \times X_{i,k} \quad (12)$$

$$IC_i = \sum\sum IC_{i,j,k} \quad (13)$$

in which j represents the term of N_r loss (NH_3, NO_x, N runoff and leaching); $IC_{i,j,k}$ refers to the implementation cost for measure k in county i ($). In Equation (11), $\Delta E_{i,j,k}$ represents the reduction of N_r emissions in different forms (kg N yr^{-1}), derived from the integrated modeling analysis presented in Equation (7); $UAC_{i,k}$ refers to the integrated unit abatement cost for mitigation option k ($), obtained from the GAINS model database and recent literature (Table S5). In Equation (12), A_i is the activity data such as cropland area, livestock number, garbage produced, sewage discharged, etc.; $UIC_{i,k}$ refers to the implementation cost of measure k, which is derived from the literature (Table S5). $X_{i,k}$ is the implementation rate of the specific measure, as presented in Equation (8).

The societal and economic benefits of mitigating N_r pollution ($SOC_{benefit,i,j}$) mainly encompass avoided damage costs of ecosystem health benefits ($EH_{benefit,i,j}$), human health benefits ($HH_{benefit,i,j}$), climate benefits ($CL_{benefit,i,j}$) and crop/animal yield benefits ($YD_{benefit,i,j}$), as shown in Equation (14):

$$SOC_{benefit,i,j} = EH_{benefit,i,j} + HH_{benefit,i,j} + CL_{benefit,i,j} + YD_{benefit,i,j} \quad (14)$$

where ecosystem health benefits ($EH_{benefit,i,j}$) refer to the avoided damage cost of negative N_r effect on ecosystems ($/kg N). Several studies have already examined the unit damage cost on ecosystems for the EU and USA using the willingness to pay (WTP) method [70-77]. However, such cost and benefit data are not directly available for China. Therefore, we

adopted a new set of unit N_r damage costs to ecosystems (Table S8) across various regions (at the provincial level) in China. By utilizing the regional-specific WTP and PGDP as proxies for adjustment, we evaluated the benefits and trade-offs of nitrogen related mitigation measures in different regions, as indicated by Equation (15):

$$EH_{benefit,i} = \sum_j \Delta E_{i,j} \times \partial_{US,j} \times WTP_i/WTP_{US} \times PGDP_i/PGDP_{US} \times \mu e_{i,j} \quad (15)$$

where $\Delta E_{i,j}$ represents the change of specific N_r loss in form j; $\partial_{US,j}$ is the the estimated unit ecosystem damage cost of N_r loss in USA ($/kg N, values are shown in Table S8); WTP_i and WTP_{US} refer to the values of the WTP for ecosystem service in county *i* and the USA ($), respectively; $PGDP_i$ and $PGDP_{US}$ are the per capita gross domestic product (PGDP, in constant 2020 USD) of county *i* and the USA, respectively. The welfare implications of damage transformation are predicated on WTP, with the provincial-level WTP data for ecosystem services in China sourced from Xu et. al, 2025 [78]. The adjusted ecosystem damage costs for each province are shown in Table S9. Additionally, based on the exceedance level for corresponding boundary in county *i*, we established a marginal benefit adjustment coefficient $\mu e_{i,j}$ to better highlight the damage to the regional environment by varying degrees of N_r pollution (Table S10).

The human health benefit ($HH_{benefit,i,j}$) quantifies the prevented mortality resulting from the mitigation of $PM_{2.5}$ attributable to N_r gas reduction [13]. Provincial-specific unit costs for human health damage from atmospheric release of N_r were estimated using the methodology developed by Gu et al. 2021, which correlates the economic cost of mortality per unit of atmospheric release of N_r with factors including population density, PGDP, urbanization rate, and nitrogen-share. The calculation of health benefits derived from N_r gas mitigation is expressed in Equation (16):

$$HH_{benefit,i,j} = \sum_j \Delta E_{i,j} \times HCost_{i,j} \times \mu h_{i,j} \quad (16)$$

where $\Delta E_{i,j}$ is the reduction in specific N_r loss; $HCost_{i,j}$ represents the unit health damage cost of atmospheric release of N_r ($/kg N), with original parameters shown in Table S8, and province-specific adjusted values in Table S9. Similarly, $\mu h_{i,j}$ is the marginal benefit adjustment coefficient for human health (Table S10).

For monetary evaluation of the climate impact ($CL_{benefit,i,j}$), regional-weighted N_r damage cost was used for calculation, as formulated in Equation (17):

$$CL_{benefit,i,j} = \sum_j \Delta E_{i,j} \times CCost_{i,j} \quad (17)$$

where $CCost_{i,j}$ represents the unit abatement cost to the climate ($/kg N), with specific parameters shown in Table S8. It is important to note that, since the focus of this study is not on N_2O, we did not include N_2O in the climate impact calculations. Instead, we specifically concentrated on the cooling effects of NO_x and NH_3 emissions on the global climate [36, 79].

The yield benefit ($YD_{benefit,i}$) comprises the additional economic benefits derived from increased crop or animal yield, as expressed in Equation (18):

$$YD_{benefit,i} = \Delta Hv_i \times YP_i \quad (18)$$

where ΔHv_i refers to the change in crop yield or livestock production (kg), calculated using the same methodology as $\Delta E_{i,j}$ in Equation (8); YP_i is the integrated crop or livestock price ($ kg^{-1}) in USD on the basis of the Compilation of National Agricultural Product Cost and Benefit Data [80], Gu et al. 2023 [81], and Khan et al., 2019 [82].

S2. Description of CHANS model

The Coupled Human And Natural Systems (CHANS) model was employed to calculate the nitrogen budget for 2,847 counties across China. CHANS is a process-based nitrogen-flow model that focuses on the nitrogen cycle at the interface between human and natural systems. This model encompasses virtually all biogeochemical processes associated with the nitrogen cycle. The CHANS-China model integrates the fluxes of nitrogen inputs, output, and accumulations among 15 subsystems, utilizing a mass balance approach. In terms of spatial coverage, the model encompasses approximately 9.6 million square kilometers of mainland China, excluding Hong Kong, Macau, and Taiwan due to data limitations. Vertically, the upper boundary is defined at about 1 km above ground level, while the lower boundary extends to encompass all materials above the bedrock, including soil and groundwater. The nitrogen cycle initiates with the entry of N_r from N_2 or through the mobilization of fossil fuels and terminates with its reduction to N_2 or losses to systems beyond China's borders.

Gu et al. [83] validated the model's calculated nitrogen fluxes by employing atmospheric quality monitoring data from ground observation networks, water quality data, and NH_3 and NO_x column concentrations obtained from remote sensing satellites [84, 85]. Results indicate that the atmospheric release of N_r calculated by CHANS closely aligns with measured values, achieving an R^2 value exceeding 0.7. Conversely, nitrogen fluxes in aquatic environments demonstrated moderate agreement with an R^2 value of approximately 0.5 [83], primarily attributed to the complex nature of nitrogen cycling processes in aquatic ecosystems (e.g., denitrification occurring in hyporheic zones). Consequently, it can be concluded that the model's calculated nitrogen fluxes are fundamentally accurate and can be employed to evaluate N_r loads to both air and water.

Based on Gu et al. [86], we categorized the 15 subsystems within CHANS into 5 sectors and 3 environmental receptors (Fig. S12). Specific parameters and sources are outlined in Table S11. Here are detailed definitions for each sector and receptor:

S2.1 The cropland sector

The cropland (CL) subsystem is the sole subsystem within the cropland sector. This subsystem primarily encompasses terrestrial ecosystems that cultivate grains, vegetables, fibers, sugar crops, fruits, and other economic crops artificially. Nitrogen inputs to the cropland subsystem primarily include fertilizers, irrigation, manure, biological nitrogen fixation (BNF), nitrogen deposition, and recycled straw, which can be calculated using the following formula:

$$N_{input,CL} = N_{fer,CL} + N_{irr,CL} + N_{exc,CL} + N_{fix,CL} + N_{dep,CL} + N_{str,CL} \quad (1)$$

where $N_{input,CL}$ represents the total nitrogen input within cropland, $N_{fer,CL}$ denotes the nitrogen content in synthetic fertilizers; $N_{irr,CL}$ indicates the nitrogen input from irrigation water; $N_{exc,CL}$ refers to the excretion (including animal manure and human excretion) recycled to the cropland; $N_{fix,CL}$ signifies biological nitrogen fixation (BNF), including both symbiotic and non-symbiotic processes; $N_{dep,CL}$ represents the total amount of atmospheric dry and wet nitrogen deposition on cropland; $N_{str,CL}$ indicates the nitrogen contained in straw being recycled to cropland. Nitrogen outputs from the cropland subsystem primarily comprise grain and straw harvested, nitrogen loss to air and water, which can be calculated using the following equation:

$$N_{output,CL} = N_{crop,CL} + N_{str,CL} + N_{gas,CL} + N_{leach,CL} + N_{runoff,CL} \quad (2)$$

where $N_{output,CL}$ denotes the total nitrogen output from the cropland sector; $N_{crop,CL}$ indicates the nitrogen content in harvested crops, including grain consumed by humans or

utilized as aquaculture and livestock feed; $N_{str,CL}$ refers to the nitrogen removal via harvested straw, including straw used as livestock feed, biomass fuel and industrial materials; $N_{gas,CL}$ encompasses nitrogen gases (including NH_3, N_2O, NO_x, and N_2) emitted during crop production and straw burning; $N_{leach,CL}$ and $N_{runoff,CL}$ signifies NO_3^- leaching to groundwater and runoff to river.

S2.2 The livestock sector

Similarly, The livestock (LS) subsystem is the sole subsystem within livestock sector. This study categorizes total livestock production into two categories: grazing and husbandry, to differentiate between the contributions of grasslands and domesticated livestock. All domesticated animals are incorporated into the livestock subsystem, whereas remaining grazing animals are classified under the grassland system. Nitrogen inputs to the livestock subsystem primarily consist of animal feed, which can be calculated using the following formula:

$$N_{input,LS} = N_{feed,LS} + N_{fer,LS} \tag{3}$$

where $N_{input,LS}$ represents the total nitrogen input within the livestock subsystem; $N_{feed,LS}$ denotes the nitrogen content in animal feed, specifically including domestically produced grains and straw, forage, and fish powder; $N_{fer,LS}$ refers to the nitrogen input used for straw ammonization to produce feed. Nitrogen outputs from the livestock subsystem primarily consist of animal products, manure excretion, nitrogen gas emissions, and nitrogen loss to water. This can be represented by the following equation:

$$N_{output,LS} = N_{ani,LS} + N_{man,LS} + N_{gas,LS} + N_{water,LS} \tag{4}$$

where $N_{output,LS}$ indicates total nitrogen output from the livestock sector; $N_{ani,LS}$ is nitrogen content in animal products designated for human consumption or industrial purposes; $N_{man,LS}$ signifies nitrogen content in livestock manure; $N_{gas,LS}$ encompasses nitrogen gas emissions during housing and storage (primarily NH_3, N_2O), while $N_{water,LS}$ signifies livestock wastewater, which is either transferred to wastewater treatment plants (WTPs) or directly discharged into water bodies..

S2.3 The human sector

It encompasses all nitrogen cycling processes associated with human food and energy consumption, as well as waste disposal, thereby closely linking it to other subsystems. The human sector comprises a total of six subsystems, including the aquaculture subsystem (AQ), urban green-land subsystem (UG), human subsystem (HM), pet subsystem (PT), garbage subsystem (GB) and wastewater subsystem (WW).

China is the world's largest aquaculture producer, and the aquaculture subsystem (AQ) encompasses both natural and cultivated aquatic products, such as fish, shrimp and crabs in both freshwater and seawater. Given that natural fishery production contributes to animal protein for both humans and livestock, it is also regarded as an input for the aquaculture subsystem [86]. Nitrogen inputs consist of fish feed, nitrogen content from cultivated fisheries, fertilizers and nitrogen deposition, which can be calculated using the following formula:

$$N_{input,AQ} = N_{feed,AQ} + N_{fishery,AQ} + N_{fer,AQ} + N_{dep,AQ} \tag{5}$$

where $N_{input,AQ}$ represents the total nitrogen input for the aquaculture subsystem; $N_{feed,AQ}$ denotes nitrogen contained in aquaculture bait; $N_{fishery,AQ}$ refers to the nitrogen content in natural fishing; $N_{fer,AQ}$ indicates the nitrogen from fertilizers required for the production process of fish feed; $N_{dep,AQ}$ signifies atmospheric nitrogen deposition in the aquaculture area. Nitrogen outputs from the aquaculture subsystem primarily comprise harvested aquatic

products and N_r losses, which can be expressed by the following equation:

$$N_{output,AQ} = N_{meat,AQ} + \sum_{i=1}^{4} N_{loss,AQ,i} \quad (6)$$

where $N_{output,AQ}$ indicates the total nitrogen output from the aquaculture subsystem; $N_{meat,AQ}$ represents nitrogen contained in aquaculture production (including fish, shrimp, shellfish, algae, and others) used for human food or livestock feed. Additionally, $N_{loss,AQ}$ refers to nitrogen loss losses during aquaculture processes, primarily including NH_3, N_2O, NO_3^-, and N_2 emissions.

The urban green-land subsystem (UG) pertains to urban land covered with natural or artificial vegetation, including urban lawns and shrubs. The maintenance of lawns plays a crucial role in nitrogen inputs, which derive from fertilizers, pet waste, biological nitrogen fixation, and nitrogen deposition. Nitrogen inputs for the urban green-land subsystem can be calculated using the following formula:

$$N_{input,UG} = N_{fer,UG} + N_{man,UG} + N_{fix,UG} + N_{dep,UG} \quad (7)$$

in this equation, $N_{input,UG}$ represents the total nitrogen input to the urban green-land subsystem; $N_{fer,UG}$ denote the nitrogen content in chemical fertilizers applied to lawns; $N_{man,UG}$ refer to the nitrogen contained in pet manure recycled to urban lawns; $N_{fix,UG}$ indicates the amount of biological nitrogen fixation in urban lawns; $N_{dep,UG}$ signifies atmospheric nitrogen deposition. And the nitrogen outputs from the urban green-land subsystem primarily comprise green waste and nitrogen losses, which can be expressed by the following equation:

$$N_{output,UG} = N_{was,UG} + \sum_{i=1}^{6} N_{loss,UG} \quad (8)$$

Here, $N_{output,UG}$ indicates total nitrogen output from the urban green-land subsystem; $N_{was,UG}$ accounts for nitrogen removal through pruning branches or mowing of lawns, with this green waste typically sent to landfill or subjected to burning, while some is recycled within the urban green-land subsystem; $N_{loss,UG}$ refers to nitrogen losses, including NH_3, NO_x, N_2O and N_2 produced during denitrification, as well as NO_3^- runoff and leakage.

The human subsystem (HM) encompasses processes related to food and energy consumption. Nitrogen inputs to the human system primarily consist of three components: food, daily necessities, and fuels, which can be described using the following formula:

$$N_{input,HM} = N_{food,HM} + N_{indu,HM} + N_{fuel,HM} \quad (9)$$

where $N_{input,HM}$ represents the total nitrogen input to the human subsystem; $N_{food,HM}$ denotes the nitrogen content in human food consumption, which includes grains from the cropland subsystem, livestock products (meat, eggs, and dairy) from the livestock subsystem, and aquatic products from the aquaculture subsystem; $N_{indu,HM}$ refer to the nitrogen inputs from the consumption of necessities (primarily nitrogen-containing industrial products from the industrial subsystem) by humans; $N_{fuel,HM}$ indicates the nitrogen input from domestic fuel consumption encompassing fossil fuels, straw, firewood, and manure. The nitrogen outputs from the human system mainly consist of three components: human metabolism, solid waste, and fuel combustion, which can be expressed by the following equation:

$$N_{output,HM} = N_{exc,HM} + N_{gar,HM} + N_{gas,HM} \quad (10)$$

here, $N_{output,HM}$ indicates the total nitrogen output from the human subsystem; $N_{exc,HM}$ refers to the nitrogen content in human excreta that is either recycled to cropland, transferred to wastewater treatment plants (WTPs), discharged directly into surface water, or leached into groundwater.; $N_{gar,HM}$ represents the nitrogen output from discarded food and industrial products, where the food waste primarily includes waste from household, restaurants and

retail; it is assumed that 50% of structural industrial nitrogen products (e.g., furniture, plastics, paper, rubber, and fibers) are sent to landfills while all non-structural industrial nitrogen products (e.g., explosives) and non-food waste are directly discarded into the environment after consumption; and $N_{gas,HM}$ denotes the nitrogen gas emissions during household energy combustion and human activities (such as respiration, sweating, and flatulence), primarily including NH_3, N_2O, NO_x, and N_2.

The pet subsystem (PT) functions as an affiliated subsystem of the human subsystem. This study exclusively focuses on dogs and cats, excluding other pets due to smaller populations. Nitrogen inputs for the pet system mainly consist of pet food, while outputs are primarily derived from pet excrement. The calculations can be expressed as follows:

$$N_{input,PT} = N_{feed,PT} \quad (11)$$

$$N_{output,PT} = N_{exc,PT} \quad (12)$$

where $N_{input,PT}$ and $N_{output,PT}$ are the total nitrogen input and output within the pet subsystem, respectively; $N_{feed,PT}$ denote the nitrogen contained in pet feed, specifically dog and cat food; $N_{exc,PT}$ account for nitrogen contained in pet excretions, part of which is deposited in urban lawns while the remainder is treated as waste and sent to landfills for centralized treatment.

The garbage subsystem (GB) functions as a nitrogen removal system that accepts and processes solid waste generated by human production and consumption activities, thereby mitigating. Nitrogen inputs for the garbage subsystem include all waste transported to garbage treatment facilities:

$$N_{input,GB} = N_{gre,GB} + N_{dom,GB} + N_{pet,GB} + N_{slud,GB} \quad (13)$$

where $N_{input,GB}$ indicates the total nitrogen input to the garbage subsystem; $N_{gre,GB}$ refers to the nitrogen content in landscaping waste transported from urban lawns to landfills; $N_{dom,GB}$ denotes the nitrogen input from solid waste generated by human activities; $N_{pet,GB}$ signifies the nitrogen content in pet feces entering landfills; and $N_{slud,GB}$ represents the nitrogen content in sludge generated during domestic sewage and industrial wastewater treatment processes. Nitrogen outputs from the garbage system are categorized into four disposal pathways: landfill, incineration, composting, and discarding (primarily rural solid waste), while other outputs are considered accumulations within the subsystem. This can be expressed as follows:

$$N_{output,GB} = N_{gas,GB} + N_{comp,GB} + N_{leach,GB} \quad (14)$$

here, $N_{output,GB}$ indicates the total nitrogen output from the garbage subsystem; $N_{gas,GB}$ encompasses nitrogen gas emissions during various garbage treatment processes (primarily NH_3, N_2O, NO_x, and N_2). $N_{comp,GB}$ denotes total nitrogen from composted household waste, and $N_{leach,GB}$ represents the nitrogen content in leachate produced during landfilling and discarding.

The wastewater subsystem (WW) functions as another nitrogen removal system that address liquid waste generated during human production and consumption activities to mitigate environmental impacts. Nitrogen inputs to the wastewater subsystem encompass all wastewater transferred to treatment plants. The calculation can be expressed as follows:

$$N_{input,WW} = N_{dom,WW} + N_{ind,WW} + N_{ani,WW} + N_{leachate,WW} \quad (15)$$

where $N_{input,WW}$ indicates the total nitrogen input to the wastewater subsystem; $N_{dom,WW}$ denotes the nitrogen content in domestic sewage; $N_{ind,WW}$ refers to nitrogen content in industrial effluents; $N_{ani,WW}$ denoted the nitrogen contained in livestock sewage; and

$N_{leachate,WW}$ signifies nitrogen entering wastewater treatment plants from landfill-generated leachate. The outputs of the wastewater subsystem comprise five components: effluent discharge, recycled wastewater, sludge treatment, gas emissions, and leachate. This can be expressed as follows:

$$N_{output,WW} = N_{river,WW} + N_{rec,WW} + N_{sluge,WW} + N_{gas,WW} + N_{leach,WW} \quad (16)$$

here, $N_{output,WW}$ indicates total nitrogen output from the wastewater subsystem; $N_{river,WW}$ signify the nitrogen contained in treated effluent discharged into rivers; $N_{rec,WW}$ refers to the nitrogen content in recycled water after treatment; $N_{sluge,WW}$ denotes the total nitrogen contained in sludge produced during wastewater processing; $N_{gas,WW}$ encompasses nitrogen gas emission (including N_2O, NH_3, and N_2) during the treatment process; and $N_{leach,WW}$ refers to N_r leaching of wastewater during sewage collection.

S2.4 The industry sector

This sector mainly encompasses the industry subsystem (ID) and its affiliated subsystem—the traffic subsystem (TF).

The industry subsystem (ID) is defined in this study as a manufacturing sector that functions as a production end providing industrial products such as cement, lime, and fertilizers. This subsystem primarily operates as a processing facility, characterized by rapid material flow. Nitrogen inputs to the industry subsystem primarily consist of industrial raw materials, fossil fuel energy, and nitrogen fixed from N_2 through the Haber-Bosch process (HBNF), as represented by the following equation:

$$N_{input,ID} = N_{agr,ID} + N_{ani,ID} + N_{timber,ID} + N_{fuel,ID} + N_{HBNF,ID} \quad (17)$$

where $N_{input,ID}$ is the total nitrogen input to industry subsystem; $N_{agr,ID}$ denote the nitrogen content in agricultural products entering industrial food processing—encompassing crops such as cotton, tobacco, hemp—and industrial grains such as liquor, beer, and corn starch; $N_{ani,ID}$ refers to the nitrogen content in animal products sourced from livestock subsystem, such as silk, leather, feather, and wool; $N_{timber,ID}$ indicates the nitrogen content in timber processed for manufacturing furniture or other products that sourced from the forest subsystem; $N_{fuel,ID}$ signifies the nitrogen content in fossil fuels consumed during energy production and processing; $N_{HBNF,ID}$ denotes the amount of N_2 fixed to synthesize NH_3 using HBNF. Nitrogen outputs from this system primarily include industrial products, industrial emissions, wastewater, and other waste materials. This can be expressed as follows:

$$N_{output,ID} = N_{prod,ID} + N_{gas,ID} + N_{waste,ID} \quad (18)$$

here, $N_{output,ID}$ indicates the total nitrogen output from the industry subsystem; $N_{prod,ID}$ represents the nitrogen contained in industrial products such as fertilizers, plastics, coatings, synthetic fibers, synthetic rubber, dyes, resins, drugs, dynamite, chemical reagents, pesticides, detergents, and nitrates. This study categorizes nitrogen-containing consumer goods produced by the industrial system into two types: non-structural nitrogen products, which are artificially synthesized through ammonia pathways; and structural nitrogen products, which are derived from biological nitrogen-containing raw materials sourced from agriculture or forestry. $N_{gas,ID}$ refers to nitrogen gases emitted during industrial production processes (primarily including NH_3, N_2O, NO_x, and N_2; with N_2O primarily originating from adipic acid and nitric acid production) [87]; $N_{waste,ID}$ represents nitrogen output contained in industrial waste discharge (including wastewater and solid waste), where a portion of the wastewater is treated at sewage treatment facilities while the remainder is directly discharged directly into surface water.

The traffic subsystem (TF) is an important energy consumer that utilizes substantial amounts of fossil fuels during transportation processes and emits considerable quantities of NO_x and NH_3. Due to data limitations, this study exclusively focuses on road traffic as a primary source of N_r emissions, classifying it based on different vehicle types and transport categories (passenger and freight). The nitrogen inputs and outputs for this system can be calculated using the following equations:

$$N_{input,TF} = N_{fuel,TF} + N_{N_2,TF} \quad (19)$$

$$N_{output,TF} = N_{gas,TF} \quad (20)$$

where $N_{input,TF}$ and $N_{output,TF}$ are the total nitrogen input and output for the traffic subsystem, respectively; $N_{fuel,TF}$ denotes the nitrogen content in fossil fuels used for transportation heat or power. In this study, we assumed that the primary energy sources utilized are coal and petroleum, while excluding electricity from consideration. $N_{N_2,TF}$ signifies the N_2 that is converted into N_r during transportation; and $N_{gas,TF}$ refers to nitrogen gases produced during transportation processes (primarily including NO_x and NH_3).

S2.5 The nature sector

It mainly includes areas that are less disturbed by human activities and comprises two subsystems: grassland and forest.

Grasslands constitute the most extensively distributed terrestrial ecosystem in China. In this study, permanent grasslands (including both natural and artificial grasslands) utilized for grazing or forage harvesting for livestock feeding are categorized within the grassland subsystem (GL). Additionally, activities associated with grazing animals and their in-situ excretion are incorporated into the grassland subsystem owing to their intrinsic connection to grassland ecosystems. We assumed that all manure produced by grazing livestock remain on the grasslands. The nitrogen inputs for the grassland subsystem can be calculated using the following equation:

$$N_{input,GL} = N_{fer,GL} + N_{fix,GL} + N_{dep,GL} + N_{irr,GL} \quad (21)$$

where $N_{input,GL}$ is the total nitrogen input to the grassland system; $N_{fer,GL}$ pertain to the nitrogen from chemical fertilizers applied to grasslands (primarily on artificial grasslands); $N_{fix,GL}$ denotes biological nitrogen fixation occurring during grass growth; $N_{dep,GL}$ signifies atmospheric nitrogen deposition onto the grassland area; $N_{irr,GL}$ indicates nitrogen content in irrigation water utilized for artificial grasslands. The nitrogen outputs from the grassland subsystem primarily include livestock products, forage harvests, manure, gaseous emissions, leaching, and runoff:

$$N_{output,GL} = N_{gra,GL} + N_{for,GL} + N_{gas,GL} + N_{man,GL} + N_{leach,GL} + N_{runoff,GL} \quad (22)$$

here, $N_{output,GL}$ represents the total nitrogen output from the grassland subsystem; $N_{gra,GL}$ denotes the nitrogen in livestock products derived from grazing animals, primarily consumed by humans or utilized as industrial raw materials. $N_{for,GL}$ refers to nitrogen harvested from forage crops; $N_{man,GL}$ indicates the nitrogen in manure produced by grazing livestock; $N_{gas,GL}$ encompasses nitrogen gas emissions (primarily including NH_3, N_2O, NO_x, and N_2) resulting from processes such as fertilization, denitrification, fire-biomass combustion and other biochemical processes; $N_{leach,GL}$ and $N_{runoff,GL}$ represent NO_3^- losses to surface water and groundwater originating from the grasslands.

The forests in China are primarily categorized into six types: evergreen broadleaf forests, mixed coniferous-broadleaf forests, deciduous broadleaf forests, coniferous forests, bamboo, and bushes. Urban woodlands are excluded from this classification as they are categorized

under urban green spaces. The nitrogen inputs to the forest subsystem (FR) generally consist of fertilizers, biological nitrogen fixation, and nitrogen deposition:

$$N_{input,FR} = N_{fer,FR} + N_{fix,FR} + N_{dep,FR} \quad (23)$$

where $N_{input,FR}$ denotes the total nitrogen inputs to the forest subsystem; $N_{fer,FR}$ represent the nitrogen content in fertilizers applied to afforestation efforts aimed at enhancing soil fertility and promoting tree growth; $N_{fix,FR}$ refers to nitrogen fixed during growth of living wood; $N_{dep,FR}$ accounts for both dry and wet nitrogen deposition onto forest ecosystems. Nitrogen outputs from the forest system include timber production and denitrification:

$$N_{output,FR} = N_{product,FR} + N_{gas,\,FR} + N_{leach,FR} + N_{runoff,FR} \quad (24)$$

here, $N_{output,FR}$ indicates the total nitrogen output from the forest subsystem; $N_{product,FR}$ includes the nitrogen content in all timber utilized as firewood or industrial raw materials; $N_{gas,\,FR}$ refers to nitrogen gases emitted during biomass combustion and denitrification processes (primarily including NH_3, N_2O, NO_x, and N_2). Additionally, $N_{leach,FR}$ and $N_{runoff,FR}$ signify NO_3^- loss to groundwater and surface water originating from forested areas.

S2.6 Near-surface atmosphere

The atmospheric system (AT) encompasses the near-surface atmosphere within a specified region. All gases released from other subsystems are regarded as nitrogen inputs to this subsystem. N_r in the near-surface atmosphere can be transported to other systems via atmospheric circulation and subsequently redeposited onto terrestrial surfaces. The nitrogen flow within the atmospheric system can be expressed by the following equations:

$$N_{input,\,AT} = \sum_{i=1}^{13} N_{item,AT,i} \quad (25)$$

$$N_{output,\,AT} = N_{dep,AT} + N_{exp,AT} \quad (26)$$

where $N_{input,AT}$ and $N_{output,AT}$ represent the total nitrogen inputs and outputs within the atmospheric subsystem, respectively; $N_{item,AT}$ indicates the total nitrogen content resulting from the atmospheric release of nitrogen gases (including NH_3, NO_x, N_2O, and N_2) originating from 13 distinct subsystems: cropland, livestock, aquaculture, grasslands, forests, urban green-land, human, pets, garbage, wastewater, industry, traffic, and surface water. Furthermore, $N_{dep,AT}$ refers to atmospheric nitrogen deposition encompassing both dry and wet deposition primarily in the form of ammonium and nitrate [88], while $N_{exp,AT}$ signifies the total amount of nitrogen gas transported beyond the region to surrounding areas (e.g., adjacent coastal marine environments) via atmospheric circulation.

S2.7 Surface water

The surface water subsystem (SW) includes all rivers, lakes, and wetlands that receive inflows from various sources. It receives nitrogen inputs from other subsystems and transfers them to the ocean or neighboring countries. The equations utilized for calculations are as follows:

$$N_{input,SW} = \sum_{i=1}^{8} N_{item,SW,i} + N_{dep,SW} \quad (27)$$

$$N_{output,SW} = N_{irr,SW} + N_{gas,WS} + N_{ocean,SW} \quad (28)$$

where $N_{input,SW}$ and $N_{output,SW}$ are the total nitrogen inputs and outpust within the surface water subsystem, respectively; $N_{item,SW}$ represents nitrogen runoff from other subsystems, including cropland, livestock, aquaculture, forest, grassland, urban green-land, human, industry and effluent from wastewater treatment plants. Additionally, $N_{dep,SW}$ signifies N_r that directly deposits onto the surface water. $N_{irr,SW}$ refers to the nitrogen content in the irrigation water utilized in agricultural fields; $N_{gas,WS}$ represents the nitrogen released as N_2

and N_2O during denitrification processes in surface water, with this release accounting for approximately 40% to 50% of total nitrogen outputs [89, 90]; and $N_{ocean,SW}$ denotes nitrogen exported to the ocean from rivers.

S2.8 Groundwater

Groundwater refers to various forms of water located within the voids of rock or soil layers beneath the Earth's surface. The groundwater subsystem (GW) predominantly receives leachate from other subsystems. The nitrogen budget within this system can be calculated using the following equations:

$$N_{input,GW} = \sum_{i=1}^{8} N_{item,GW,i} \quad (29)$$

$$N_{output,GW} = N_{irr,GW} \quad (30)$$

where $N_{input,GW}$ and $N_{output,GW}$ represent the total nitrogen input and output for the groundwater subsystem, respectively; $N_{item,GW}$ denotes the amount of N_r that leaches into groundwater from other subsystem (including cropland, livestock, forest, grassland, domestic sewage leaks, urban green-land, and waste landfill); $N_{irr,GW}$ refers to nitrogen contained in groundwater extracted for irrigation purposes. Any residual nitrogen tends to accumulate over time in the groundwater subsystem, resulting in elevated nitrate concentrations in groundwater.

S3. Supplementary Discussion

S3.1 Uncertainty analysis

This study conducted an uncertainty analysis of the nitrogen budget in 2020 and under CSM within all subsystems in the CHANS model using the Monte Carlo simulation method. Monte Carlo simulation is a mathematical technique that generates a set of random numbers according to the data distribution and parameters for each variable. In this study, we integrated Monte Carlo simulation into the CHANS model to estimate uncertainties of various nitrogen budgets. Theoretically speaking, there are three dominant sources of uncertainties in nitrogen budget calculations: First, the variation in input data (mainly including statistical and measured data) implies a measure of uncertainty, fine data on the spatial distribution of nitrogen inputs and losses shows a certain degree of scarcity. Second, the process of combining data from various sources and meta-analysis inevitably simplified the complex biogeochemical and hydrological nitrogen cycling and left out spatial heterogeneity, which may lead to biases and uncertainties. Third, the imperfections in model structure, interactions between different parameters and processes, as well as the reliability and applicability of the parameters or coefficients in CHANS model may all contribute to uncertainties. The specific steps of uncertainty assessment can be referred to in Zhang et al. 2021 [91]. Generally, activity data collected from official statistics or direct measurements exhibit a lower coefficient of variation (CV, 5%-10%); other activity data and parameters derived from published studies exhibit a higher CV (20%-50%); and emission factors for different nitrogen fluxes exhibit the highest CV (50%-100% or even higher) due to variations caused by anthropogenic and natural factors across China. All coefficients of variations (CV) for activity data and main parameters are primarily summarized from published literature, as shown in Table S11. To improve the accuracy of the results, this study performed 10,000 iterations of Monte Carlo simulations. By conducting the above processes, uncertainties for nitrogen losses from each subsystem in the CHANS model and their contributions to total uncertainties in 2020 were identified (Table S12).

Apart from the calculation of nitrogen budget, the uncertainty of the nitrogen losses to air and water under CSM is predominantly influenced by the mitigation efficiency of each mitigation

measure. It can be seen from the result of meta-analysis and literature review that nearly all the mitigation options come with an uncertainty range due to the variations in specific local conditions such as soil, climate, equipment and management practice. In this study, the uncertainty ranges of mitigation options for each sector are shown in Figs. S6-S7 and Table S5 alongside the mean values. The cropland and livestock sectors contribute a substantial portion of the uncertainties among all sectors since the effectiveness of management practices in these two sectors are most sensitive to local conditions.

What's more, when formulating safe nitrogen boundaries based on the sensitivity and impact of receiving environments, many factors may result in uncertainties:

(1) Ecosystem-specific N_r sensitivity: This study's broad classification of vegetation into 12 categories fails to capture the nuanced variations in nitrogen deposition tolerance among sub-categories. We used the area-weighted average critical loads of different vegetation types with varying sensitivities rather than the most sensitive vegetation type to calculate the critical nitrogen deposition loads within certain regions. Besides, the adoption of a uniform threshold value of 1.0 mg N L^{-1} for TN (Total Nitrogen) in surface oversimplifies the diverse range of critical nitrogen concentrations (from 0.5 to 4.0 mg N L^{-1}) necessary to prevent eutrophication across various aquatic ecosystems (e.g., lakes, rivers, coastal areas) [92-94].

(2) Multi-factorial ecological impacts: The ecological status of air and water is influenced by various nutrient substances and environmental factors beyond nitrogen alone. For instance, in addition to nitrogen, phosphorus is also an important nutrient loading that leads to cyanobacterial bloom formation [95, 96]. Usually, the imbalance of stoichiometry for nitrogen & phosphorus leads to the eutrophication of a water body [97]. Although the selected mitigation measures can potentially reduce phosphorus loss to water as well, their mitigation potential remains unquantified, potentially leading to unforeseen ecological consequences.

(3) Temporal dynamics and legacy effects: Since this study didn't account for time-dependencies, we ignored groundwater nitrogen legacy and its delivery to surface water. Extensive research has demonstrated that excessive nitrogen accumulation in groundwater creates substantial nitrogen reservoirs [98-100]. Part of the legacy nitrogen will be delivered from groundwater to surface water through lateral flow, which leads to time lags in water quality improvement. Accurate quantification of nitrogen loading in surface and groundwater systems necessitates the development of long-term, time-series hydrologic models that account for these legacy effects.

(4) Complexity in N_r trajectory estimation: Tracing the path of N_r through air and water systems presents significant challenges. Atmospheric N_r dynamics are subject to uncertainties arising from complex, multifactorial processes spanning precursor release, gas-particle transformations, and ultimate deposition. In aquatic systems, uncertainties in hydrological parameters and local conditions (e.g., soil texture, aquifer porosity) complicate the estimation of nitrate concentrations, particularly in groundwater systems.

In this study, we employed the atmospheric and hydrological conditions from 1950 to 2022 to calculate the uncertainty associated with the safe nitrogen boundaries (Fig. 1a). However, it is important to note that given the more pronounced climate changes observed in China since the early 2000s, the calculated safe nitrogen boundary values would also shift accordingly. Notably, compared with the safe boundary for nitrogen runoff of 5.2 Tg N yr^{-1} derived using the long-term mean hydrological conditions from 1950 to 2022, applying the more recent average conditions from 2000 to 2022 yields a substantially lower boundary of 4.5 Tg N yr^{-1}. This reduction reflects decreased precipitation as well as the increasing frequency of hydrological extremes; for instance, runoff in 2011 was 36% lower than the long-term mean in China. These tighter limits highlight the intensifying environmental pressures and shrinking

thresholds under contemporary climate conditions, suggesting that our study may underestimate the risk of surface-water nitrogen exceedance under ongoing climate change. Looking ahead, dynamically adjusting boundary estimates using real-time annual climate data will be essential for developing adaptive and climate-responsive safe nitrogen boundaries.

S3.2 Limitations

This study innovatively developed targeted mitigation strategies based on the exceedance levels of regional nitrogen boundaries, providing a practical benchmark for defining a "safe operating space" for human activities. However, the results of this study still have imperfections due to the following limitations:

Firstly, we integrated the effectiveness of various N_r mitigation measures derived from a nationwide meta-analysis with the CHANS model to conduct the mitigation potential in the cropland and livestock sector. Meta-analysis is a robust methodology for synthesizing results and promoting the precision of the conclusions. Despite collecting over 4,000 observations, limited experiments were conducted in regions such as Tibet, Qinghai, and Xinjiang provinces. Consequently, the efficacy of several management practices may not have been adequately evaluated in these areas. Moreover, variations in social contexts and local policies across provinces may influence the actual mitigation potential, despite our consideration of the urgency of N_r reductions and regional economic affordability. Given these limitations, it is evident that our approach inevitably incorporates simplifications that may not fully capture the complexity of real-world scenarios.

Secondly, this study creatively bridged the gaps between the economic opportunities of N_r management practices and their cost-effectiveness concerning human health and ecosystem service through cost-benefit analysis (CBA). Nevertheless, such an innovative approach has its inevitable limitations. The accurate evaluation of implementation costs for N_r mitigation measures is complicated by various uncontrollable and ancillary factors. For instance, our cost calculations for mitigation measures in the cropland sector only accounted for labor wages and direct input costs (e.g., machinery and fertilizer expenses). However, subsidy costs, opportunity costs, and educational expenses, which are crucial for the feasibility of N_r management practices, were not fully incorporated into our calculations. And for the monetization of societal benefits, unlike previous research [64, 101-103], this study has established the marginal benefit adjustment coefficients based on boundary exceedance levels to better reflect the social costs induced by N_r pollution. However, this approach inherently differs from direct economic income quantification, potentially introducing limitations to the valuation process. Moreover, unlike the global-scale climate effects of carbon emissions, the environmental impacts induced by N_r can be localized. Quantifying the implementation costs and social benefits of N_r emission reduction at a relatively small scale is challenging, necessitating a comprehensive assessment of regional climatic, social, and economic factors. This doesn't imply that the regional-scale assessments conducted in this study lack significance. Instead, it is crucial to transparently acknowledge these limitations and, as much as possible, quantify the resulting uncertainties or provide qualitative caveats.

Third, the CSM scenario is designed to represent an upper-bound estimate of the currently achievable technical mitigation potential. To account for mitigation measures that have already been implemented in practice, model parameters in CHANS were adjusted rather than relying solely on historical practices or reported adoption rates, which remain incomplete due to data constraints. In contrast, when estimating additional reduction potential, traditional practices or outdated equipment without mitigation were treated as the baseline, which may

lead to an overestimation of incremental mitigation under CSM. Moreover, although the selected mitigation measures were compiled from an extensive review of the available literature, they may not exhaustively capture all technically feasible options, and their effective implementation often depends on enabling socioeconomic transformations, such as farm consolidation, recoupling of cropland and livestock systems, and shifts in consumption patterns, which provide the structural conditions required for broader adoption. In addition, several mitigation measures often affect different subsystems sequentially, and their combined effects are not strictly additive. These simplifications, together with potential mismatches between modeled baselines and real-world implementation, imply that the estimated mitigation potential under CSM should be interpreted as an optimistic benchmark. Importantly, the finding that even such an upper-bound scenario remains insufficient to fully resolve water-related nitrogen pollution underscores the structural difficulty of controlling aqueous nitrogen losses and highlights the need for complementary institutional and socioeconomic transformations beyond technical measures alone.

Furthermore, this study acknowledges the existence of cultural and regional heterogeneities that are challenging to fully incorporate into the analytical framework. For example, the relative significance attributed to various environmental threats exhibits substantial spatial variability. Identical concentration of total nitrogen in surface water may provide nutrients for crops in agriculturally intensive areas, but it can also pose a significant threat to ecological equilibrium in protected areas. Transparent acknowledgment of the methodological and data-related limitations is crucial, as it not only elucidates the constraints of the current study but also identifies critical areas for future research, thereby facilitating scientific progress.

Supplementary Figures

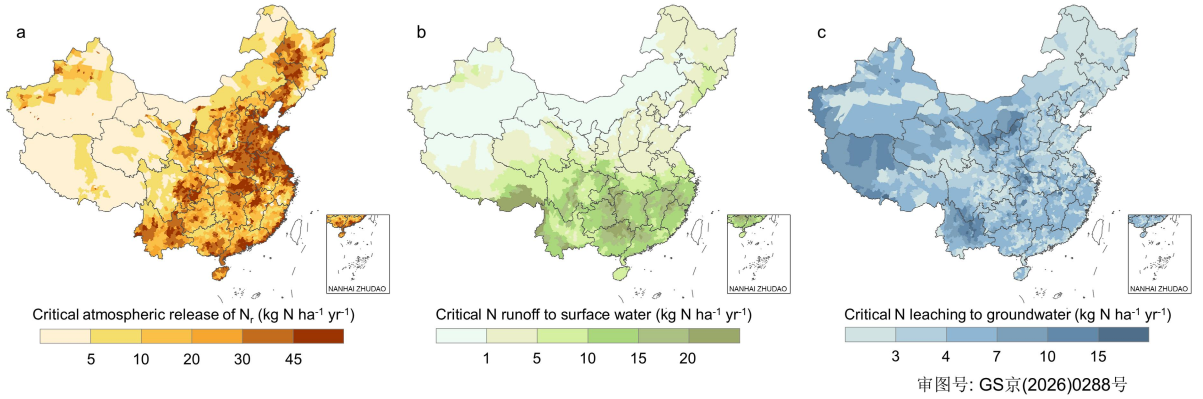

Figure S1. Spatial variation of safe nitrogen boundary in China.
a, Critical atmospheric release of N_r; **b,** Critical nitrogen runoff to surface water; **c,** Critical nitrogen leaching to groundwater. Hong Kong, Macau, and Taiwan are presented in white because corresponding data are unavailable, rather than representing zero values.

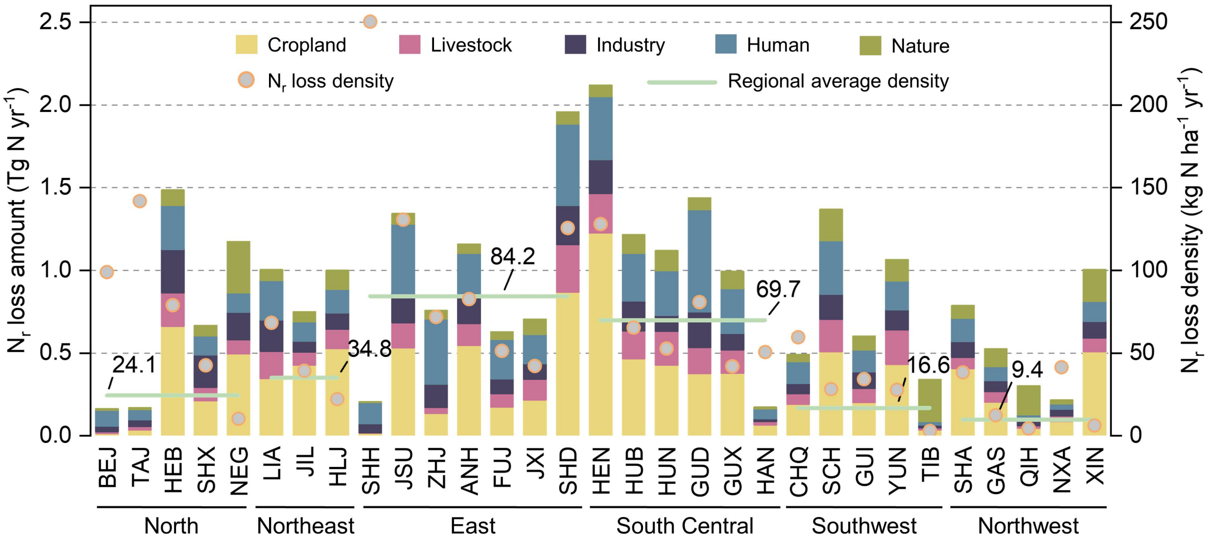

Figure S2. N_r loss amount and density at provincial scale in 2020.
The left axis represents N_r loss amount, and the right represents the density. The acronyms for all provinces are listed in Table S7.

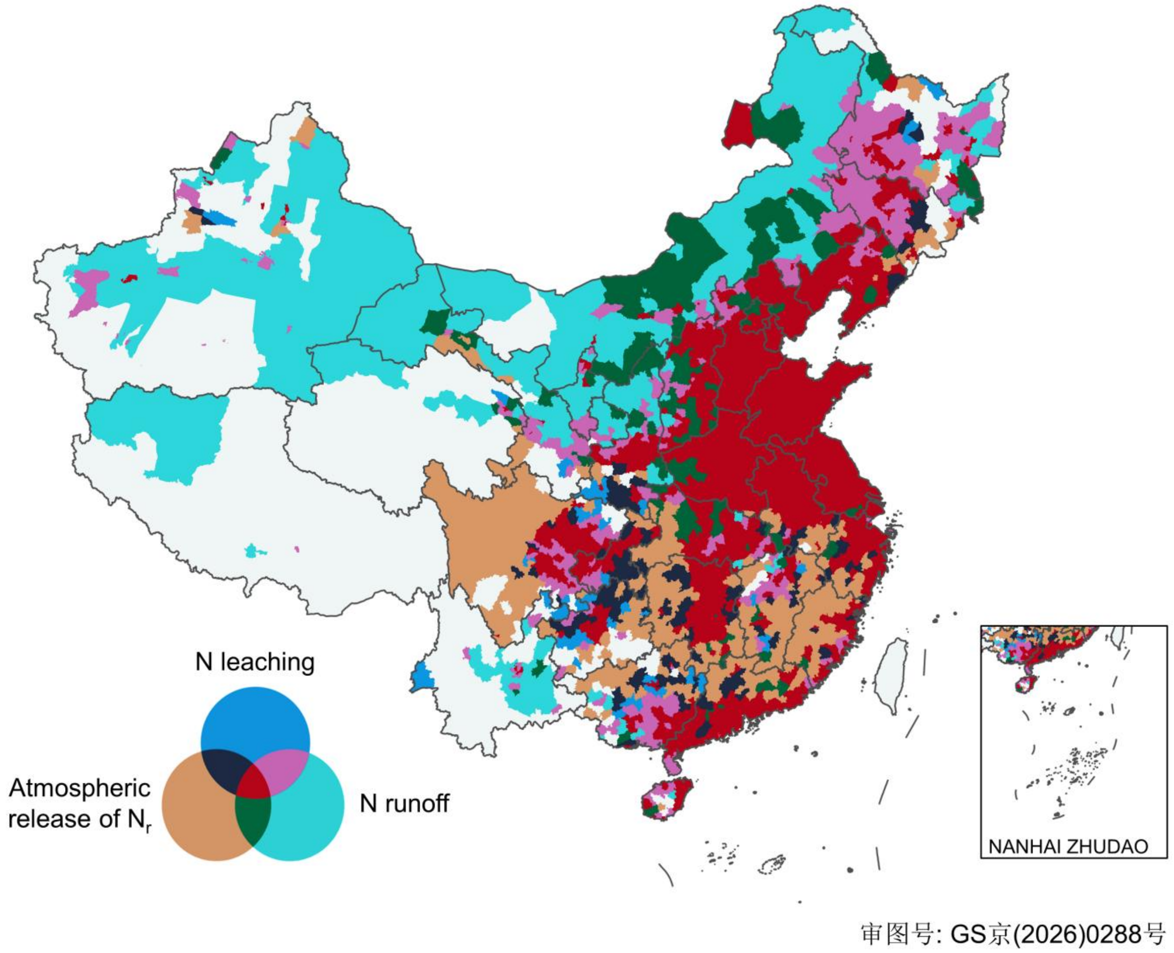

Figure S3. Spatial variation for the type of nitrogen-related boundary that has been exceeded.
The colors indicate exceedance of none (white), one, two or all three safe nitrogen boundaries (see legend). Hong Kong, Macau, and Taiwan are presented in white because corresponding data are unavailable.

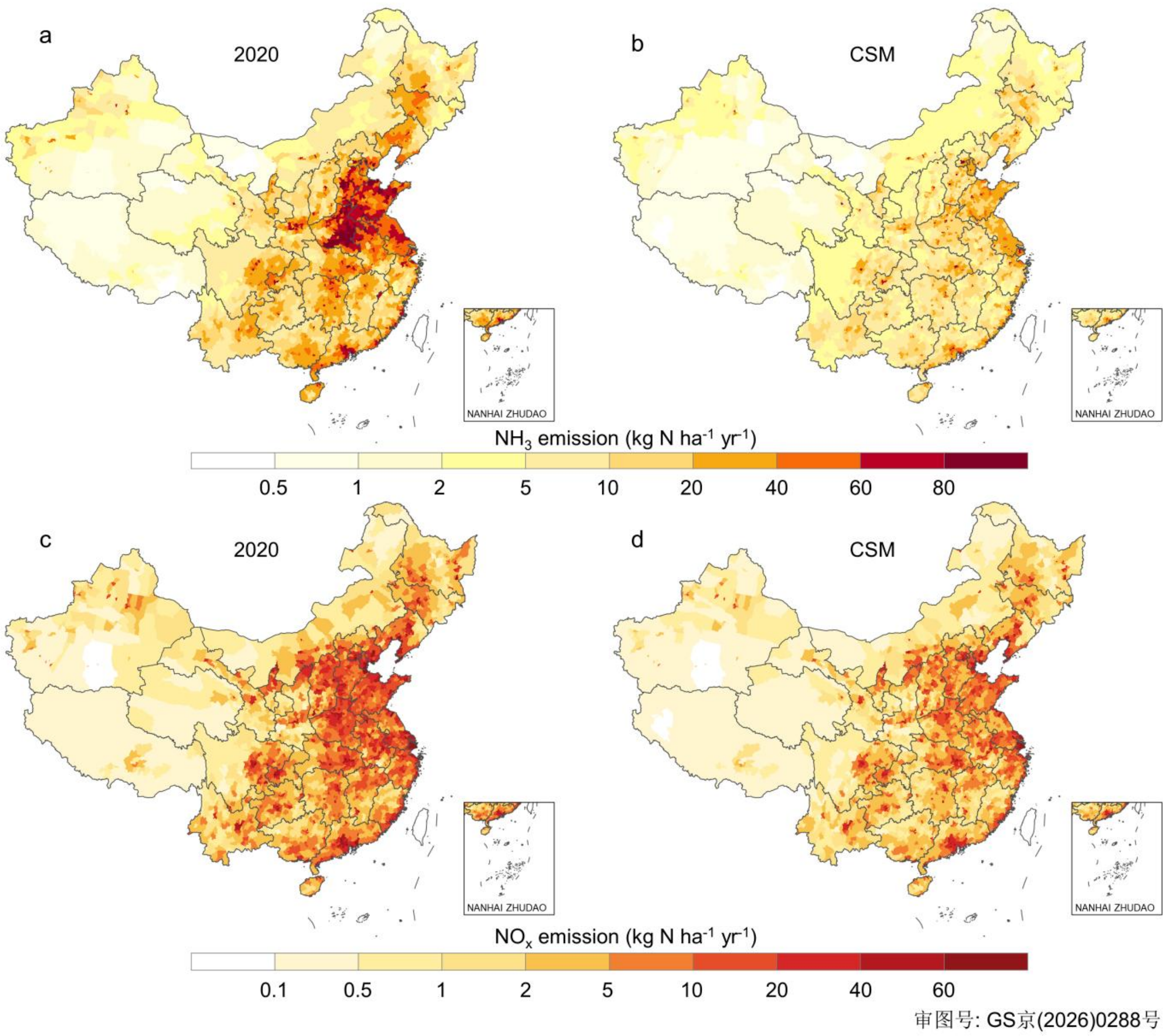

Figure S4. N_r losses to air across China at the county-level in 2020 and under CSM.
a-b, NH_3 emission intensity in 2020 (**a**) and under CSM (**b**). **c-d,** NO_x emission intensity in 2020 (**c**) and under CSM (**d**). Hong Kong, Macau, and Taiwan are presented in white because corresponding data are unavailable, rather than representing zero values.

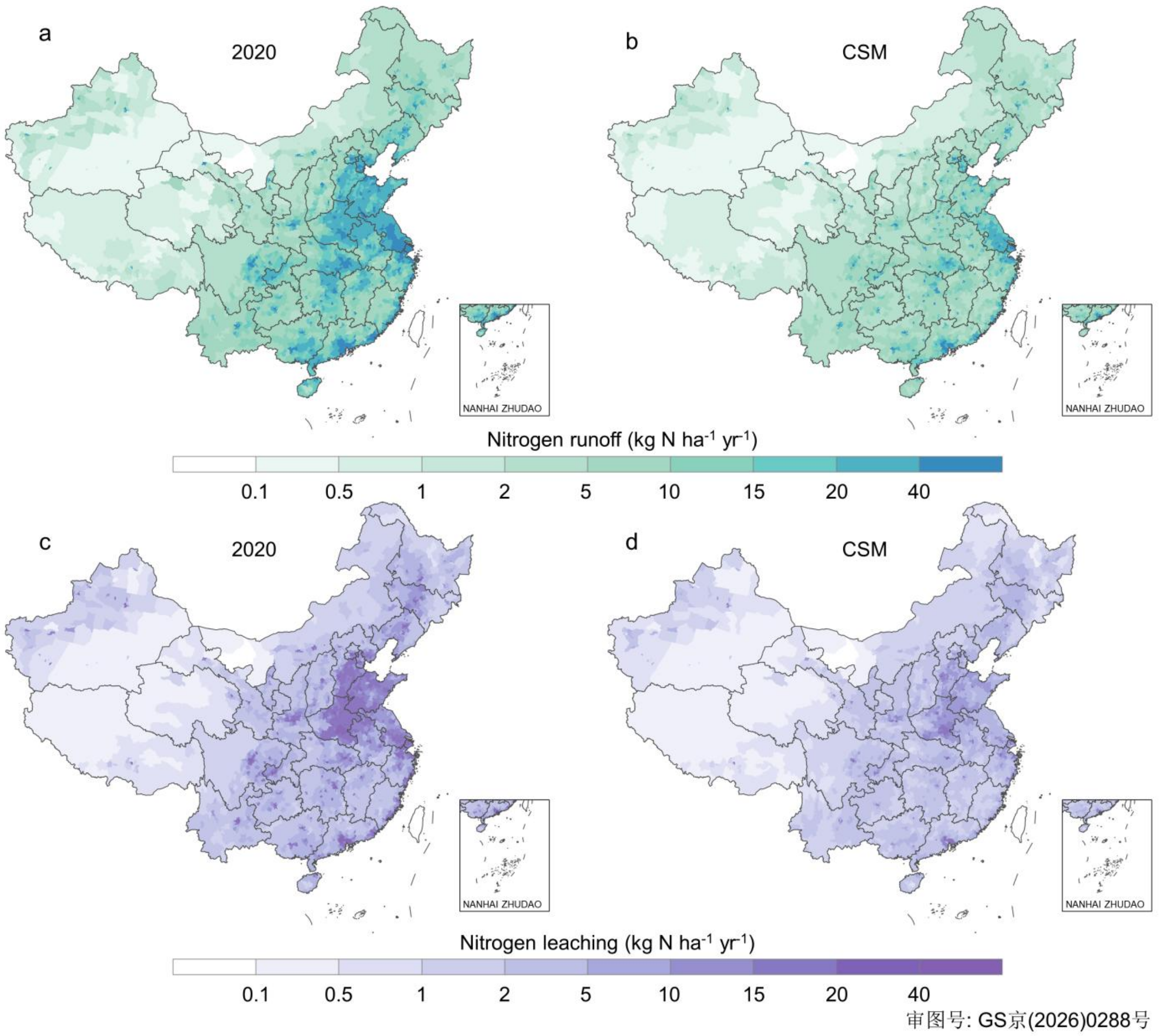

Figure S5. N_r losses to water across China at the county-level in 2020 and under CSM. **a-b,** Nitrogen runoff intensity in 2020 (**a**) and under CSM (**b**). **c-d,** Nitrogen leaching intensity in 2020 (**c**) and under CSM (**d**). Hong Kong, Macau, and Taiwan are presented in white because corresponding data are unavailable, rather than representing zero values.

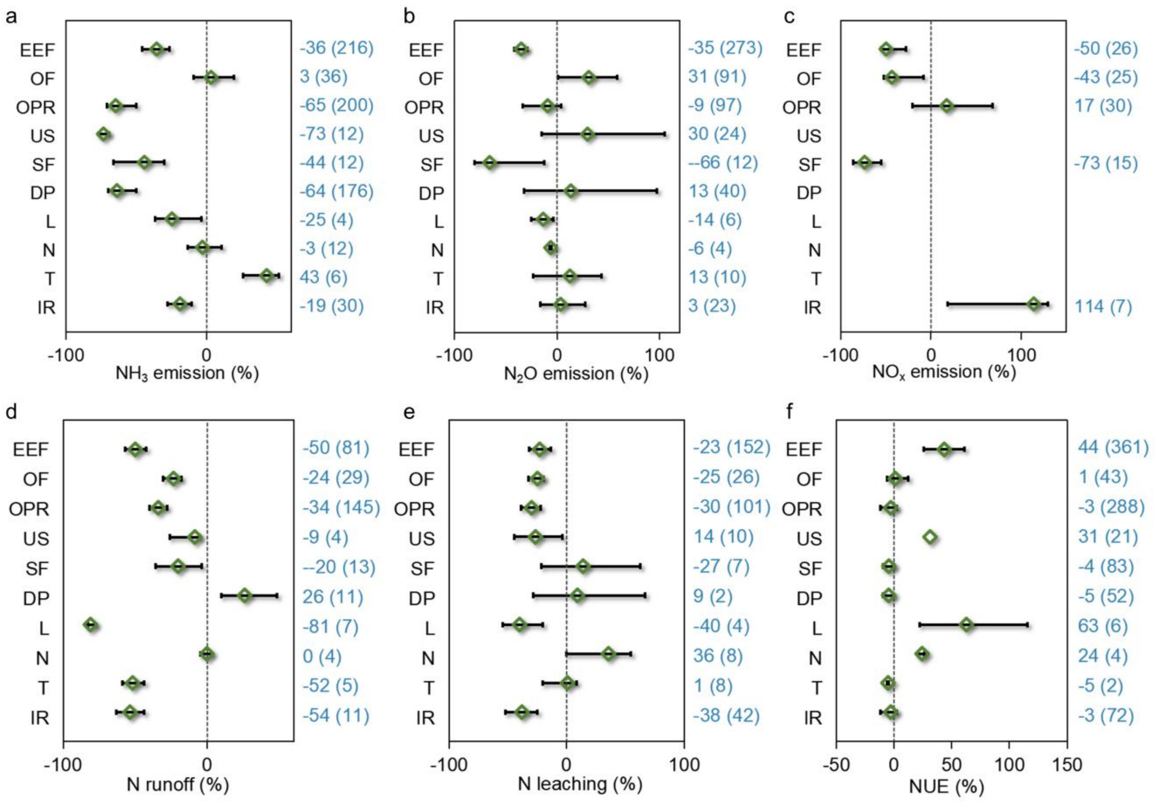

Figure S6. Effects of management practices in cropland sector for N_r use and loss. The results are presented as the mean and 95% bootstrap confidence intervals. Effects were significant ($p < 0.05$) if the confidence intervals did not overlap with zero. Negative values indicate a reduction in the amount of nitrogen loss or use due to the management practice whereas positive values indicate an increase in loss or use. Blue numbers show the exact mean value of change, with the sample size in brackets. The explanation of the abbreviation and their detailed description are shown in Table S4.

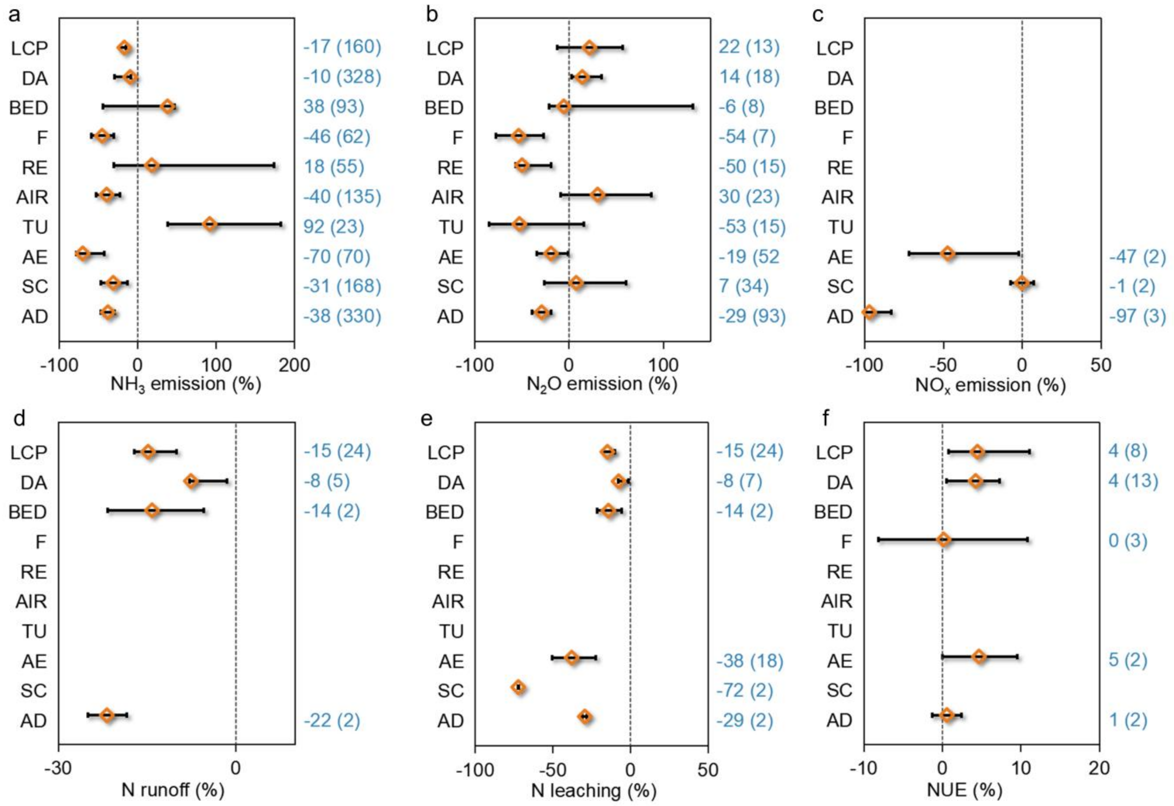

Figure S7. Effects of management practices in livestock sector for N_r use and loss.
The results are presented as the mean and 95% bootstrap confidence intervals. Effects were significant ($p < 0.05$) if the confidence intervals did not overlap with zero. Negative values indicate a reduction in the amount of nitrogen loss or use due to the management practice whereas positive values indicate an increase in loss or use. Blue numbers show the exact mean value of change, with the sample size in brackets. The explanation of the abbreviation and their detailed description are shown in Table S5.

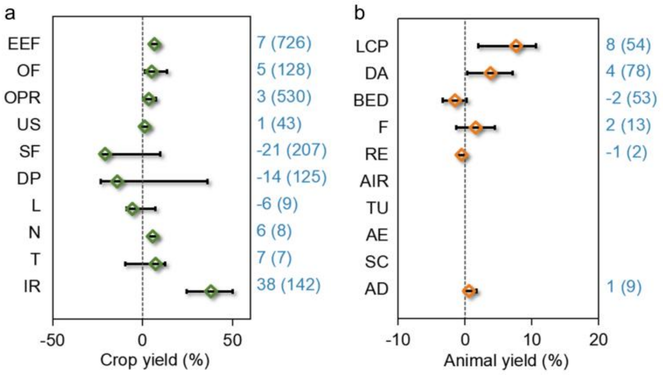

Figure S8. Effects of management practices for crop or animal yield.
The results are presented as the mean and 95% bootstrap confidence intervals. Effects were significant ($p < 0.05$) if the confidence intervals did not overlap with zero. Negative values indicate a reduction in the amount of crop or animal yield due to the management practice whereas positive values indicate an increase in yield. Blue numbers show the exact mean value of change, with the sample size in brackets. The explanation of the abbreviation and their detailed description are shown in Table S4.

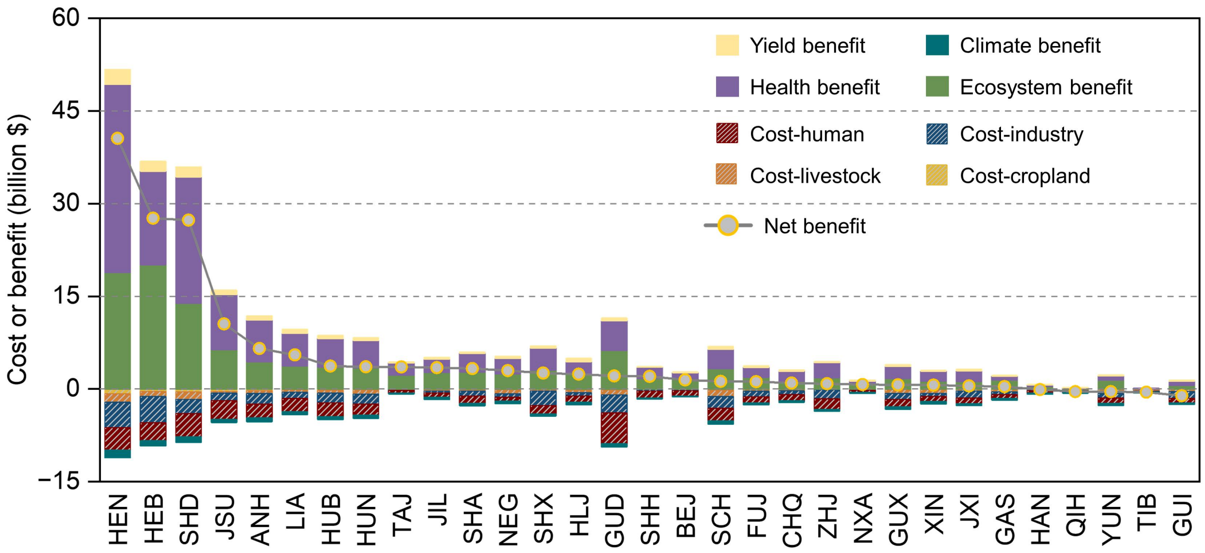

Figure S9. Total costs and benefits variations across 31 provinces in China under CSM. Negative values refer to costs or negative benefits, and positive values refer to benefits. The acronyms for all provinces are listed in Table S7.

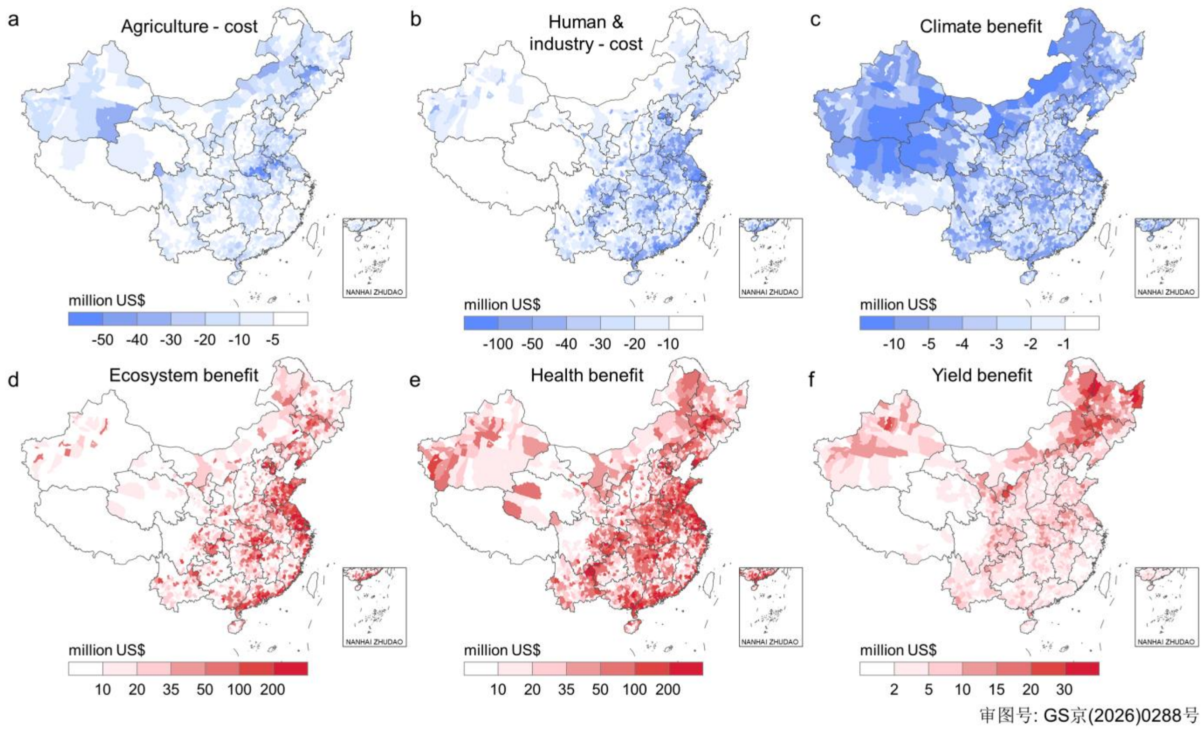

Figure S10. Geographic distribution of costs and benefits in China under CSM.
a-b, Geographic distribution of implementation costs for the agricultural (cropland and livestock) sector (**a**) and the human & industrial sector (**b**) under CSM. **c-f,** Geographic distribution of monetized benefits associated with climate (**c**), ecosystem (**d**), human health (**e**) and economic benefits derived from yield increases (**f**) under CSM. Blue indicates implementation costs, while red represents benefits. Hong Kong, Macau, and Taiwan are presented in white because corresponding data are unavailable, rather than representing zero values.

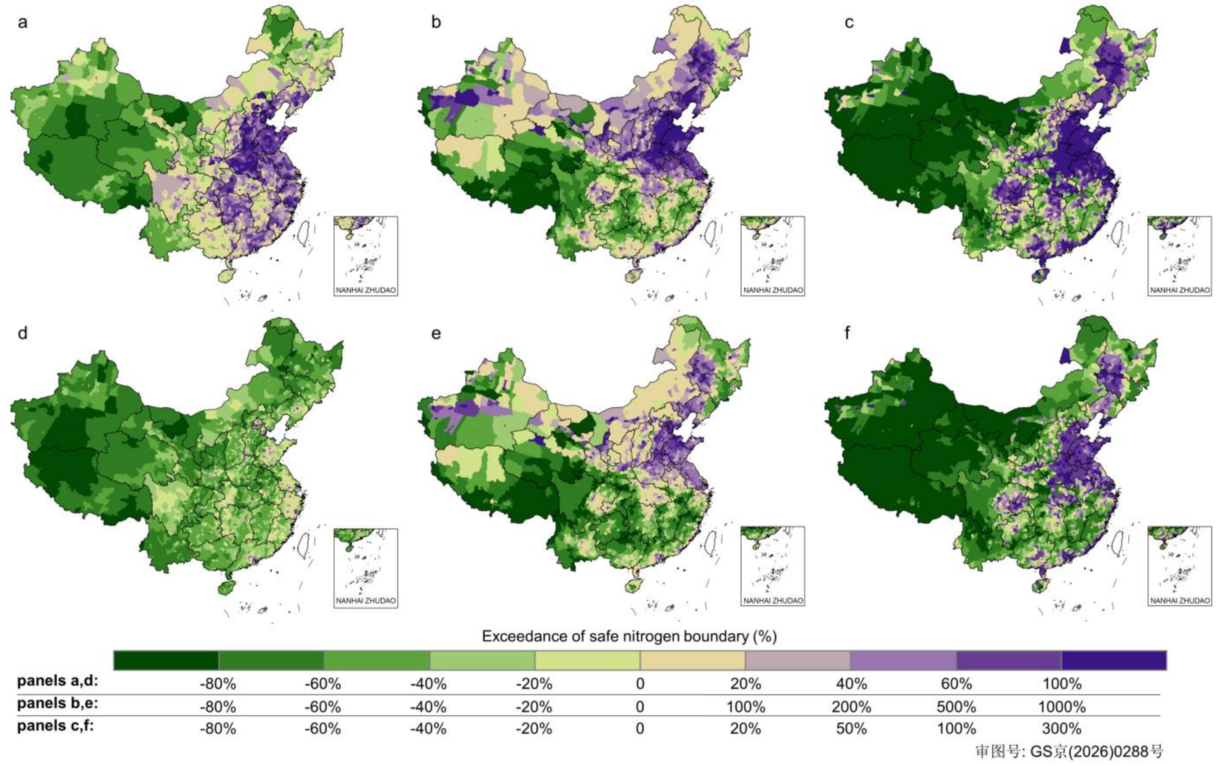

Figure S11. Spatial variation for the exceedance percentage of safe nitrogen boundary at the county-level in 2020 and under CSM.

a-c, the exceedance percentage of critical atmospheric release of N_r, nitrogen runoff to surface water and leaching to groundwater in 2020, respectively; **d-f,** the corresponding exceedance percentage under CSM. Hong Kong, Macau, and Taiwan are presented in white because corresponding data are unavailable, rather than representing zero values.

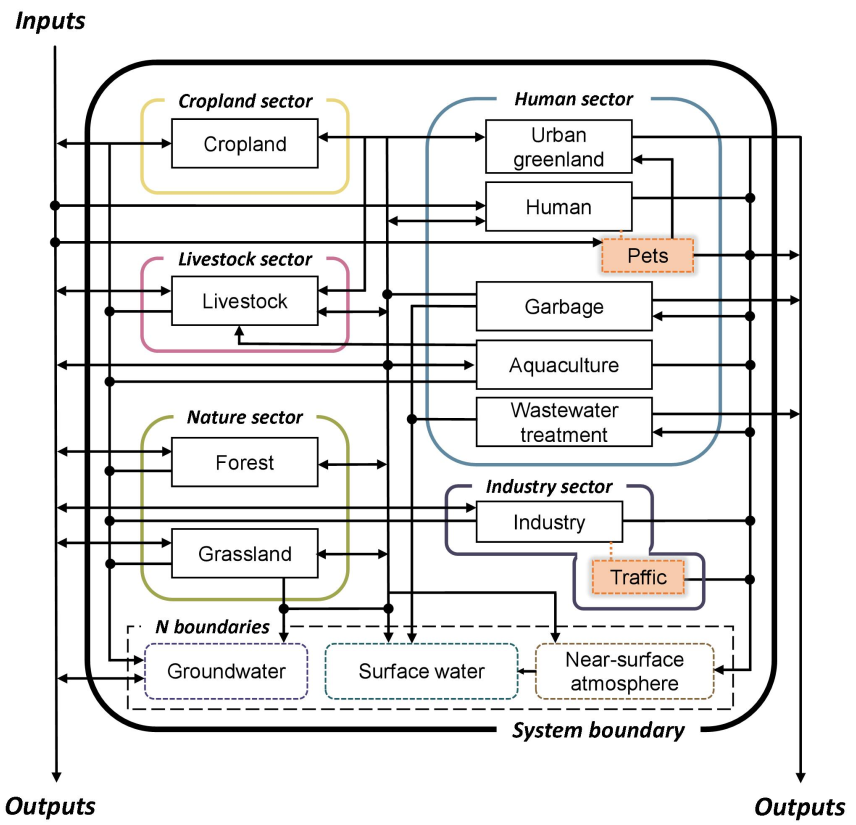

Figure S12. The framework of CHANS model and sector division in this study.

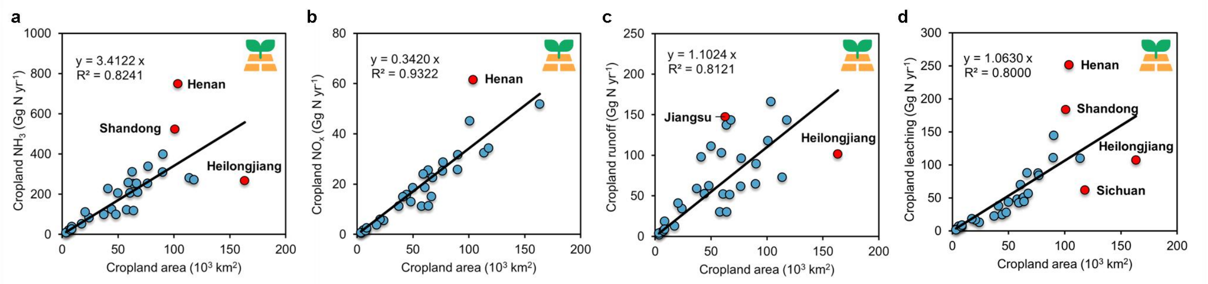

Figure S13. Different forms of N_r losses in the cropland sector as functions of corresponding driving factors.

(**a**), (**b**), (**c**), (**d**) denotes the correlation between cropland area and cropland NH_3 emission, NO_x emission, nitrogen leaching and runoff, respectively. The value of R^2 indicates the strength of the correlation between these variables. The red dots represent provinces with unique circumstances; however, they were included in the regression analysis. The icon was applied from https://www.iconfont.cn/.

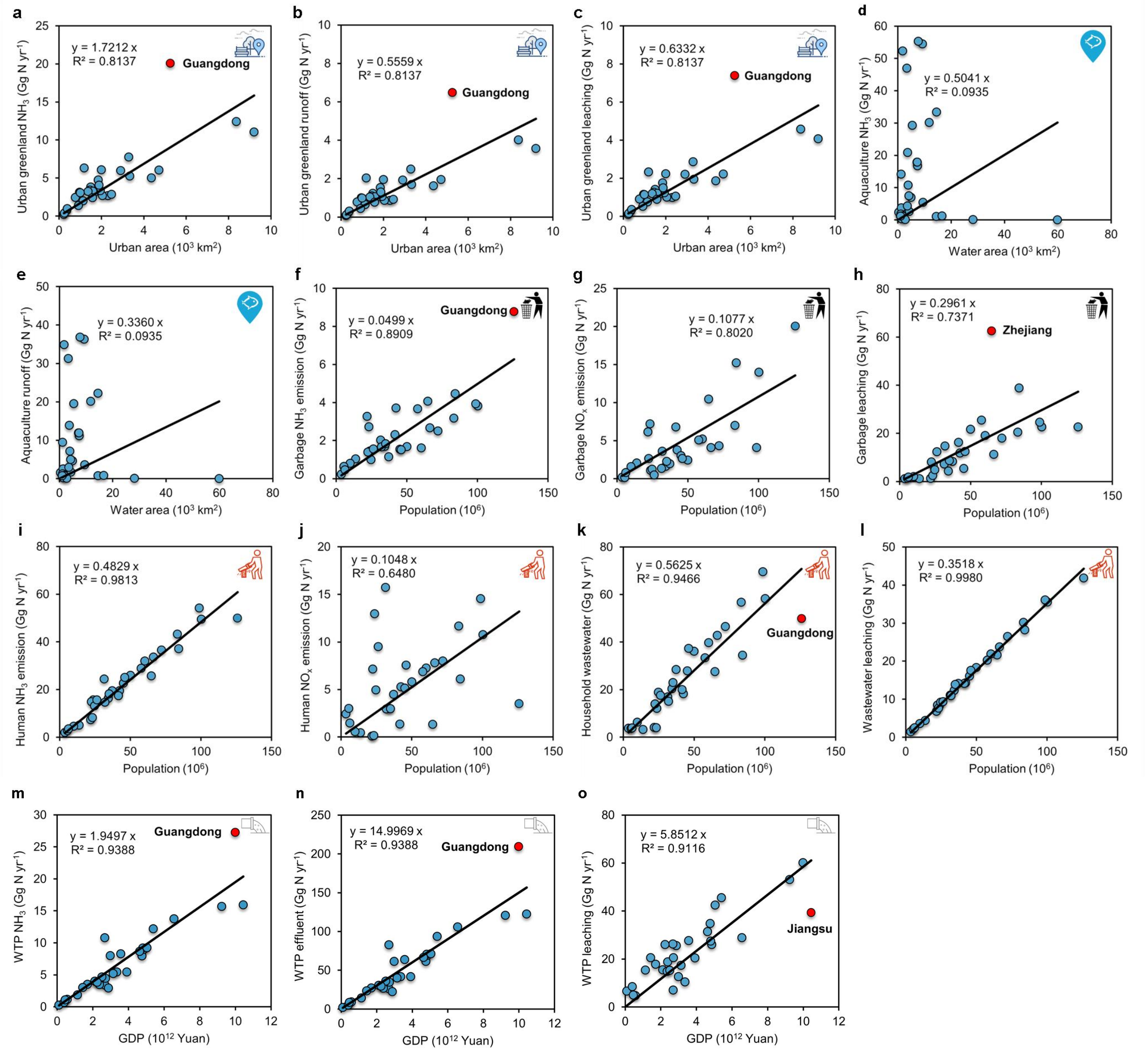

Figure S14. Different forms of N_r losses in the human sector as functions of corresponding driving factors.

(**a**), (**b**), (**c**) denotes the correlation between urban area and urban greenland NH_3 emission, nitrogen leaching and runoff, respectively. (**d**), (**e**) denotes the correlation between water area and aquaculture NH_3 emission and nitrogen runoff, respectively. (**f**), (**g**), (**h**) denotes the correlation between population and garbage NH_3 emission, NO_x emission and nitrogen leaching, respectively. (**i**), (**j**), (**k**), (**l**) denotes the correlation between population and human NH_3 emission, NO_x emission, nitrogen runoff and leaching, respectively. (**m**), (**n**), (**o**) denotes the correlation between GDP and WTP NH_3 emission, WTP nitrogen effluent and leaching, respectively. The value of R^2 indicates the strength of the correlation between these variables. The red dots represent provinces with unique circumstances; however, they were included in the regression analysis.The icons was applied from https://www.iconfont.cn/.

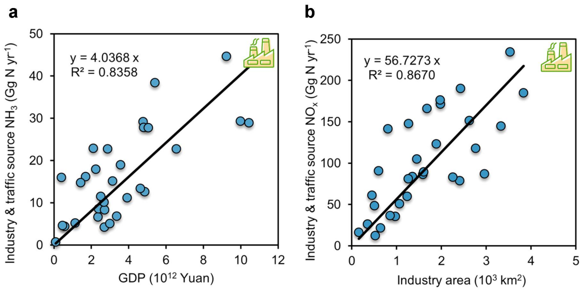

Figure S15. Different forms of N_r losses in the industry sector as functions of corresponding driving factors.

(**a**) denotes the correlation between GDP and NH_3 emission from the industry sector. (**b**) denotes the correlation between industry area and NO_x emission from the industry sector. The value of R^2 indicates the strength of the correlation between these variables. The icon was applied from https://www.iconfont.cn/.

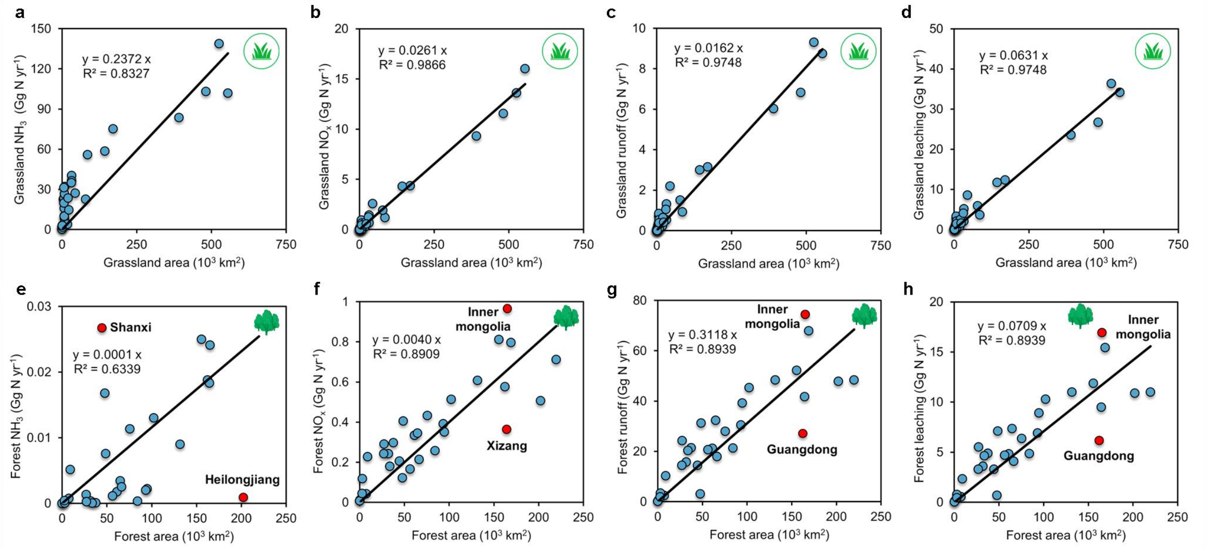

Figure S16. Different forms of N_r losses in the nature sector as functions of corresponding driving factors.

(**a**), (**b**), (**c**), (**d**) denotes the correlation between grassland area and grassland NH_3 emission, NO_x emission, nitrogen leaching and runoff, respectively. (**e**), (**f**), (**g**), (**h**) denotes the correlation between forest area and forest NH_3 emission, NO_x emission, nitrogen leaching and runoff, respectively. The value of R^2 indicates the strength of the correlation between these variables. The red dots represent provinces with unique circumstances; however, they were included in the regression analysis. The icons was applied from https://www.iconfont.cn/.

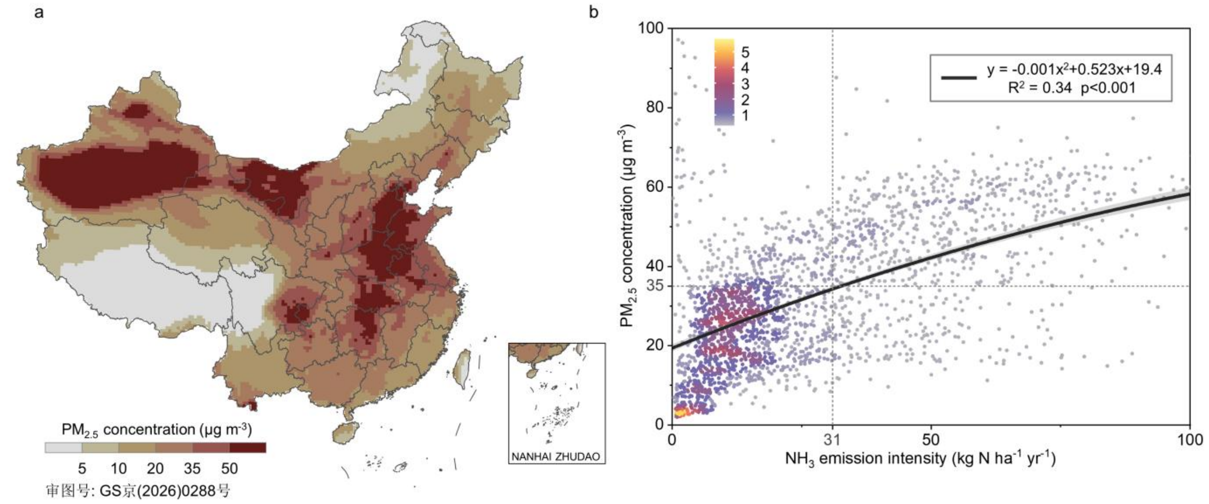

Figure S17. $PM_{2.5}$ concentration in China for 2020 and its correlation with NH_3 emission intensity at the county level.

a, Spatial variation of $PM_{2.5}$ concentration in 2020. The result was simulated using the WRF-Chem model, based on the estimated NH_3 and NO_x emissions from this study. **b**, The relationship between NH_3 emission intensity and $PM_{2.5}$ concentration at the county level. Each dot represents different numbers of counties, indicated by its color (refer to the legend). The x-axis denotes the average NH_3 emission intensity, while the y-axis represents the $PM_{2.5}$ concentration for each county in 2020.

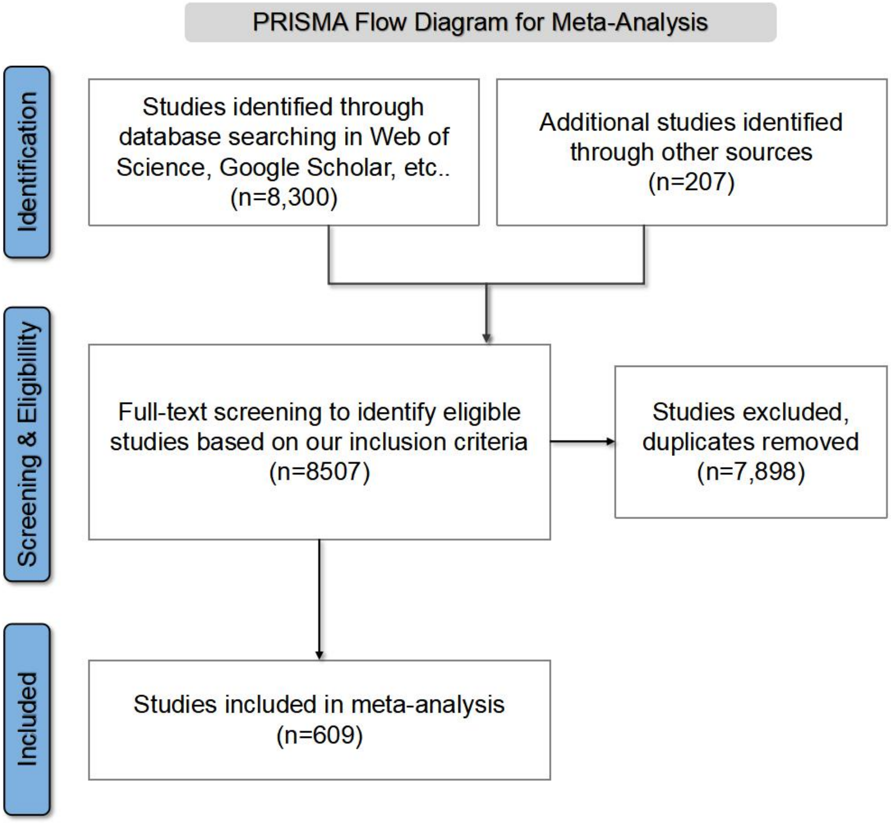

Figure S18. A PRISMA (Preferred Reporting Items for Systematic Reviews and Meta-Analyses) flow diagram of meta-analysis.

This diagram delineates the flow of information, indicating the number of relevant publications at various stages of the meta-analysis process, including "Identification," "Screening & Eligibility," and "Inclusion."

Supplementary Tables

Table S1. Comparison of alternative indicators for defining safe nitrogen boundaries

Indicator	Definition	Advantages	Disadvantages
Total nitrogen inputs	The total amount of reactive nitrogen entering a system over a given period, typically including Haber-Bosch nitrogen fixation (HBNF), biological nitrogen fixation (BNF), fossil fuel combustion (FFC), and imported products.	Simple and transparent to quantify, with data that are relatively easy to measure and track. This indicator directly reflects the intensity of human intervention and represents the manageable nitrogen input to a system, making it well suited for large-scale comparisons such as national or global assessments of anthropogenic pressure on the nitrogen cycle.	Weakly linked to actual environmental impacts, as high nitrogen inputs do not necessarily translate into high losses and outcomes depend strongly on nitrogen use efficiency and management practices. And it does not account for nitrogen removed in harvested products, stored in soils, or internally recycled within the system, nor does it distinguish between losses to air and water or reflect differences in ecosystem sensitivity.
Nitrogen surplus	The difference between total nitrogen inputs and nitrogen removed in harvested products, representing the amount of nitrogen remaining within the system that has the potential to be lost to the environment.	Directly reflects potential environmental risk because surplus nitrogen constitutes the source that can eventually be lost to the environment, and can be readily calculated from nitrogen input and output data, making it widely applicable from field to regional scales and useful for evaluating management efficiency, without relying on nitrogen use efficiency metrics.	Does not directly represent actual nitrogen losses in a given year, as values are influenced by soil nitrogen storage and legacy effects, with substantial spatial and temporal variability. Represents potential rather than realized environmental risk and does not differentiate loss pathways, providing no distinction between nitrogen losses to air and to water.
Nitrogen losses to air and water	The actual amount of reactive nitrogen emitted to the atmosphere, such as NH_3, N_2O, and NO_x, or leached and discharged to surface water and groundwater in forms such as nitrate and dissolved nitrogen, thereby directly representing	Directly linked to ecological and human health impacts, distinguishing between air and water pollution pathways, and providing an impact-oriented basis for defining environmentally meaningful safe boundaries by constraining the actual load of reactive nitrogen the environment can absorb, with effects quantified through	Data intensive and methodologically complex, relying on emission factors, models, or monitoring with substantial uncertainty and strong spatial and temporal variability, which complicates consistent application across large spatial scales. Reflects final outcomes rather than upstream pressures, making it a lagging indicator that is difficult to manage proactively

	nitrogen entering the environment through both atmospheric and aquatic pathways.	critical concentrations or fluxes associated with water eutrophication and air pollution.	and not directly associated with controllable nitrogen input fluxes.

Table S2. Summary of critical nitrogen deposition loads for 12 main vegetation types in China

Vegetation type	Critical load (kg N ha^{-1} yr^{-1})	Main responses	Ref
Temperate coniferous forest	10	Decrease in soil microorganisms and the activities of soil polyphenol oxidase, cellulase and sucrase.	[104-106]
Subtropical coniferous forest	15	Reduced decomposition of decaying organic matter and needle K, Ca, and Mg content.	[107]
Warm mixed forest	10	Increases in exotic grasses, decline in native species and in mycorrhizal communities. The lichen community composition shifts from sensitive to more N-tolerant species.	[108]
Temperate deciduous forest	12.5	Decrease in soil microorganisms.	[104, 108, 109]
Subtropical evergreen broad leaved forest	20	Reduced nutrient discharge from decaying organic matter and the breakdown of lignin and cellulose.	[109-112]
Subtropical monsoon evergreen broad leaved forest	30	Altering the photosynthetic and physiological traits of the main plants in the understory.	[109, 113]
Steppe and grassland	15	Changes in plant species and increases in invasive grasses.	[108, 109, 113]
Tropical plantation	35	Changes in species composition and richness.	[108, 113]
Scrubland	7.5	Decrease in the diversity and density of arbuscular mycorrhizal spores.	[108]
Fens	15	Decrease in bryophytes and increase in vascular plants.	[108]
Alpine meadow and tundra	7.5	Decrease in lichens and bryophytes and increase in vascular plants.	[108]
Cropland	40	N deposition provides a new source of fertilizer for crop growth. However excessive deposition may result in a decrease of crop yield and quality by influencing soil properties and soil microorganisms together with acid rain.	[88, 109, 114]

Table S3. Proxy parameters for the downscaling of N_r loss inventory

System	Proxy parameter & R^2 for regression at provincal level			
	NH_3	NO_x	runoff	leaching
Cropland	Wang et al., 2021 [115]	Cropland area, 0.93	Wang et al., 2021 [115]	Wang et al., 2021 [115]
Livestock	Zhu et al., 2022 [116]	-	Zhu et al., 2022 [116]	Zhu et al., 2022 [116]
Grassland	Grassland area, 0.83	Grassland area, 0.99	Grassland area, 0.97	Grassland area, 0.97
Forest	Forest area, 0.63	Forest area, 0.89	Forest area, 0.89	Forest area, 0.89
Urban greenland	Urban area, 0.81	-	Urban area, 0.81	Urban area, 0.81
Aquaculture	Water area, 0.09	-	Water area, 0.09	-
Industry & traffic	GDP per capita, 0.84	Industry area, 0.87	-	-
Human	Population, 0.98	Population, 0.65	Population, 0.95	Population, 0.99
Garbage	Population, 0.89	Population, 0.80	-	Population, 0.74
WTP	GDP per capita, 0.94	-	GDP per capita, 0.94	GDP per capita, 0.91

*Note: The table's regression analysis results for R^2 are shown in Fig. S2-S5.

Table S4. Detailed description of the selected mitigation measures

System	Strategy	Measures	Abbr.	Description
Cropland	additive	Enhanced efficiency fertilizers	EEF	Enhanced efficiency fertilizers (EEFs), encompassing slow/controlled-release fertilizers and inhibitors such as urease inhibitors (e.g., NPBT) and nitrification inhibitors (e.g., DCD), were evaluated in comparison to their non-enhanced counterparts. EEFs mainly include polymer-coated and sulfur-coated urea formulations, which are designed to enhance crop yield and nitrogen use efficiency (NUE) simultaneously by synchronizing nitrogen release with plant requirements and mitigating nitrogen losses.
		Organic fertilizer	OF	The application of manure and soil amendments such as biochar, straw, lime, compost, and organic acids can enhance soil fertility and nitrogen uptake by plants.
	4R	Right rate-optimizing N rate	OPR	Optimizing nitrogen fertilizer rates typically involves synchronizing them with soil nitrogen supply and crop requirements, necessitating a direct reduction in nitrogen fertilizer application for certain crops.
		Right source-urea substitution	US	Substitute urea-based fertilizer with other N fertilizers such as ammonium sulphate, ammonium nitrate, urea phosphate, calcium ammonium nitrate, monoammonium phosphate and diammonium phosphate.
		Right time-split fertilization	SF	Compared to a single application, splitting the total amount of nitrogen fertilizer into 3-4 applications for basal fertilization and top dressing will significantly optimize the nitrogen use efficiency by coinciding with the times that the crops need fertilizer most.
		Right place-deep placement	DP	Deep placement can significantly decrease the NH_3 volatilization by typically applying the fertilizer at a depth of 20 cm beneath the plants.
	crop species	Legume rotation	L	Legume-cereal rotation involves growing legumes to provide complementary nitrogen in the soil for cereal crops.
		New cultivar	N	Improving crop varieties to enhance the efficient utilization of nitrogen from sustainable sources, such as organic fertilizers and biological nitrogen fixation, is an important tool for agronomy.
	biophysical	No-tillage	T	No-tillage refers to an agricultural practice of growing crops without tilling or disturbing the soil through operations like plowing or digging.

	management	Irrigation	IR	Typically refers to drip irrigation or fertigation, a technique that delivers nutrients directly to plant roots through a network of tubes and emitters. Compared to broadcast fertilization, it supplies nutrients and water more appropriately by conveying them directly to the active root zone.
Livestock	Feeding strategies	Low crude protein feeding	LCP	By adhering to contemporary feeding guidelines that mitigate excessive dietary protein intake, this strategy leads to a decrease in nitrogen excretion.
		Dietary additive	DA	The employment of dietary additives, such as acidifiers, probiotics, dried distillers' grains with soluble (DDGS), saponin and yucca extracts, has been demonstrated to yield a significant reduction in ammonia emissions.
	Animal housing strategies	Suitable bedding materials	BED	The utilization of appropriate bedding materials can effectively reduce nitrogen loss while promoting resourceful utilization. Common bedding options encompass a range of materials, including straw, wheat husk, rice husk, rice chaff, wood sawdust, and combinations of various organic substances.
		Floor adaption	F	Optimized flooring management encompasses options like slatted, slotted, and netting floors, which enable timely manure removal. Mainly applicable for intensive rearing of poultry and pigs.
		Frequent manure removal	RE	Frequent manure removal is facilitated through techniques such as scraping, flushing, pulling pit-drain plugs, belt transport, and pressure washing.
		Air cleaning or scrubbing	AIR	An air cleaning system, combined with an air scrubber, removes NH_3 from the air through NH_3 absorbent within a designated apparatus that directly captures and treats ammonia emissions. Additionally, chemical, acidic, or biofilters can be installed at exhaust outlets to adsorb or convert NH_3 gas as it passes through.
	Manure management strategies	Turning of manure	TU	This technique is primarily applied to solid manure management. Employing manure turners facilitates the uniform mixing of composted manure, which aids in water evaporation and improves aeration. This process will not only accelerate the composting process, but also significantly reduce odor emissions.
		Adjusted aeration modes	AE	Aeration is chiefly utilized in the management of liquid manure. Aeration systems forcefully inject air into sewage, enabling direct contact between sewage and air for oxygenation, thereby facilitating the oxidative breakdown of organic compounds in the sewage.
		Storage covers	SC	Using specific materials to create a cover layer on manure reduces nitrogen loss by preventing direct contact with air. Cover choice varies by manure type: solid

				manure needs compaction, liquid manure benefits from acidification and straw or artificial films, while lagoons and slurry tanks require floating or structural covers, respectively.
		Acidifiers or additives	AD	Directly adding acids, inhibitors, or absorbents to manure can effectively reduce emissions. The addition of acids converts NH_3 in manure to NH_4^+, decreasing NH_3 volatilization and increasing the ammonia content suitable for organic fertilizer application.
Industry	Pretreatment	Clinker substitution	CS	Replacing a portion of clinker with other cementitious materials has the potential to realise significant emissions reductions within cement sector.
		Improving fuel quality	IFQ	Utilizing coal with reduced ash and sulfur content.
	Combustion modification (CM)	Energy intensity and efficiency improvement	EII	By enhancing energy efficiency in industrial processes and adopting advanced technologies like preheater kilns, multi-channel combustion and heat recovery systems, production tasks can be achieved with reduced fossil fuel consumption, leading to decreased emissions, particularly NO_x that are related to combustion.
		Electrification	ELE	Electrification involves replacing fossil fuel combustion in industrial boilers with electricity generated from renewable sources (ideally sourced from wind, solar, hydro, and nuclear energy), thereby reducing emissions.
		Fuel switching	FS	Switching from high-nitrogen fuels like coal to cleaner alternatives such as natural gas or renewable energy sources can help reduce NO_x emissions, as plants using these cleaner fuels produce fewer emissions than those using coal.
		Fuel balancing	FB	Optimizing the operation of existing generating stations by increasing production from non-thermal power plants and decreasing production from some thermal power plants can successfully reduce NO_x emissions without structural changes to the fleet.
	Process improvement (PI)	Staged combustion	SC	Staged combustion is a method to reduce NO_x emissions during combustion by introducing over-fired air into a boiler or furnace. It includes air-staged and fuel-staged supply methods.
		Precise ammonia injection	PAI	Precise ammonia injection technology reduces NO_x emissions by accurately injecting ammonia into the exhaust gas stream. This precise control optimizes the reaction with NO_x, converting it to nitrogen and water, minimizing ammonia slip, and enhancing NO_x reduction efficiency.

		Selective catalytic reduction	SCR	Selective Catalytic Reduction is the most widespread state-of-the-art technology for NO_x mitigation, which employs heterogeneous catalysis to transform these pollutants into nitrogen (N_2) and water (H_2O). This process involves introducing a reductant—such as ammonia, hydrogen, hydrocarbons, or urea—into the flue gas stream in the presence of oxygen, before it enters a catalyst chamber where Fe-based catalysts facilitate a chemical reaction that effectively reduces NO_x emissions to harmless substances.
		Selective non-catalytic reduction	SNCR	Similar to SCR, this technique injects ammonia or other reducing agents into flue gas to control NO_x emissions. However, this technique does not require a catalyst, so reducing space needs. Recently, this method has seen significant improvements, making it particularly suitable for controlling emissions from smaller industrial boilers.
		Low-NOx burners	LNB	Replacing conventional burners with low-NO_x burners in industrial boilers and power plants significantly reduces NO_x emissions. These burners employ advanced combustion technologies, such as staged combustion, fuel burning, or flue gas recirculation, to optimize fuel-air mixtures and combustion conditions. By modifying air and fuel injection to delay mixing and reduce oxygen availability, low-NO_x burners lower peak flame temperatures and limit the time spent in high-temperature zones, thus curtailing NO_x formation while maintaining high combustion efficiency.
		Catalytic decomposition	CD	NO_x/N_2O abatement can be efficiently achieved through the direct decomposition of these pollutants using noble metal, spinel, perovskite-structured metal oxides, and ion-exchanged zeolite catalysts. These catalysts excel in dissociating nitrogen oxides without the need for reducing agents, offering a streamlined approach to pollution control.
		Electron beam	EB	Electron beam technology stands out for its ability to simultaneously remove SO_2 and NO_x, while producing by-products like fertilizer. The efficiency of NO_x removal can be further enhanced by incorporating microwave (MW) processes.
Traffic	Engine Technology (ET)	Internal engine modification	IEM	Internal engine modifications focus on optimizing combustion, enhancing air charge characteristics, or modifying fuel injection systems through engine modifications. These modifications primarily aim to reduce NO_x emissions by lowering peak temperature and pressure within the cylinder.

		Miller cycle	MC	Miller cycle reduces NO_x emissions through a lower compression ratio, high-pressure turbocharging, variable air inlet valve timing, and charge-air cooling, which can effectively decrease the temperature in the combustion chamber.
		Water injection	WJ	By adding water directly into the engine cylinders after fuel injection, water injection can effectively reduce NO_x emissions through cooling the combustion chamber and lowering combustion temperatures.
	New Energy technology (NE)	New-energy vehicle	NE	Replacing traditional fuel-powered transportation with new energy options like electric vehicles.
	Exhaust gas capture & control (ECC)	Diesel oxidation catalysts	DOC	Diesel oxidation catalysts (DOCs) facilitate the oxidation of harmful gases into less harmful compounds and heat exhaust gases to aid in the active regeneration of DPF. Additionally, the production of NO_2 supports the passive regeneration of DPF and enhances SCR efficiency.
		Diesel particulate filters	DPF	Diesel Particulate Filters (DPF) in the transport system use specialized filters to capture and trap particulate matter (PM) emissions from diesel engines as exhaust gases pass through.
		Selective catalytic reduction	SCRT	The selective catalytic reduction (SCR) method employs a catalyst to reduce nitrogen oxides (NOx) emissions. In this process, a reductant, typically ammonia (NH_3) or urea, is injected into the flue gas stream before it enters a catalyst chamber. The catalyst facilitates a chemical reaction between the NO_x and the reductant, converting the NO_x into harmless nitrogen (N_2) and water (H_2O).
		Exhaust gas recirculation	EGR	Recirculating exhaust gases replace some fresh intake air with exhaust gas, lowering oxygen levels in the combustion chamber. This, along with the added heat capacity, suppresses temperature peaks, reducing nitrogen oxide emissions by up to 30% without increasing fuel consumption.
	Drag reduction technology (DR)	Drag reduction	DR	Drag reduction involves minimizing vehicle resistance through lightweight, low rolling resistance tires, and low-friction lubricants.
Human	Household fuel combustion	Electric stove	ES	Replacement of existing gas stove with electric stove.
		Ventilation hood	VH	Installation of ventilation hood over existing gas stove.
		High-efficiency	HEPA	The placement of air purifiers with high-efficiency particulate air and carbon

	(HF)	particulate air filters		filters.
		Promote alternatives to burning	PAB	Promoting alternatives to burning involves reducing biomass energy consumption and encouraging practices like composting, mulching, or utilizing crop residues as animal feed.
	Advanced toilet (AT)	Innovative or advanced septic	IAS	I/A septic systems utilize innovative technologies to convert nitrogen to N_2 through biochemical processes. These on-site wastewater treatment systems offer higher treatment efficiencies that reduce environmental impact, which are crucial for sustainable wastewater treatment in areas without centralized sewer systems.
		Urine-diverting toilet	DT	A diversion toilet, like a urine-diverting or split toilet, separates urine and feces at the source using two independent compartments.
		Composting toilets	CT	Composting toilets utilize natural decomposition processes to break down human waste into compost, rather than flushing waste into septic tanks or sewer systems. These eco-friendly toilets promote sustainable waste management by converting waste into nutrient-rich compost for soil enrichment.
		Improved sanitation infrastructure	ISI	With the advancement of economic development, there is a trend toward constructing improved sanitation infrastructure to reduce sewage pipeline leakage rates.
	Waste recycling (WR)	Efficient recycling of human excreta	ERE	Efficient recycling of human excreta involves utilizing technologies to recover nutrients from source-separated human urine, which accounts for 80% of nitrogen excretion.
		Less household waste and recycling	LHWR	In the scenario, less household waste and recycling,' there is an assumed reduction of the waste share to 20%. Additionally, half of the nutrients from household waste and sewage are recycled as fertilizers, promoting sustainable nutrient management practices.
		Domestic sewage collection	DSC	Domestic sewage collection aids in reducing N_r pollution by directing wastewater to treatment plants, where nitrogen compounds are removed through processes such as denitrification. In our modeling, household connectivity rates are scaled with regional economic status, meaning that wastewater collection and subsequent nitrogen removal reflect locally feasible infrastructural and socioeconomic conditions.
Garbage	Incineration	Improved	IW	Improved incineration process encompasses various aspects such as incineration

	(IN)	incineration process		gas capture, waste-to-energy technologies, and advancements in combustion processes. These improvements not only enhance energy recovery but also contribute to reducing emissions
		Replace landfill with composting and incineration	RCI	Future waste management will shift towards advanced incineration technologies like fluidized bed and grate furnaces. The market for small-scale incinerators is expected to decline as larger incineration equipment becomes more prevalent.
	Landfill (LF)	Negative pressure steam-stripping pretreatment + Membrane bioreactor	NPS+ MBR	This technology involves injecting gas into leachate to transfer pollutants to the gas phase, effectively removing NH_3-N before biological treatment. Additionally, Membrane bioreactor (MBR) systems, combining biofilm and ultrafiltration, have also been proven to be practical and efficient for leachate treatment.
		Coagulation pretreatment + mechanical vapor recompression	CP+M VR	The utilization of mechanical vapor recompression in conjunction with coagulation pretreatment enhances the efficiency of the treatment process.
		Electrocoagulation combination with biological treatment	EC+B T	The combination of electrocoagulation with biological treatment effectively removes ammonium-nitrogen from wastewater. This approach, combined with ozonation, enhances the removal efficiency of organic compounds and ammonium-nitrogen in mature leachates. The synergy of electrocoagulation and ozonation, along with biological treatment, offers a promising strategy for improving the removal of TOC and ammonium-nitrogen in landfill leachate treatment processes.
		UV/H_2O_2-BAC	UVH+ BAC	The UV/H_2O_2-BAC treatment effectively removes organic matter and nitrogen from high-salinity reverse osmosis concentrate (ROC), ensuring safe and sustainable wastewater treatment.
Wastewater (WTP)	Physical methods	Step-feed SBR	SFS	The step-feed SBR effectively controls N_2O production during nitrogen removal via managing ammonia and nitrite levels in domestic wastewater. Anoxic denitrification using organic matter as a carbon source reduces N_2O production significantly. Compared to the aerobic-anoxic SBR, the step-feed SBR shows a 50% decrease in total N_2O production during nitrogen removal via nitrite, demonstrating its efficiency in managing and minimizing N_2O emissions.
		Membrane	MF	Membrane filtration utilizes semi-permeable membranes under a pressure gradient

	filtration		to prevent the transfer of solids and dissolved components from influent to effluent, effectively removing pollutants like ammonia. These membranes, often microporous and hydrophobic, facilitate mass transfer in liquid-liquid or gas-liquid phases without dispersion. Techniques such as reverse osmosis, ultrafiltration, microfiltration, and nanofiltration, along with the development of hollow fiber membrane contactors, offer versatile solutions for wastewater treatment by capturing volatile contaminants through absorption and stripping processes.
	Addition of Cu nanoparticles	ACU	Operating SBR reactors in an anoxic-aerobic mode with the presence of copper ions (Cu^{2+}) in the sludge fermentation liquid enhances Nos enzyme activity, leading to a significant reduction in N_2O production. Adding copper to the denitrification tank increases N_2OR enzyme activity, reducing N_2O emissions by 50-73%.
Chemical methods	Chemical precipitation	CPR	Chemical precipitation, especially struvite formation, efficiently removes ammonia from high-concentration wastewater. This process forms particles that settle and are removed, minimizing sludge production and operating costs. It's applicable on various scales, from small to large treatment systems, and offers a fast, energy-efficient solution with low capital investment.
	Ion exchange method	IE	The ion exchange method effectively removes ammonia and other nitrogen pollutants from wastewater by swapping dissolved ions with similarly charged ions. This process, suitable for a wide range of ammonia concentrations and temperatures, utilizes cation and anion exchangers for targeted ion removal. Zeolites, with high sorption and selectivity, have shown high efficiency in ammonia removal, making ion exchange a viable option for enhancing water quality through nitrogen pollutant reduction.
	Adsorption	ADS	The adsorption process utilizes activated carbon and natural zeolites like clinoptilolite to remove ammonia and other nitrogen pollutants. These adsorbents, chosen for their vast surface area and polar properties, efficiently attract and bind ammonia, especially under optimal pH conditions. While activated carbon is traditionally used, modifications and composite adsorbents have been developed to enhance ammonia adsorption capacity.
	Breakpoint chlorination	BC	Breakpoint chlorination, conducted within a pH range of 6.5 to 7.5, is a highly effective method for the removal of nitrogen pollution from wastewater. This

			process oxidizes 95-99% of ammonia, transforming it into nitrogen gas.
	Bioreactors	BR	Bioreactors, utilizing the biological treatment capabilities of nitrifying and denitrifying bacteria, effectively convert reactive nitrogen in water into N_2. This process is enhanced in membrane bioreactors, where membrane filtration complements biological treatment by acting as a physical barrier.
	Nitrification/denitrification sequencing batch reactor	SBR	The shortcut nitrification/denitrification (Nit/DNit) sequencing batch reactor process treats wastewater effectively by employing intermittent aeration and carbon source addition, significantly reducing NO_2- accumulation and cutting N_2O emissions by approximately 99%. This method leverages the inherent kinetic differences between nitrobacteria and nitrite bacteria to control the nitrification reaction to halt at the NO_2^--N stage, leading to substantial NO_2^--N accumulation before proceeding to denitrification.
	Control sludge retention time	SRT	Controlling the Sludge Retention Time (SRT) in treatment systems effectively reduces N_2O emissions by favoring the growth of Nitrite Oxidizing Bacteria (NOB). This approach lowers NO_2 concentrations and subsequently decreases N_2O production.
Biological methods	Bioscrubber	BS	A bioscrubber, utilizing methanol as a carbon source, offers an innovative approach to wastewater treatment by effectively removing nitrogen pollutants. By harnessing the power of microorganisms that thrive on methanol, the bioscrubber efficiently breaks down nitrogenous compounds, transforming them into less harmful substances.
	Aerobic/Anaerobic bacteria	AA	Biological treatment efficiently reduces nitrogen compounds in wastewater through the action of aerobic and anaerobic ammonia-oxidizing bacteria. Aerobic bacteria consume oxygen to convert ammonium into nitrite and then nitrate, which is further reduced to nitrogen gas using external electron acceptors like methanol. Conversely, anaerobic bacteria operate under anoxic conditions, using nitrite as an electron donor to transform ammonia directly into nitrogen gas. This cost-effective, natural process leverages the distinct metabolic pathways of these bacteria groups, offering a significant advantage over other treatment technologies by spontaneously driving the reactions with negative Gibbs' free energy, ensuring the efficient removal of nitrogen from wastewater.
	Biological	AWTP	In aquaculture aqueous systems, biological methods play a crucial role in removing

	methods for aquaculture	nitrogen pollution from wastewater. These methods primarily rely on the natural processes of nitrification and denitrification carried out by specific bacteria.

Table S5. Mitigation potential and implementation cost of the selected mitigation measures

Abbr.	Mitigation potential					Implementation cost	Ref
	NH_3	NO_x	N_2O	runoff	leaching		
**EEF**	36% (26-46%)	50% (28-55%)	35% (29-41%)	50% (43-57%)	23% (13-32%)	Enhanced efficiency fertilizers (EEF) have varying costs: Controlled-release fertilizers (CRF) are priced at 350 $/t, nitrification inhibitors (DCD) at 1 $/kg, and urease inhibitors (NBPT) at 20 $/kg, which cost 50% to 1200% more than traditional nitrogen fertilizers on average.	[117-119]
**OF**	-3% (-19-9%)	43% (9-53%)	-31% (-59-(-1)%)	24% (18-31%)	25% (20-33%)	Costs vary based on material type, quality, moisture content, freight, and additional agricultural practices. The average market price for organic fertilizer is 150 $/t, with additional labor required. Here we use biochar (400 $/ t) to represent all kinds of amendments.	[120-122]
**OPR**	65% (50-71%)	-17% (-68-86%)	9% (-4%-34%)	34% (28-40%)	30% (33-39%)	Optimizing nitrogen rates involves no additional cost, with reductions based on recommended crop-specific rates. Fertilizer utilization efficiency is expected to increase to over 40%, enhancing cost-effectiveness and productivity.	
**US**	73% (70-77%)	-	-30% (-105%-15%)	9% (6-26%)	27% (3-45%)	Substituting urea (140 $/t) with calcium ammonium nitrate (200 $/t), ammonium sulfate ($425/t), or ammonium phosphate ($723/t) increases costs, with calcium ammonium nitrate as a represent for a 40% higher expense.	[64, 120, 121, 123-126]
**SF**	44% (30-66%)	74% (55-86%)	66% (13-80%)	20% (4-36%)	-14% (-63-22%)	Split fertilization for corn, wheat, rice, and fruits requires more labor, equipment and time costs.	
**DP**	64% (50-70%)	-	-13% (-97%-32%)	-26% (-48-(-10%))	-9% (-67-29%)	Deep placement increases machine investment and operation costs, with fertilizer applicators costing 3000 $ each. However, mechanized deep placement improves efficiency, potentially saving on	

						expensive labor.	
**L**	25% (3-37%)	-	14% (4%-25%)	81% (78-83%）	40% (20-54%)	Legume rotations reduce the demand for nitrogen input, boost yields, and enhance gross margins, effectively resulting in negative implementation costs due to their overall economic benefits.	[127, 128]
**N**	3% (-11-13%)	-	6% (5-7%)	1% (-3-5%)	-36% (-5-1%)	Adopting improved crop varieties effectively enhances nitrogen use efficiency (NUE) and stabilizes yields. However, this approach incurs costs, including necessary infrastructure investments: seed cost 250 $/ha, purchase new cultivar add 10% additional cash cost.	[129, 130]
**T**	-43% (-51-26%)	-	-13% (-44-23%)	52% (44-59%)	-1% (-9-20%)	No-till farming, diverging from traditional methods, necessitates unique skills, techniques, and equipment. However, it significantly cuts down annual fuel and labor costs by up to two-thirds compared to conventional tillage.	[131 -133]
**IR**	19% (11-28%)	-114% (-129-(-19) %)	-3% (-28-17%)	52% (44-59%)	38% (25-52%)	The implementation costs of high-efficiency irrigation systems encompass an initial investment of around $2,150 per acre (lifespan=10 years), with a typical range of $1,800 to $2,500, and an annual maintenance cost at 200 $. However, labour, pesticides and water costs will be saved.	[134 -137]
**LCP**	17% (15-23%)	-	-22% (-57-12%)	15% (10-17%)	15% (10-17%)	Implementing low crude protein (LCP) feeding in animal farms emerges as a cost-effective strategy for reducing NH3 emissions and soybean imports in China, simultaneously improving feed efficiency and potentially reducing some feed costs. Nevertheless, the requisite addition of synthetic amino acids to preserve feed nutritional quality may slightly elevate the overall cost of the feed.	[138 -142]
**DA**	10% (8-30%)	-	-14% (-34-2%)	8% (2-8%)	8% (1-8%)	Implementing dietary additives incurs initial costs related to feed and labor, yet, considering market	[143

						prices, it proves cost-effective by benefiting the environment, animal welfare, and producers alike, supported by solid scientific and practical evidence. Primarily, while the expense is tied to the additives, their use significantly lowers feed consumption, leading to an overall decrease in feed costs, demonstrating a favorable cost trend.	-145]
**BED**	-38% (-47-44%)	-	6% (-132-27%)	14% (5-22%)	14% (5-22%)	The implementation costs of suitable bedding materials include expenses for wood shavings, straw, and other bedding options, along with labor costs, which should be calculated on a per-head annual basis. These materials not only help prevent NH_3 emissions by reducing manure exposure but also provide benefits by repurposing manure as a fertilizer substitute.	[146 -149]
**F**	46% (31-59%)	-	54% (27-77%)	-	-	The costs of floor management measures to prevent NH_3 emissions include investment, labor, and operational expenses for systems like flushing, immediate manure transport, filtration, biofiltration, or bio-fermented mattress materials. These costs exclude manure removal expenses but focus on reducing manure exposure and surface area in animal housing.	[150 -153]
**RE**	-18% (-174-30%)	-	50% (19-57%)	-	-	Frequent manure removal is generally cost-effective despite initial investment costs for systems like manure conveyor belts, as it reduces labor expenses. The total costs include construction, operational maintenance, repair supplies, labor, and electricity, with construction expenses depreciated annually.	[150, 151, 154]
**AIR**	40% (23-53%)	-	-30% (-88-9%)	-	-	The costs of air cleaning or scrubbing to prevent NH_3 emissions include installation expenses,	[146,

						materials like chemical reagents or microbial absorbents, labor, and maintenance costs involving water and electricity. These systems, with a typical lifespan of 10 years.	155-158]
**TU**	-92% (-182-(-38%))	-	53% (-15-85%)	-	-	The costs of turning manure include investment in compost turners, labor for operating the machinery, and operational expenses like energy consumption.	[159]
**AE**	70% (43-78%)	47% (2-71%)	19% (1-34%)	-	28% (22-50%)	The costs of adjusting aeration modes include significant initial investments in construction and installation of aerators, electrical systems, and piping, along with labor and operational expenses like power consumption, making it less suitable for backyard farming systems.	[160, 161]
**SC**	31% (13-47%)	0.3% (-7-7%)	-7% (-60-26%)	-	72.2% (72.2-72.4%)	The costs of storage covers primarily include material expenses and labor, with covering materials generally lasting three to five years.	[162-164]
**AD**	38% (30-47%)	97% (83-100%)	29% (19-40%)	22% (18-25%)	29% (28-31%)	The costs of using acidifiers or additives primarily include material expenses, which vary significantly among different substances, averaging out overall; some materials like apple pomace incur negligible costs, while commonly used additives such as lignite, sodium bisulfate, and biochar not only incur specific costs but also enhance the nutrient content of manure, effectively substituting chemical fertilizers.	[165-169]
**CS**	-	10%	-	-	-	The costs for clinker substitution are influenced by factors such as the availability and transportation of substitute materials, modifications required in production processes, and potential changes in product performance.	[170]
**IFQ**	-	46%	-	-	-	Improving fuel quality generally involves higher	

						initial costs due to the procurement of higher-grade coal and potential modifications to existing infrastructure. However, these costs can be offset by benefits such as improved combustion efficiency, reduced maintenance expenses, and lower emissions control costs, ultimately leading to long-term economic and environmental advantages.	[171]
**EII**	-	41%<br>(19-59.9%)	-	-	-	Implementing energy intensity and efficiency improvements may involve initial costs for advanced technologies like preheater kilns and heat recovery systems.	[171, 172]
**ELE**	-	16%<br>(4-28%)	-	-	-	Electrification needs initial costs due to infrastructure upgrades and renewable energy integration.	[172]
**FS**	-	49%<br>(15-83%)	-	-	-	The process entails modifying the power plant's infrastructure to support natural gas combustion, which includes installing natural gas burners, adjusting boiler systems, and upgrading emission control technologies.<br>1.8-6.1 $/kg NO_x	[171, 173-175]
**FB**	-	30%	-	-	-	3.4 $/kg NO_x	[175]
**SC**	-	45%<br>(26-64%)	-	-	-	1.2 $/kg NO_x	[176 -178]
**PAI**	-	57%<br>(43-70%)	-	-	-	3.7 $/kg NO_x	[176, 177]
**SCR**	80%	83%<br>(29-100%)	-	-	-	2.5 (2-7.5) $/kg NO_x	[170,

							174, 175, 178-188]
**SNCR**	-	62% (29-90%)	-	-	-	Compared to SCR, energy costs are lower, and less space is required<br>1.55 $/kg NO_x	[170, 175, 178, 189]
**LNB**	-	43% (18-80%)	-	-	-	1.2 (1-4) $/kg NO_x	[174, 175, 177, 178, 190]
**CD**	-	78% (47-96%)	82% (38-100%)	-	-	The implementation costs of catalytic decomposition are largely due to the expense of noble metals and specialized catalysts like spinel and perovskite-structured metal oxides.	[188, 191-198]
**EB**	-	78% (70-90%)	-	-	-	Similar to SNCR, this process does not utilize expensive catalytic beds, thereby reducing capital costs. Nevertheless, unlike most other NO_x mitigation technologies, EB is not particularly selective and by-products (other than N_2 and H_2O) are produced in the reaction chain, such as NH_4NO_3 and $(NH_4)_2SO_4$. However, these by-products can be commercialized for other applications, such as the production of fertilizers or explosives.	[188, 199-203]
**IEM**	-	35% (30-40%)	-	-	-	Basic IEM (slide valves, 2-stroke slow speed only): 9 (€/t NOx)<br>Average IEM: 40 (€/t NO_x)	[179]
**MC**	-	26%	-	-	-	From an economic perspective, installing the	

		(17-35%)				humidifier involves high initial costs and requires large surface and volume. However, the reduced fuel and lubricating oil consumption significantly can lower the engine's operating costs.<br>2,200 $/Vehicle	[179, 204]
**WJ**	-	56% (42-70%)	-	-	-	Direct water injection: 363(€/t NO_x)	[179, 180]
**NE**	-	45% (14-100%)	-	-	-	Longer electric range; charging station<br>2900 $/Vehicle	[2, 204]
**DOC**		10% (5-15%)	-	-	-	3-8 $/kg NO_x	[174]
**DPF**		8% (4-12%)	-	-	-	10-25 $/kg NO_x	[174]
**SCRT**	30%	30% (12-48%)	-	-	-	1-3 $/kg NO_x	[174, 178]
**EGR**		30%	-	-	-	The implementation cost of exhaust gas recirculation varies but generally includes expenses for system installation and potential engine modifications.	[178]
**DR**		8%	-	-	-	300 $/Vehicle	[204]
**ES**	5% (0-10%)	20% (10-30%)	-	-	-	4.5 $/kg NO_x<br>225 $/kg NH_3	[205]
**VH**		35% (20-50%)	-	-	-	5.8 $/kg NO_x<br>290 $/kg NH_3	[206]

**HEPA**		35% (20-50%)	-	-	-	15.1 $/kg NO_x<br>600 $/kg NH_3	[207, 208]
**PAB**	5% (0-10%)	5% (0-10%)	-	-	-	-	[209]
**IAS**	-4%	-	-	62%	-	Capital Cost<br>low case / base case / high case<br>$6,110 / $13,400 / $25,000 new<br>$6,110 / $12,480 / $25,000 retrofit<br>O&M Cost<br>(low case / base case / high case)<br>$550 / $950 / $1,750	[210, 211]
**DT**	-10%	-	-	84%	-	Capital Cost<br>low case / base case / high case<br>$850 / $1,210 / $1,440 toilet<br>$2,670 / $3,200 / $4,170 tank<br>O&M Cost<br>(low case / base case / high case)<br>$130 / $170 / $220 flush<br>$195 / $280 / $360 dry	[210, 211]
**CT**	-10%	-	-	77% (69-84%)	-	Capital Cost<br>low case / base case / high case<br>$6,150 / $8,340 / $10,530<br>O&M Cost<br>(low case / base case / high case)<br>$100 / $150 / $200	[174, 210, 211]
**ISI**	-	-	-	-	61% (25-100%)	The implementation cost for improved sanitation infrastructure is considerable, reflecting the expenses for upgrading or constructing new systems to reduce sewage leakage.	[212]
**ERE**	-	-	-	70%	-	-	

							[213]
**LHWR**	15% (10-20%)	5% (0-10%)	-	15% (10-20%)	15% (10-20%)	1-5 \$/kg NO_x<br>0-1 \$/kg NH_3	[214]
**DSC**	-	-	-	85% (70-100%)	-	In 2013, the average costs entailed operating expenses of 0.2 \$/t, construction at 0.05 \$/t, wastewater operation at 0.1 \$/t, and sludge treatment at 0.03 \$/t, alongside average treatment fees of 0.1 \$/t for residential areas and 0.14 \$/t for non-residential areas.	[2]
**IW**	15% (10-20%)	5% (0-10%)	-	-	-	Waste-to-energy facilities (e.g. incineration plants with selective catalytic reduction): 5-15 \$/kg NO_x; Improved combustion processes (e.g. low-NO_x burners): 1-4 \$/kg NO_x	[215]
**RCI**			-	-	-	The cost of waste incineration in 2020 for city is about 40 \$/t.	[2]
**NPS+MBR**	-	-	-	-	82% (74-90%)	Assuming electricity costs of 0.11 \$/kWh and external carbon source costs of 350 \$/t, the total operating costs for a Membrane Bioreactor (MBR) system are estimated. Without pretreatment, the operating cost is 10 \$/m^3 of leachate, whereas with negative pressure steam-stripping pretreatment, it reduces to 1.8 \$/m^3.	[216]
**CP+MVR**	-	-	-	-	81%	The electrocoagulation combined with biological treatment costs 5.02 \$ per m^3, proving more economical than the prevalent leachate treatment in China, which stands at 8.16 \$ per m^3. This method offers a cost-effective alternative to the widely used biological nitrogen removal and membrane technology combination.	[217]
**EC+BT**	-	-	-	-	65%	EC is considered a low-cost technique with good	

						removal efficiencies for treating landfill leachate reverse osmosis concentrate. It has advantages such as being a high-tech and automated system that doesn't require chemical additions, though it does require energy input.	[218]
**UVH+BAC**	-	-	-	-	63% (36-90%)	The combination of advanced oxidation (like UV/H_2O_2) with BAC can be attractive for addressing both nitrate and organic contaminant removal. When considering UV treatment costs, factors such as UV dose, upstream treatment processes, and water quality can significantly impact energy requirements and overall costs.	[219]
**SFS**	-	-	50%	-	-	The implementation cost can be higher due to the advanced control systems and infrastructure requirements.	[220, 221]
**MF**	-	-	-	99% (97-100%)	-	Membrane filtration incurs higher operational and maintenance costs due to the frequent need for membrane replacement, stemming from their short lifespan. Despite these costs, its broad applicability and numerous advantages over other processes make it an attractive option in various industries.	[222-226]
**ACU**	-	-	63% (50-73%)	-	-	Although the copper addition itself is not extremely expensive, the process requires precise dosing and monitoring. The exact costs would vary based on specific system requirements and scale.	[227-229]
**CPR**	-	-	-	87% (58-99%)	-	Despite high chemical costs and the drawback of producing sludge that increases operating expenses, chemical precipitation is advantageous due to its quick process, low capital investment, and ability to recover ammonia for fertilizers, offering dual benefits in wastewater treatment and resource recovery.	[222, 230-241]

**IE**	-	-	-	98% (97-100%)	-	Despite the significant capital and operational costs due to chemical reagent regeneration, ion exchange remains advantageous when low-cost minerals like zeolites are used. However, its primary drawback is desorption caused by equilibrium shifts during reversible chemical reactions, particularly when ammonia concentration in the influent drops.	[222, 242-247]
**ADS**	-	-	-	63% (16-90%)	-	Despite minor drawbacks, adsorption emerges as a viable alternative to other technologies due to its simple and cost-effective setup and operation. Widely recognized as an economical and environmentally friendly process, adsorption is favored for its long-term operational stability, low maintenance requirements, and reliable performance.	[222, 224, 244, 248-256]
**BC**	-	-	-	97% (95-99%)	-	The process of breakpoint chlorination requires chlorine dosing equipment and pH control systems, which are initial investments. Ongoing operational costs include chlorine chemicals and energy for mixing and monitoring.	[257]
**BR**	-	-	-	46% (15-80%)	-	Bioreactor implementation costs vary significantly: subsurface drainage bioreactors range from under $5,000 to $27,000, with cost efficiencies between $2.50 to $48 per kg of nitrogen removed annually, while more advanced membrane bioreactors can reach $2-$3 per ton or higher.	[174, 258-262]
**SBR**	-	-	99%	-	-	The Nit/DNit SBR process requires intermittent aeration and carbon source addition equipment, but it is still considered a cost-effective nitrogen reduction strategy for ammonia-rich wastewater treatment. It can lower operational costs by around 1-1.8 $\$/m^3$ compared to traditional	[263]

						nitrification/denitrification.	
**SRT**	-	-	47% (22-72%)	-	-	Costs mainly arise from additional monitoring and control systems. It primarily involves adjusting existing operational parameters rather than installing new equipment.	[264, 265]
**BS**	-	-	67% (40-94%)	-	-	The implementation cost of a bioscrubber varies depending on factors like size and capacity. However, it generally involves moderate setup costs for the scrubber unit and microbial culture, along with operation expenses for methanol supply and maintenance.	[266, 267]
**AA**	-	-	-	86% (60-100%)	-	Biological treatment, involving aerobic/anaerobic bacteria, faces challenges such as slow bio-conversion, long start-up times, and inefficiency in treating high ammonia concentrations over 300 mg/L or wastewater with fluctuating ammonia levels and imbalanced nutrients. Additionally, the need for high aeration rates to ensure effective ammonia removal can lead to increased power consumption and operating costs.	[222, 223, 268-271]
**AWTP**	-	-	-	74% (30-98%)	-	-	[222, 272-281]

1179

Table S6. Determination criteria of the potential implementation rate for different mitigation measures

Abbr.	Determination boundary	Adoption rate under different exceedance levels (%)					Income adjustment coefficient			
		<0	0-100%	100%-200%	200%-500%	>500%	Low income	Lower-middle income	Upper-middle income	High income
EEF	all	15%	30%	50%	70%	90%	1	1	1	1
OF	all	10%	25%	35%	45%	55%	1	1	1	1
L	all	5%	10%	20%	20%	20%	0.5	0.7	0.85	1
OPR	all	35%	45%	55%	65%	80%	0.35	0.6	0.8	1
US	all	25%	35%	50%	60%	70%	0.25	0.5	0.7	1
SF	all	35%	45%	55%	65%	80%	0.35	0.6	0.8	1
DP	all	25%	35%	50%	60%	70%	0.25	0.5	0.7	1
N	all	0%	15%	20%	25%	35%	0	0.5	0.8	1
IR	all	5%	10%	15%	20%	25%	0	0.5	0.8	1
T	all	5%	10%	15%	20%	25%	0	0.5	0.8	1
LCP	all	15%	28%	38%	45%	53%	0	0.25	0.5	1
DA	all	14%	27%	41%	57%	73%	1	1	1.1	1.2
BED	all	55%	42%	35%	26%	14%	0.5	0.8	1	1.2
F	all	14%	26%	35%	42%	50%	0.5	0.8	1	1.2
RE	air	66%	47%	35%	23%	14%	0.4	0.65	0.8	1
AIR	all	23%	42%	57%	67%	75%	0	0.2	0.5	1
TU	air	42%	37%	27%	13%	0%	0.5	0.7	0.85	1
AE	all	20%	37%	49%	66%	80%	0.5	0.8	1	1.2
SC	all	10%	21%	32%	57%	70%	1	1	1	1
AD	all	10%	21%	27%	41%	57%	0.5	0.8	1	1.2
CM	air	20%	35%	45%	65%	80%	0.5	0.5	0.7	1
PI	air	50%	75%	85%	95%	100%	0.4	0.65	0.85	1
ET	air	10%	25%	45%	65%	80%	0.25	0.45	0.8	1
NE	air	10%	25%	35%	50%	70%	0.25	0.55	0.85	1
ECC	air	50%	65%	75%	85%	95%	0.45	0.65	0.85	1
DR	air	5%	10%	20%	30%	45%	0	0.3	0.5	1
HF	air	20%	35%	45%	60%	75%	0.2	0.6	0.8	1

AT	surface water	50%	65%	75%	88%	100%	0.5	0.75	0.85	1
LHWR	all	50%	65%	75%	88%	100%	0.5	0.7	0.85	1
IW	air	25%	50%	70%	85%	95%	0.2	0.55	0.8	1
LF	groundwater	40%	55%	70%	80%	95%	0.45	0.65	0.8	1
WTP	surface water	45%	55%	70%	80%	95%	0.4	0.65	0.8	1
AWTP	surface water	45%	55%	70%	80%	95%	0.4	0.65	0.8	1

*Note: The determination of the coefficients in the table was based on Gu et al. (2023) [102] and expert judgement.

Table S7. Subregion divisions, abbreviations and socioeconomic data for 31 provinces in China.

Subregion	Abbreviation	Full name	Population (10^4 person)	PGDP（10^3 Yuan）	Income level
North China (N)	BEJ	Beijing	2189.31	153.43	High-income
	TAJ	Tianjin	1386.60	194.21	High-income
	HEB	Hebei	7461.02	66.14	Lower-middle-income
	SHX	Shanxi	3491.56	61.39	Lower-middle-income
Northeast China (NE)	NEG	Inner Mongolia	2404.92	120.96	High-income
	LIA	Liaoning	4259.14	114.62	High-income
	JIL	Jilin	2407.35	104.76	High-income
	HLJ	Heilongjiang	3185.01	75.75	Upper-middle-income
East China (E)	SHH	Shanghai	2487.09	128.52	High-income
	JSU	Jiangsu	8474.80	123.82	High-income
	ZHJ	Zhejiang	6456.76	101.12	High-income
	ANH	Anhui	6102.72	59.21	Lower-middle-income
	FUJ	Fujian	4154.01	94.11	Upper-middle-income
	JXI	Jiangxi	4518.86	60.05	Lower-middle-income
	SHD	Shandong	10152.75	92.04	Upper-middle-income
South Central China (SC)	HEN	Henan	9936.55	54.73	Lower-middle-income
	HUB	Hubei	5775.26	83.22	Upper-middle-income
	HUN	Hunan	6644.49	69.76	Upper-middle-income
	GUD	Guangdong	12601.25	79.29	Upper-middle-income
	GUX	Guangxi	5012.68	53.60	Lower-middle-income
	HAN	Hainan	1008.12	57.77	Lower-middle-income
Southwest China (SW)	CHQ	Chongqing	3205.42	78.43	Upper-middle-income
	SCH	Sichuan	8367.49	60.63	Lower-middle-income
	GUI	Guizhou	3856.21	45.76	Low-income
	YUN	Yunnan	4720.93	48.79	Low-income
	TIB	Tibet	364.81	27.93	Low-income
Northwest China	SHA	Shaanxi	3952.90	89.64	Upper-middle-income
	GAS	Gansu	2501.98	46.21	Low-income

(NW)	QIH	Qinghai	592.40	73.24	Upper-middle-income
	NXA	Ningxia	720.27	73.81	Upper-middle-income
	XIN	Xinjiang	2585.23	54.58	Lower-middle-income

*Note: In this study, we categorized provincial income levels (PGDP) into four distinct gradients to assess the economic carrying capacity.

Table S8. Detailed description for damage cost of nitrogen loss ($/kg N) to air, land, and water

Species	Effect	Type	Subject	Low	Median	High
NO_x	Increased incidence of respiratory disease	human health	air/climate	12.88	23.1	38.63
	Declining visibility—loss of aesthetics	human health	air/climate	0.31	0.31	0.31
	Increased effects of airborne particulates/increased carbon sequestration in forests (includes benefits)	climate	air/climate	-11.59	-4.51	2.58
	Increased damages to buildings from acid	ecosystem	land	0.09	0.09	0.09
	Increased ozone exposure to crops	ecosystem	land	1.29	1.51	2.58
	Increased ozone exposure to forests	ecosystem	land	0.89	0.89	0.89
	Increased loss of plant biodiversity from N enrichment	ecosystem	land	2.58	7.73	12.88
NH_3	Increased incidence of respiratory disease-pm2.5	human health	air/climate	2.58	4.93	25.75
	Declining visibility—loss of aesthetics	human health	air/climate	0.31	0.31	0.31
	Increased effects of airborne particulates/increased carbon sequestration in forests (includes benefits)	human health	air/climate	-3.86	-1.93	-1.93
	Increased damages to buildings from particulates	ecosystem	land	0.09	0.09	0.09
	Increased loss of plant biodiversity	ecosystem	land	2.58	7.73	12.88
Nitrogen runoff	Declining waterfront property value	ecosystem	freshwater	0.21	0.21	0.21
	Loss of recreational use	ecosystem	freshwater	0.17	0.17	0.17
	Loss of endangered species	ecosystem	freshwater	0.01	0.01	0.01
	Increased eutrophication	ecosystem	freshwater	6.44	16.1	25.75
	Undesirable odor and taste	human health	drinking water	0.14	0.14	0.14
	Nitrate contamination	human health	drinking water	0.54	0.54	0.54

	Increased colon cancer risk	human health	drinking water	1.76	1.76	5.15
Nitrogen leaching	Undesirable odor and taste	human health	drinking water	0.14	0.14	0.14
	Nitrate contamination	human health	drinking water	0.54	0.54	0.54
	Increased colon cancer risk	human health	drinking water	1.76	1.76	5.15
coastal N loading	Loss of recreational use	ecosystem	coastal zone	6.38	6.38	6.38
	Declines in fisheries and estuarine/marine habitat	ecosystem	coastal zone	6	15.84	26

*Note: Figures in this table are the original values applicable to the EU and US in the 2010s. Negative values indicate an economic benefit. This study estimates regional mitigation benefits by applying the peer-reviewed damage cost estimates (in \$ kg^{-1} N_r) with regional PPP and WTP adjustment.

Table S9. Adjusted avoided damage cost for 31 provinces

Province	WTP (US$2020)	Ecosystem benefit ($/kg N)			Health benefit ($/kg N)				Climate benefit ($/kg N)	
		NH_3	NO_x	runoff	NH_3	NO_x	runoff	leaching	NH_3	NO_x
Beijing	24.62	11.48	5.26	24.74	23.87	11.30	12.20	5.24	-1.27	-1.13
Tianjin	11.34	5.90	3.06	24.74	26.30	12.45	12.20	18.30	-1.27	-1.13
Hebei	14.41	22.35	2.43	24.74	17.02	8.05	12.20	8.41	-1.27	-1.13
Shanxi	18.34	11.70	3.19	24.74	14.72	6.97	12.20	4.34	-1.27	-1.13
Inner Mongolia	22.27	10.30	0.86	24.74	11.44	5.42	12.20	4.01	-1.27	-1.13
Liaoning	31.00	5.96	2.47	24.74	15.16	7.17	6.57	6.46	-1.27	-1.13
Jilin	18.34	13.23	1.63	24.74	12.48	5.91	3.58	2.87	-1.27	-1.13
Heilongjiang	14.95	12.08	0.96	14.56	11.23	5.32	2.15	1.82	-1.27	-1.13
Shanghai	34.84	0.00	3.68	24.74	17.46	8.26	12.20	18.30	-1.27	-1.13
Jiangsu	5.81	0.00	4.93	24.74	16.28	7.70	6.86	8.40	-1.27	-1.13
Zhejiang	22.48	8.02	3.20	14.82	13.26	6.27	2.19	7.23	-1.27	-1.13
Anhui	16.87	6.33	3.68	16.17	13.55	6.41	2.39	3.45	-1.27	-1.13
Fujian	2.00	9.20	2.30	15.18	8.63	4.09	2.25	2.56	-1.27	-1.13
Jiangxi	39.11	6.14	2.22	8.73	10.99	5.20	1.29	1.30	-1.27	-1.13
Shandong	12.96	0.00	3.46	24.74	16.73	7.92	12.20	5.33	-1.27	-1.13
Henan	15.79	7.22	3.34	24.74	16.45	7.78	12.20	8.69	-1.27	-1.13
Hubei	28.00	8.23	2.98	11.43	14.01	6.63	1.69	2.44	-1.27	-1.13
Hunan	23.65	7.60	2.91	10.26	11.70	5.54	1.52	1.72	-1.27	-1.13
Guangdong	30.31	9.10	2.95	24.74	11.22	5.31	2.86	3.71	-1.27	-1.13
Guangxi	14.41	4.86	1.53	11.64	8.52	4.03	1.72	1.91	-1.27	-1.13
Hainan	15.79	6.14	0.74	15.74	4.43	2.10	2.33	1.41	-1.27	-1.13
Chongqing	28.00	8.31	1.86	10.27	11.66	5.52	1.52	3.35	-1.27	-1.13
Sichuan	31.00	10.56	1.12	8.79	10.34	4.89	1.30	1.65	-1.27	-1.13
Guizhou	14.41	4.80	1.56	6.63	5.54	2.62	0.98	1.34	-1.27	-1.13

Yunnan	61.24	10.69	1.31	11.38	5.03	2.38	1.68	1.10	-1.27	-1.13
Tibet	50.61	5.41	0.63	1.88	4.10	1.94	0.28	0.35	-1.27	-1.13
Shaanxi	13.33	10.84	1.85	24.74	14.29	6.76	5.35	3.80	-1.27	-1.13
Gansu	18.34	16.61	0.84	24.74	7.46	3.53	3.80	2.21	-1.27	-1.13
Qinghai	18.34	4.68	0.63	7.12	7.43	3.52	1.05	0.52	-1.27	-1.13
Ningxia	18.34	16.98	1.06	24.74	9.40	4.45	12.20	9.23	-1.27	-1.13
Xinjiang	17.97	4.14	0.69	24.74	12.85	6.08	4.08	1.82	-1.27	-1.13

*Note: WTP for each province was derived from Xu et. al, 2023 [282].

Table S10. The exceedance for regional nitrogen boundaries and marginal benefit adjustment coefficient at provincial level in China

Province	Exceeding multiple				Marginal benefit adjustment coefficient		
	air	surface water	groundwater	all	atmosphere	runoff	leaching
Beijing	0.64	13.69	2.70	1.69	1.34	2.54	1.12
Tianjin	1.29	25.82	7.39	2.59	1.87	4.29	1.96
Hebei	0.69	8.54	2.96	1.25	1.38	1.56	1.14
Shanxi	0.27	3.90	0.47	0.50	1.04	0.80	0.90
Inner Mongolia	-0.22	1.41	-0.55	-0.21	0.64	0.63	0.64
Liaoning	0.38	4.02	2.05	0.81	1.13	0.86	1.06
Jilin	-0.06	1.16	1.86	0.23	0.77	0.62	0.99
Heilongjiang	-0.20	1.09	0.50	0.03	0.65	0.62	0.90
Shanghai	0.69	10.63	9.53	1.93	1.38	2.10	2.56
Jiangsu	0.66	7.40	3.86	1.47	1.35	1.28	1.22
Zhejiang	0.49	0.70	1.94	0.71	1.22	0.59	0.99
Anhui	0.50	1.49	1.86	0.79	1.22	0.63	0.99
Fujian	0.43	0.09	0.44	0.31	1.17	0.58	0.90
Jiangxi	0.13	-0.13	-0.01	0.02	0.93	0.54	0.87
Shandong	0.92	13.68	6.06	1.74	1.57	2.54	1.77
Henan	1.14	7.36	4.45	1.86	1.75	1.28	1.37
Hubei	0.31	1.01	0.39	0.48	1.07	0.61	0.90
Hunan	0.48	0.04	0.37	0.30	1.21	0.58	0.90
Guangdong	0.45	1.06	1.67	0.73	1.19	0.62	0.98
Guangxi	0.05	-0.12	0.31	0.01	0.86	0.54	0.89
Hainan	0.00	0.94	0.66	0.24	0.82	0.60	0.91
Chongqing	0.32	0.35	1.74	0.44	1.08	0.59	0.98
Sichuan	0.10	-0.28	-0.04	-0.06	0.90	0.50	0.86
Guizhou	0.04	-0.36	0.09	-0.11	0.85	0.47	0.88
Yunnan	-0.39	-0.41	-0.54	-0.42	0.50	0.46	0.64
Tibet	-0.72	-0.89	-0.94	-0.87	0.23	0.32	0.47
Shaanxi	0.07	0.64	0.10	0.16	0.87	0.59	0.88
Gansu	-0.32	0.34	-0.61	-0.35	0.55	0.58	0.61

Qinghai	-0.51	-0.64	-0.87	-0.68	0.40	0.39	0.50
Ningxia	-0.25	6.55	-0.40	-0.16	0.61	1.20	0.70
Xinjiang	-0.45	0.10	-0.78	-0.54	0.45	0.58	0.54

Table S11. Activity data (A) and main parameters (P) with their coefficient of variation (CV) used for calculating N budgets

A/P	Item	Unit	CV (%)	Ref
For cropland				
A	Fertilizer use	kg yr^{-1}	10	[91, 283-286]
A	Planting area	10^3 ha	10	[91, 283-286]
A	Crop yield	10^7 kg yr^{-1}	10	[91, 283-286]
A	Irrigation water volume	10^8 m^3	20	[85, 283]
P	Crop N content (grain)	%	20	[85, 285, 287, 288]
P	Crop N content (straw)	%	20	[85, 285, 287, 288]
P	Harvest Index	-	50	[86, 91]
P	N deposition density	kg N ha^{-1} yr^{-1}	50	[289-293]
P	N fixation rate	kg N ha^{-1} yr^{-1}	25	[58, 86]
P	Crop & straw fate	%	50	[91, 294]
P	N_r emission factors	%	50	[85, 86]
For livestock				
A	Livestock production	head yr^{-1}	10	[85, 116, 285, 295, 296]
P	N content in animal products	%	20	[86, 288]
P	Excretion index	kg N $head^{-1}$ yr^{-1}	10	[85, 295, 296]
P	Manure N content	%	25	[85, 91, 285, 296]
P	Fate of livestock excretion	%	30	[86, 91, 116]
P	NH_3 emission factor	kg N $head^{-1}$ yr^{-1}	50	[85, 86, 116, 296]
For aquaculture				
A	Fishery production	kg yr^{-1}	10	[283]
P	Ratio of fertilizer to feed	%	20	[297]
P	N content of aquaculture products	%	20	[86, 288]
P	N_r emission factors	%	50	[85, 91, 298-300]

For grassland				
A	Grassland area	10^4 ha	5	[85, 283, 285]
A	Grassland affected by fires	10^4 ha	10	[85, 283, 285]
P	Grass N content	%	10	[91, 301]
P	BNF rate	kg N ha^{-1} yr^{-1}	25	[86, 91]
P	N_r emission factors	%	50	[86, 91]
For forest				
A	Forest area	10^4 ha	10	[283, 302]
A	Timber harvested	10^4 m^3	10	[283]
A	Forest area affected by fires	10^3 ha	50	[91, 285]
P	Wood N content	%	15	[86]
P	Forest fire emission factors	g kg^{-1}	10	[91, 303]
P	N_r emission factors	%	50	[91, 304]
For urban green-land				
A	Area of urban green land	ha	10	[283]
A	Park area	ha	10	[283]
P	N_r emission factors	%	50	[91, 303]
P	BNF rate	kg N ha^{-1} yr^{-1}	15	[283]
For human				
A	Population	10^4 person	5	[91, 305]
A	Urbanization rate	%	5	[91, 283]
P	Food intake	kg N $capita^{-1}$ yr^{-1}	10	[295]
P	Food waste ratio	%	20	[86, 91, 306]
P	Human excretion fate	%	30	[86, 91]
P	N_r emission factors	%	50	[86, 91]
For pet				
A	Number of pets	head	10	[283]
P	Pet food intake	kg $capita^{-1}$ d^{-1}	30	[86, 307]
P	Pet excretion fate	%	30	[86, 307]
For garbage				
A	Garbage clearance volume	10^7 kg yr^{-1}	10	[283]
A	Garbage harmlessly treated	10^7 kg yr^{-1}	10	[283]

A	Garbage produced	10^7 kg yr^{-1}	10	[283]
A	Industrial garbage production	10^7 kg yr^{-1}	10	[283]
P	Garbage N fate	%	20	[211]
P	Composition of municipal domestic waste	%	20	[308]
P	N_r emission factors	kg N kg^{-1}	50	[86, 91]
For wastewater				
A	Sewage discharge	10^4 m^3 yr^{-1}	10	[283]
A	Sludge production	10^7 kg yr^{-1}	10	[283]
P	Wastewater N content	mg N L^{-1}	15	[309, 310]
P	N_r emission factors	%	50	[86, 91, 311, 312]
For industry				
A	Industrial products	10^7 kg yr^{-1}	10	[283]
A	Haber-Bosch N fixation	Tg N yr^{-1}	10	[283]
A	NO_x emission	Tg N yr^{-1}	10	[283]
P	N content for industrial products	%	15	[313]
P	N_r emission factors	%	50	[84, 86]
For traffic				
A	Number of vehicles	10^4	5	[283]
A	NO_x emission	Tg N yr^{-1}	10	[283]
P	N_r emission factors	%	50	[85, 86]

Table S12. Uncertainty analysis

Subsystem	Unit	Calculated value	Estimated range	Normalized range (%)	Uncertainty contribution (%)
N_r losses in 2020					
cropland	(Tg N yr^{-1})	10.9	8.9-12.8	18%	39%
livestock	(Tg N yr^{-1})	3.4	2.6-4.1	24%	16%
grassland	(Tg N yr^{-1})	1.4	1.0-1.7	27%	7%
forest	(Tg N yr^{-1})	1.1	0.7-1.4	33%	7%
urban green-land	(Tg N yr^{-1})	0.2	0.2-0.3	23%	1%
aquaculture	(Tg N yr^{-1})	0.7	0.6-0.9	18%	3%
industry	(Tg N yr^{-1})	1.6	1.5-1.6	4%	1%
traffic	(Tg N yr^{-1})	2.0	2.0-2.1	4%	2%
human	(Tg N yr^{-1})	2.2	1.6-2.7	26%	11%
garbage	(Tg N yr^{-1})	0.6	0.5-0.8	22%	3%
WTP	(Tg N yr^{-1})	2.6	2.1-3.1	18%	9%
N_r losses under CSM					
cropland	(Tg N yr^{-1})	5.1	3.2-8.6	53%	43%
livestock	(Tg N yr^{-1})	2.0	1.0-3.9	73%	23%
grassland	(Tg N yr^{-1})	1.2	0.8-1.5	27%	5%
forest	(Tg N yr^{-1})	0.6	0.4-0.8	33%	3%
urban green-land	(Tg N yr^{-1})	0.2	0.1-0.2	23%	1%
aquaculture	(Tg N yr^{-1})	0.4	0.3-0.5	32%	2%
industry	(Tg N yr^{-1})	0.9	0.8-1.0	13%	2%
traffic	(Tg N yr^{-1})	1.3	1.2-1.6	15%	3%
human	(Tg N yr^{-1})	1.1	0.5-1.8	60%	11%
garbage	(Tg N yr^{-1})	0.5	0.3-0.6	29%	2%
WTP	(Tg N yr^{-1})	1.3	1.0-1.6	23%	5%

REFERENCES

1. Gu B, Ju X, Chang J *et al*. Integrated reactive nitrogen budgets and future trends in China. *Proc Natl Acad Sci* 2015; **112**: 8792-7.

2. Tian Y, Hu Y, Su M *et al*. Mitigation measures could aggravate unbalanced nitrogen and phosphorus emissions from land-use activities. *Environ Sci Technol* 2024; **58**: 4627-36.

3. Chen Y, Xu C, Ge Y *et al*. A 100-m gridded population dataset of China's seventh census using ensemble learning and geospatial big data. *Earth Syst Sci Data Discuss* 2024; **2024**: 1-19.

4. Zhao N, Liu Y, Cao G *et al*. Forecasting China's GDP at the pixel level using nighttime lights time series and population images. *GIsci Remote Sens* 2017; **54**: 407-25.

5. Zhu Z, Zhang X, Dong H *et al*. Integrated livestock sector nitrogen pollution abatement measures could generate net benefits for human and ecosystem health in China. *Nat Food* 2022; **3**: 161-8.

6. Gao Q, Zhang X, Liu L *et al*. A database of atmospheric inorganic nitrogen deposition fluxes in China from satellite monitoring. *Sci Data* 2023; **10**: 698.

7. Jia Y, Wang Q, Zhu J *et al*. A spatial and temporal dataset of atmospheric inorganic nitrogen dry deposition in China (2006-2015). *Science Data Bank*; 2019.

8. Jia Y, Wang Q, Zhu J *et al*. A spatial and temporal dataset of atmospheric inorganic nitrogen wet deposition in China (1996-2015). *Science Data Bank*; 2018.

9. Galloway JN, Townsend AR, Erisman JW *et al*. Transformation of the nitrogen cycle: Recent trends, questions, and potential solutions. *Science* 2008; **320**: 889-92.

10. He C, Wang X, Liu X *et al*. Nitrogen deposition and its contribution to nutrient inputs to intensively managed agricultural ecosystems. *Ecol Appl* 2010; **20**: 80-90.

11. Liu X, Duan L, Mo J *et al*. Nitrogen deposition and its ecological impact in China: An overview. *Environ Pollut* 2011; **159**: 2251-64.

12. Ma R, Li K, Guo Y *et al*. Mitigation potential of global ammonia emissions and related health impacts in the trade network. *Nat Commun* 2021; **12**: 6308.

13. Gu B, Zhang L, Van Dingenen R *et al*. Abating ammonia is more cost-effective than nitrogen oxides for mitigating $PM_{2.5}$ air pollution. *Science* 2021; **374**: 758-62.

14. Rockström J, Gupta J, Qin D *et al*. Safe and just Earth system boundaries. *Nature* 2023; **619**: 102-11.

15. Ministry of Ecology and Environment of the People's Republic of China. Ambient air quality standards (GB 3095—2012); 2012.

16. Lafortune G, Fuller G, Kloke-Lesch A *et al*. *Europe's Future and the SDGs: Europe Sustainable Development Report 2023/24*: Dublin University Press, 2024.

17. Bai Z, Fan X, Jin X *et al*. Relocate 10 billion livestock to reduce harmful nitrogen pollution exposure for 90% of China's population. *Nat Food* 2022; **3**: 152-60.

18. Zhao H, Fan X, Bai Z *et al*. Holistic food system innovation strategies can close up to 80% of China's domestic protein gaps while reducing global environmental impacts. *Nat*

Food 2024; **5**: 581-91.
19. Zhang C, Guo H, Huang H *et al*. Atmospheric nitrogen deposition and its responses to anthropogenic emissions in a global hotspot region. *Atmos Res* 2021; **248**: 105137.
20. Xu W, Zhao Y, Liu X *et al*. Atmospheric nitrogen deposition in the Yangtze River basin: Spatial pattern and source attribution. *Environ Pollut* 2018; **232**: 546-55.
21. Deng O, Huang S, Wang C *et al*. Atmospheric nitrogen pollution control benefits the coastal environment. *Environ Sci Technol* 2024; **58**: 449-58.
22. Ministry of Ecology and Environment of the People's Republic of China. Environmental quality standards for surface water (GB 3838-2002); 2002.
23. Camargo JA, Alonso Á. Ecological and toxicological effects of inorganic nitrogen pollution in aquatic ecosystems: A global assessment. *Environ Int* 2006; **32**: 831-49.
24. de Vries W, Kros J, Kroeze C *et al*. Assessing planetary and regional nitrogen boundaries related to food security and adverse environmental impacts. *Curr Opin Environ Sustain* 2013; **5**: 392-402.
25. Ma T, Sun S, Fu G *et al*. Pollution exacerbates China's water scarcity and its regional inequality. *Nat Commun* 2020; **11**: 650.
26. Schulte-Uebbing LF, Beusen AHW, Bouwman AF *et al*. From planetary to regional boundaries for agricultural nitrogen pollution. *Nature* 2022; **610**: 507-12.
27. Lofton DD, Hershey AE, Whalen SC. Evaluation of denitrification in an urban stream receiving wastewater effluent. *Biogeochemistry* 2007; **86**: 77-90.
28. Zhao Y, Xia Y, Ti C *et al*. Nitrogen removal capacity of the river network in a high nitrogen loading region. *Environ Sci Technol* 2015; **49**: 1427-35.
29. Wang M, Houlton BZ, Wang S *et al*. Human-caused increases in reactive nitrogen burial in sediment of global lakes. *Innovation (N Y)* 2021; **2**: 100158.
30. Haddeland I, Lettenmaier DP, Skaugen T. Effects of irrigation on the water and energy balances of the Colorado and Mekong river basins. *J Hydrol (Amst)* 2006; **324**: 210-23.
31. Nitrate and nitrite in drinking-water: Background document for development of WHO Guidelines for Drinking-water Quality: World Health Organization; 2003.
32. European Commission. Council Directive of 12 December 1991 Concerning the Protection of Waters against Pollution Caused by Nitrates From Agricultural Sources (91/676/EEC). Brussels: European Commission; 1991.
33. National Bureau of Disease Control and Prevention. Standards for Drinking Water for Quality (GB5749-2022); 2022.
34. de Vries W, Schulte-Uebbing L, Kros H *et al*. Spatially explicit boundaries for agricultural nitrogen inputs in the European Union to meet air and water quality targets. *Sci Total Environ* 2021; **786**: 147283.
35. Ehhalt D, Prather M, Dentener F *et al*. Atmospheric chemistry and greenhouse gases. *Climate change 2001: the scientific basis, Intergovernmental panel on climate change* 2001.
36. Gong C, Tian H, Liao H *et al*. Global net climate effects of anthropogenic reactive nitrogen. *Nature* 2024.

37. Bai Z, Fan X, Jin X *et al*. Relocate 10 billion livestock to reduce harmful nitrogen pollution exposure for 90% of China's population. *Nat Food* 2022; **3**: 152-60.
38. Zhao H, Fan X, Bai Z *et al*. Holistic food system innovation strategies can close up to 80% of China's domestic protein gaps while reducing global environmental impacts. *Nat Food* 2024; **5**: 581-91.
39. Gu B, Sutton MA, Chang SX *et al*. Agricultural ammonia emissions contribute to China's urban air pollution. *Front Ecol Environ* 2014; **12**: 265-6.
40. Fu H, Zhang Y, Liao C *et al*. Investigating $PM_{2.5}$ responses to other air pollutants and meteorological factors across multiple temporal scales. *Sci Rep* 2020; **10**: 15639.
41. Gu B, Zhang L, Van Dingenen R *et al*. Abating ammonia is more cost-effective than nitrogen oxides for mitigating $PM_{2.5}$ air pollution. *Science* 2021; **374**: 758-62.
42. Xu W, Zhao Y, Wen Z *et al*. Increasing importance of ammonia emission abatement in PM2.5 pollution control. *Sci Bull (Beijing)* 2022; **67**: 1745-9.
43. Guo Y, Zhang L, Winiwarter W *et al*. Ambitious nitrogen abatement is required to mitigate future global $PM_{2.5}$ air pollution toward the World Health Organization targets. *One Earth* 2024; **7**: 1600-13.
44. Grell GA, Peckham SE, Schmitz R *et al*. Fully coupled "online" chemistry within the WRF model. *Atmos Environ (1994)* 2005; **39**: 6957-75.
45. Buchholz R, Emmons L, Tilmes S *et al*. CESM2.1/CAM-chem instantaneous output for boundary conditions. *UCAR/NCAR-Atmospheric chemistry observations and modeling Laboratory* 2019; **10**.
46. Emmons LK, Schwantes RH, Orlando JJ *et al*. The chemistry mechanism in the community Earth system model Version 2 (CESM2). *J Adv Model Earth Syst* 2020; **12**.
47. Zaveri RA, Peters LK. A new lumped structure photochemical mechanism for large‐scale applications. *J Geophys Res-Atmos* 1999; **104**: 30387-415.
48. Zaveri RA, Easter RC, Fast JD *et al*. Model for simulating aerosol interactions and chemistry (MOSAIC). *J Geophys Res-Atmos* 2008; **113**.
49. Chen D, Liu Z, Fast J *et al*. Simulations of sulfate–nitrate–ammonium (SNA) aerosols during the extreme haze events over northern China in October 2014. *Atmos Chem Phys* 2016; **16**: 10707-24.
50. Wiedinmyer C, Akagi SK, Yokelson RJ *et al*. The Fire INventory from NCAR (FINN): a high resolution global model to estimate the emissions from open burning. *Geosci Model Dev* 2011; **4**: 625-41.
51. Guenther A, Karl T, Harley P *et al*. Estimates of global terrestrial isoprene emissions using MEGAN (Model of Emissions of Gases and Aerosols from Nature). *Atmos Chem Phys* 2006; **6**: 3181-210.
52. Ginoux P, Chin M, Tegen I *et al*. Sources and distributions of dust aerosols simulated with the GOCART model. *J Geophys Res-Atmos* 2001; **106**: 20255-73.
53. Li M, Zhang Q, Kurokawa J *et al*. MIX: a mosaic Asian anthropogenic emission inventory under the international collaboration framework of the MICS-Asia and HTAP. *Atmos Chem*

Phys 2017; **17**: 935-63.

54. Lafortune G, Fuller G, Kloke-Lesch A *et al*. *Europe's Future and the SDGs: Europe Sustainable Development Report 2023/24*: Dublin University Press, 2024.
55. Wang S, Zhang X, Wang C *et al*. A high-resolution map of reactive nitrogen inputs to China. *Sci Data* 2020; **7**.
56. Wang C, Cheng K, Ren C *et al*. An empirical model to estimate ammonia emission from cropland fertilization in China. *Environ Pollut* 2021; **288**: 117982.
57. Stevens CJ, Quinton JN. Diffuse pollution swapping in arable agricultural systems. *Crit Rev Environ Sci Technol* 2009; **39**: 478-520.
58. Zhang X, Davidson EA, Mauzerall DL *et al*. Managing nitrogen for sustainable development. *Nature* 2015; **528**: 51-9.
59. Page MJ, Mckenzie JE, Bossuyt PM *et al*. The PRISMA 2020 statement: an updated guideline for reporting systematic reviews. *Brit Med J* 2021; **372**: n71.
60. WebPlotDigitizer.: WebPlotDigitizer 5.2; 2024.
61. Hedges LV, Gurevitch J, Curtis PS. The meta‐analysis of response ratios in experimental ecology. *Ecology* 1999; **80**: 1150-6.
62. Yang M, Fang Y, Sun D *et al*. Efficiency of two nitrification inhibitors (dicyandiamide and 3, 4-dimethypyrazole phosphate) on soil nitrogen transformations and plant productivity: a meta-analysis. *Sci Rep* 2016; **6**.
63. Pittelkow CM, Liang X, Linquist BA *et al*. Productivity limits and potentials of the principles of conservation agriculture. *Nature* 2015; **517**: 365-8.
64. Xia L, Lam SK, Chen D *et al*. Can knowledge‐based N management produce more staple grain with lower greenhouse gas emission and reactive nitrogen pollution? A meta‐analysis. *Glob Chang Biol* 2017; **23**: 1917-25.
65. Rosenberg MS. MetaWin 3: open-source software for meta-analysis. *Front Bioinform* 2024; **4**: 1305969.
66. Adams DC, Gurevitch J, Rosenberg MS. Resampling Tests for Meta-Analysis of Ecological Data. *Ecology* 1997; **78**: 1277.
67. Zhao Y, Xi M, Zhang Q *et al*. Decline in bulk deposition of air pollutants in China lags behind reductions in emissions. *Nat Geosci* 2022; **15**: 190-5.
68. Zhang X, Gu B, van Grinsven H *et al*. Societal benefits of halving agricultural ammonia emissions in China far exceed the abatement costs. *Nat Commun* 2020; **11**: 4357.
69. Klimont Z, Winiwarter W. Integrated ammonia abatement - Modelling of emission control potentials and costs in GAINS: IIASA, Laxenburg, Austria: IR-11-027; 2011 2011-1-1.
70. Leclère D, Obersteiner M, Barrett M *et al*. Bending the curve of terrestrial biodiversity needs an integrated strategy. *Nature* 2020; **585**: 551-6.
71. Ohashi H, Hasegawa T, Hirata A *et al*. Biodiversity can benefit from climate stabilization despite adverse side effects of land-based mitigation. *Nat Commun* 2019; **10**: 5240.
72. van Grinsven HJM, van Dam JD, Lesschen JP *et al*. Reducing external costs of nitrogen pollution by relocation of pig production between regions in the European Union. *Reg Envir*

Chang 2018; **18**: 2403-15.

73. Sobota DJ, Compton JE, Mccrackin ML *et al*. Cost of reactive nitrogen release from human activities to the environment in the United States. *Environ Res Lett* 2015; **10**: 25006.

74. Jones L, Provins A, Holland M *et al*. A review and application of the evidence for nitrogen impacts on ecosystem services. *Ecosyst Serv* 2014; **7**: 76-88.

75. Sutton MA, Howard CM, Erisman JW *et al*. *The European nitrogen assessment: sources, effects and policy perspectives*: Cambridge university press, 2011.

76. Compton JE, Harrison JA, Dennis RL *et al*. Ecosystem services altered by human changes in the nitrogen cycle: a new perspective for US decision making. *Ecol Lett* 2011; **14**: 804-15.

77. Benayas JMR, Newton AC, Diaz A *et al*. Enhancement of biodiversity and ecosystem services by ecological restoration: A meta-analysis. *Science* 2009; **325**: 1121-4.

78. Xu X, Zhang X, Zou Y *et al*. Integrated carbon and nitrogen management for cost-effective environmental policies in China. *Science* 2025; **388**: 1098-103.

79. Pinder RW, Bettez ND, Bonan GB *et al*. Impacts of human alteration of the nitrogen cycle in the US on radiative forcing. *Biogeochemistry* 2013; **114**: 25-40.

80. Ndrc. *China Agricultural Products Cost-Benefit Yearbook (2000-2018)*, 2019.

81. Gu B, Zhang X, Lam SK *et al*. Cost-effective mitigation of nitrogen pollution from global croplands. *Nature* 2023; **613**: 77-84.

82. Khan I, Lei H, Ali G *et al*. Public attitudes, preferences and willingness to pay for river ecosystem services. *International Journal of Environmental Research and Public Health* 2019; **16**: 3707.

83. Gu B, Lam SK, Reis S *et al*. Toward a Generic Analytical Framework for Sustainable Nitrogen Management: Application for China. *Environ Sci Technol* 2019; **53**: 1109-18.

84. Gu B, Ge Y, Ren Y *et al*. Atmospheric reactive nitrogen in China: sources, recent trends, and damage costs. *Environ Sci Technol* 2012; **46**: 9420-7.

85. Zhang X, Wu Y, Liu X *et al*. Ammonia emissions may be substantially underestimated in China. *Environ Sci Technol* 2017; **51**: 12089-96.

86. Gu B, Ju X, Chang J *et al*. Integrated reactive nitrogen budgets and future trends in China. *Proc Natl Acad Sci* 2015; **112**: 8792-7.

87. Cui S, Shi Y, Groffman PM *et al*. Centennial-scale analysis of the creation and fate of reactive nitrogen in China (1910–2010). *Proc Natl Acad Sci* 2013; **110**: 2052-7.

88. Zhang C, Guo H, Huang H *et al*. Atmospheric nitrogen deposition and its responses to anthropogenic emissions in a global hotspot region. *Atmos Res* 2021; **248**: 105137.

89. Zhao Y, Xia Y, Ti C *et al*. Nitrogen removal capacity of the river network in a high nitrogen loading region. *Environ Sci Technol* 2015; **49**: 1427-35.

90. Lofton DD, Hershey AE, Whalen SC. Evaluation of denitrification in an urban stream receiving wastewater effluent. *Biogeochemistry* 2007; **86**: 77-90.

91. Zhang X, Ren C, Gu B *et al*. Uncertainty of nitrogen budget in China. *Environ Pollut* 2021; **286**: 117216.

92. Camargo JA, Alonso Á. Ecological and toxicological effects of inorganic nitrogen

pollution in aquatic ecosystems: A global assessment. *Environ Int* 2006; **32**: 831-49.

93. Poikane S, Kelly MG, Salas Herrero F *et al*. Nutrient criteria for surface waters under the European Water Framework Directive: Current state-of-the-art, challenges and future outlook. *Sci Total Environ* 2019; **695**: 133888.

94. de Vries W, Kros J, Kroeze C *et al*. Assessing planetary and regional nitrogen boundaries related to food security and adverse environmental impacts. *Curr Opin Environ Sustain* 2013; **5**: 392-402.

95. Bernhardt ES. Cleaner lakes are dirtier lakes. *Science* 2013; **342**: 205-6.

96. Xu H, Mccarthy MJ, Paerl HW *et al*. Contributions of external nutrient loading and internal cycling to cyanobacterial bloom dynamics in Lake Taihu, China: Implications for nutrient management. *Limnol Oceanogr* 2021; **66**: 1492-509.

97. Qin B, Zhang Y, Zhu G *et al*. Are nitrogen-to-phosphorus ratios of Chinese lakes actually increasing? *Proc Natl Acad Sci* 2020; **117**: 21000-2.

98. Basu NB, Van Meter KJ, Byrnes DK *et al*. Managing nitrogen legacies to accelerate water quality improvement. *Nat Geosci* 2022; **15**: 97-105.

99. Gao Y, Tian Y, Zhan W *et al*. Characterizing legacy nitrogen-induced time lags in riverine nitrogen reduction for the Songhuajiang River Basin: Source analysis, spatio-seasonal patterns, and impacts on future water quality improvement. *Water Res* 2023; **242**: 120292.

100. Liu X, Beusen AHW, van Grinsven HJM *et al*. Impact of groundwater nitrogen legacy on water quality. *Nat Sustain* 2024; **7**: 891-900.

101. Zhang X, Gu B, van Grinsven H *et al*. Societal benefits of halving agricultural ammonia emissions in China far exceed the abatement costs. *Nat Commun* 2020; **11**: 4357.

102. Gu B, Zhang X, Lam SK *et al*. Cost-effective mitigation of nitrogen pollution from global croplands. *Nature* 2023; **613**: 77-84.

103. Yu C, Huang X, Chen H *et al*. Managing nitrogen to restore water quality in China. *Nature* 2019; **567**: 516-20.

104. Schulte-Uebbing LF, Beusen AHW, Bouwman AF *et al*. From planetary to regional boundaries for agricultural nitrogen pollution. *Nature* 2022; **610**: 507-12.

105. Zhao Y, Li X, Han S *et al*. Soil enzyme activities under two forest types as affected by different levels of nitrogen deposition. *Ying Yong Sheng Tai Xue Bao* 2008; **19**: 2769-73.

106. Zhao Y, Han S, Li X *et al*. Effect of simulated nitrogen deposition on soil microbial biomass. *Journal of North-East Forestry University* 2009; **37**: 49-51.

107. Lin YLMT, Duan LLTE, Yang Y *et al*. Contribution of simulated nitrogen deposition to forest soil acidification in area with high sulfur deposition. *Huanjing Kexue* 2007; **28**: 640-6.

108. Bobbink R, Hicks K, Galloway J *et al*. Global assessment of nitrogen deposition effects on terrestrial plant diversity: a synthesis. *Ecol Appl* 2010; **20**: 30-59.

109. Liu X, Duan L, Mo J *et al*. Nitrogen deposition and its ecological impact in China: An overview. *Environ Pollut* 2011; **159**: 2251-64.

110. Song X, Hu T, Xian J *et al*. Responses of litter decomposition and nutrient release to simulated nitrogen deposition in an evergreen broad-leaved forest in southwestern Sichuan.

Yingyong Shengtai Xuebao 2007; **18**: 2167-72.

111. Song X, Hu T, Xian J *et al*. Soil respiration and its response to simulated nitrogen deposition in evergreen broad-leaved forest, southwest Sichuan. *J Soil Water Conserv* 2007; **21**: 168-72.

112. Song X, Hu T, Xian J *et al*. Soil enzyme activities and its response to simulated nitrogen deposition in an evergreen broad-leaved forest, southern Sichuan. *Acta Ecol Sin* 2009; **29**: 1234-40.

113. Duan L, Xie S, Zhou Z *et al*. Calculation and mapping of critical loads for S, N and acidity in China. *Water Air Soil Pollut* 2001; **131**: 1199-204.

114. Xu W, Zhao Y, Liu X *et al*. Atmospheric nitrogen deposition in the Yangtze River basin: Spatial pattern and source attribution. *Environ Pollut* 2018; **232**: 546-55.

115. Wang C, Cheng K, Ren C *et al*. An empirical model to estimate ammonia emission from cropland fertilization in China. *Environ Pollut* 2021; **288**: 117982.

116. Zhu Z, Zhang X, Dong H *et al*. Integrated livestock sector nitrogen pollution abatement measures could generate net benefits for human and ecosystem health in China. *Nat Food* 2022; **3**: 161-8.

117. Mariano E, de Sant Ana Filho CR, Bortoletto-Santos R *et al*. Ammonia losses following surface application of enhanced-efficiency nitrogen fertilizers and urea. *Atmos Environ (1994)* 2019; **203**: 242-51.

118. Gil-Ortiz R, Naranjo MÁ, Ruiz-Navarro A *et al*. New eco-friendly polymeric-coated urea fertilizers enhanced crop yield in wheat. *Agronomy* 2020; **10**: 438.

119. Chen D, Suter H, Islam A *et al*. Prospects of improving efficiency of fertiliser nitrogen in Australian agriculture: a review of enhanced efficiency fertilisers. *Soil Res* 2008; **46**: 289-301.

120. Zhang M, Yao Y, Zhao M *et al*. Integration of urea deep placement and organic addition for improving yield and soil properties and decreasing N loss in paddy field. *Agric Ecosyst Environ* 2017; **247**: 236-45.

121. Wang W, Koslowski F, Nayak DR *et al*. Greenhouse gas mitigation in Chinese agriculture: Distinguishing technical and economic potentials. *Glob Environ Change* 2014; **26**: 53-62.

122. Bai M, Impraim R, Coates T *et al*. Lignite effects on NH_3, N_2O, CO_2 and CH_4 emissions during composting of manure. *J Environ Manage* 2020; **271**: 110960.

123. Johnston AM, Bruulsema TW. 4R Nutrient Stewardship for Improved Nutrient Use Efficiency. *Procedia Engineering* 2014; **83**: 365-70.

124. Fixen PE. A brief account of the genesis of 4R nutrient stewardship. *Agron J* 2020; **112**: 4511-8.

125. Snyder CS. Enhanced nitrogen fertiliser technologies support the '4R' concept to optimise crop production and minimise environmental losses. *Soil Res* 2017; **55**: 463.

126. Pan B, Lam SK, Mosier A *et al*. Ammonia volatilization from synthetic fertilizers and its mitigation strategies: A global synthesis. *Agric Ecosyst Environ* 2016; **232**: 283-9.

127. Squire GR, Quesada N, Begg GS *et al*. Transitions to greater legume inclusion in

cropland: Defining opportunities and estimating benefits for the nitrogen economy. *Food Energy Secur* 2019; **8**: e00175.

128. Yigezu YA, El-Shater T, Boughlala M *et al.* Legume-based rotations have clear economic advantages over cereal monocropping in dry areas. *Agron Sustain Dev* 2019; **39**: 58.

129. Evenson RE, Gollin D. *Crop variety improvement and its effect on productivity: The impact of international agricultural research*: Cabi Publishing, 2003.

130. Lenaerts B, Collard BCY, Demont M. Review: Improving global food security through accelerated plant breeding. *Plant Sci* 2019; **287**: 110207.

131. Boughlala M, Gharras OE, Dahan R. Economic comparison between conventional and no-tillage farming systems in Morocco. 2013.

132. Derpsch R, Friedrich T, Kassam A *et al.* Current status of adoption of no-till farming in the world and some of its main benefits. *Int J Agric Biol Eng* 2010; **3**: 1-25.

133. Karayel D, šarauskis E. Environmental impact of no-tillage farming. *Environmental Research, Engineering and Management* 2019; **75**.

134. Bittman S, Dedina M, Howard CM *et al. Options for ammonia mitigation: Guidance from the UNECE task force on reactive nitrogen*: NERC/Centre for Ecology & Hydrology, 2014.

135. Li J, Li Y, Wan Y *et al.* Combination of modified nitrogen fertilizers and water saving irrigation can reduce greenhouse gas emissions and increase rice yield. *Geoderma* 2018; **315**: 1-10.

136. Vuolo F, Essl L, Atzberger C. Costs and benefits of satellite-based tools for irrigation management. *Front Environ Sci* 2015; **3**: 52.

137. Dey NC, Bala SK, Hayakawa S. Assessing the economic benefits of improved irrigation management: a case study in Bangladesh. *Water Policy* 2006; **8**: 573-84.

138. Sajeev EPM, Amon B, Ammon C *et al.* Evaluating the potential of dietary crude protein manipulation in reducing ammonia emissions from cattle and pig manure: A meta-analysis. *Nutr Cycl Agroecosyst* 2018; **110**: 161-75.

139. Liu S, Ni J, Radcliffe JS *et al.* Mitigation of ammonia emissions from pig production using reduced dietary crude protein with amino acid supplementation. *Bioresour Technol* 2017; **233**: 200-8.

140. Ndegwa PM, Hristov AN, Arogo J *et al.* A review of ammonia emission mitigation techniques for concentrated animal feeding operations. *Biosyst Eng* 2008; **100**: 453-69.

141. Oenema O, Oudendag DA, Witzke HP *et al.* Integrated measures in agriculture to reduce ammonia emissions: final summary report. Service contract" Integrated measures in agriculture to reduce ammonia emissions": Alterra, Department/Division; 2007. Report No.: 1566-7197.

142. Hayes ET, Leek ABG, Curran TP *et al.* The influence of diet crude protein level on odour and ammonia emissions from finishing pig houses. *Bioresour Technol* 2004; **91**: 309-15.

143. Witzke HP, Oenema O. Assessment of most promising measures. Task 3 Service

contract" Integrated measures in agriculture to reduce ammonia emissions": Alterra, Department/Division; 2007. Report No.: 1566-7197.

144. Pope T, Emmert JL. Phase-feeding supports maximum growth performance of broiler chicks from forty-three to seventy-one days of age. *Poultry Science* 2001; **80**: 345-52.

145. Woyengo TA, Beltranena E, Zijlstra RT. NONRUMINANT NUTRITION SYMPOSIUM: Controlling feed cost by including alternative ingredients into pig diets: A review. *J Anim Sci* 2014; **92**: 1293-305.

146. Loyon L, Burton CH, Misselbrook T *et al.* Best available technology for European livestock farms: Availability, effectiveness and uptake. *J Environ Manage* 2016; **166**: 1-11.

147. Wieczorek S. Assessing the influence of adsorbent bed (Tree bark) parameters on the reduction of ammonia emissions from animal husbandry. *Pol J Environ Stud* 2008; **17**: 147-54.

148. Smith MM, Simms CL, Aber JD. Case Study: Animal bedding cost and somatic cell count across New England dairy farms: Relationship with bedding material, housing type, herd size, and management system. *The Professional Animal Scientist* 2017; **33**: 616-26.

149. Le Riche EL, Vanderzaag A, Wagner-Riddle C *et al.* Do volatile solids from bedding materials increase greenhouse gas emissions for stored dairy manure? *Can J Soil Sci* 2017.

150. Reis S, Howard C, Sutton MA. *Costs of ammonia abatement and the climate co-benefits*: Springer, 2015.

151. Klimont Z, Winiwarter W. Estimating costs and potential for reduction of ammonia emissions from agriculture in the GAINS model. *Costs of ammonia abatement and the climate co-benefits* 2015: 233-61.

152. Swierstra D, Braam CR, Smits MC. Grooved floor system for cattle housing: Ammonia emission reduction and good slip resistance. *Appl Eng Agric* 2001; **17**: 85-90.

153. Boggia A, Paolotti L, Antegiovanni P *et al.* Managing ammonia emissions using no-litter flooring system for broilers: Environmental and economic analysis. *Environ Sci Policy* 2019; **101**: 331-40.

154. Predicala BZ, Cortus EL, Lemay SP *et al.* Effectiveness of a manure scraper system for reducing concentrations of hydrogen sulfide and ammonia in a swine grower-finisher room. *Trans ASABE* 2007; **50**: 999-1006.

155. Zhou S, Li Y, Liao X *et al.* A low-cost deodorizing spray net device for the removal of ammonia emissions in livestock houses. *J Clean Prod* 2021; **318**: 128516.

156. Melse RW, Ogink N. Air scrubbing techniques for ammonia and odor reduction at livestock operations: Review of on-farm research in the Netherlands. *Trans ASAE* 2005; **48**: 2303-13.

157. Costantini M, Bacenetti J, Coppola G *et al.* Improvement of human health and environmental costs in the European Union by air scrubbers in intensive pig farming. *J Clean Prod* 2020; **275**: 124007.

158. Melse RW, Mol G. Odour and ammonia removal from pig house exhaust air using a biotrickling filter. *Water Sci Technol* 2004; **50**: 275-82.

159. Hassouna M, Espagnol S, Robin P *et al*. Monitoring NH_3, N_2O, CO_2 and CH_4 emissions during pig solid manure storage—Effect of turning. *Compost Sci Util* 2008; **16**: 267-74.

160. Bicudo JR, Clanton CJ, Schmidt DR *et al*. Geotextile covers to reduce odor and gas emissions from swine manure storage ponds. *Appl Eng Agric* 2004; **20**: 65-75.

161. Wang S, Xu Z, Yang B *et al*. Low cost wastewater treatment in small towns and rural areas by simply-constructed alternate aerated ponds. *Journal of Tongji University. Natural Science* 2010; **38**: 1781-6.

162. Balsari P, Dinuccio E, Gioelli F. A low cost solution for ammonia emission abatement from slurry storage. *International Congress Series*. 2006. Pub Place: Elsevier; Year Published.

163. Zahn JA, Tung AE, Roberts BA *et al*. Abatement of ammonia and hydrogen sulfide emissions from a swine lagoon using a polymer biocover. *J Air Waste Manag Assoc* 2011; **51**: 562-73.

164. Wang Y, Guo H, Wang S *et al*. Sulfuric acid modified expanded vermiculite cover for reducing ammonia emissions from animal slurry storage. *J Hazard Mater* 2021; **403**: 123954.

165. Chen D, Sun J, Bai M *et al*. A new cost-effective method to mitigate ammonia loss from intensive cattle feedlots: application of lignite. *Sci Rep* 2015; **5**.

166. Shah SB, Grimes JL, Oviedo-Rondón EO *et al*. Acidifier application rate impacts on ammonia emissions from US roaster chicken houses. *Atmos Environ (1994)* 2014; **92**: 576-83.

167. Mao H, Zhang T, Li R *et al*. Apple pomace improves the quality of pig manure aerobic compost by reducing emissions of NH_3 and N_2O. *Sci Rep* 2017; **7**: 870.

168. Redding MR. Bentonite can decrease ammonia volatilisation losses from poultry litter: laboratory studies. *Anim Prod Sci* 2013; **53**: 1115-8.

169. Jiang T, Ma X, Yang J *et al*. Effect of different struvite crystallization methods on gaseous emission and the comprehensive comparison during the composting. *Bioresour Technol* 2016; **217**: 219-26.

170. Lei Y, Zhang Q, Nielsen C *et al*. An inventory of primary air pollutants and CO_2 emissions from cement production in China, 1990 – 2020. *Atmos Environ (1994)* 2011; **45**: 147-54.

171. Tang L, Ruan J, Bo X *et al*. Plant-level real-time monitoring data reveal substantial abatement potential of air pollution and CO_2 in China's cement sector. *One Earth* 2022; **5**: 892-906.

172. Qian H, Xu S, Cao J *et al*. Air pollution reduction and climate co-benefits in China's industries. *Nat Sustain* 2021; **4**: 417-25.

173. Ammar NR. Environmental and cost-effectiveness comparison of dual fuel propulsion options for emissions reduction onboard LNG carriers. *Brodogradnja: An International Journal of Naval Architecture and Ocean Engineering for Research and Development* 2019; **70**: 61-77.

174. Deng O, Huang S, Wang C *et al*. Atmospheric nitrogen pollution control benefits the coastal environment. *Environ Sci Technol* 2024; **58**: 449-58.

175. Alsebaeai MK, Ba-Shammakh MS, Ahmad AL *et al*. Optimal strategies of mitigation

options for NO_x emissions in the power generation industry. *Chem Eng Technol* 2023; **46**: 979-86.

176. Wang Y, Wang X, Ning M *et al*. The collaborative pollutants and carbon dioxide emission reduction and cost of ultra-low pollutant emission retrofit in China's cement kiln. *J Clean Prod* 2023; **405**.

177. Baukal CE, Hayes R, Grant M *et al*. Nitrogen oxides emissions reduction technologies in the petrochemical and refining industries. *Environ Prog* 2004; **23**: 19-28.

178. Cofala J, Syri S. *Nitrogen oxides emissions, abatement technologies and related costs for Europe in the RAINS model database*: JSTOR, 1998.

179. Andreoni V, Miola A, Perujo A. Cost effectiveness analysis of the emission abatement in the shipping sector emissions. *JRC Scientific and Technical Reports* 2008.

180. Deng J, Wang X, Wei Z *et al*. A review of NO_x and SO_x emission reduction technologies for marine diesel engines and the potential evaluation of liquefied natural gas fuelled vessels. *Sci Total Environ* 2021; **766**: 144319.

181. Frutos OD, Quijano G, Aizpuru A *et al*. A state-of-the-art review on nitrous oxide control from waste treatment and industrial sources. *Biotechnol Adv* 2018; **36**: 1025-37.

182. Pérez-Ramírez J. Ex-framework FeZSM-5 for control of N_2O in tail-gases. *Catal Today* 2002; **76**: 55-74.

183. Centi G, Vazzana F. Selective catalytic reduction of N_2O in industrial emissions containing O_2, H_2O and SO_2: behavior of Fe/ZSM-5 catalysts. *Catal Today* 1999; **53**: 683-93.

184. Yamada K, Kondo S, Segawa K. Selective catalytic reduction of nitrous oxide over Fe-ZSM-5: the effect of ion-exchange level. *Microporous Mesoporous Mat* 2000; **35-36**: 227-34.

185. van den Brink RW, Booneveld S, Pels JR *et al*. Catalytic removal of N_2O in model flue gases of a nitric acid plant using a promoted Fe zeolite. *Appl Catal B-Environ* 2001; **32**: 73-81.

186. Kameoka S, Kita K, Takeda T *et al*. Simultaneous removal of N_2O and CH_4 as the strong greenhouse-effect gases over Fe-BEA zeolite in the presence of excess O_2. *Catal Lett* 2000; **69**: 169-73.

187. Mauvezin M, Delahay G, Kisslich F *et al*. Catalytic reduction of N_2O by NH_3 in presence of oxygen using Fe-exchanged zeolites. *Catal Lett* 1999; **62**: 41-4.

188. Alves L, Holz LIV, Fernandes C *et al*. A comprehensive review of NO_x and N_2O mitigation from industrial streams. *Renew Sust Energ Rev* 2022; **155**: 111916.

189. Pérez-Ramı rez J, Kapteijn F, Schöffel K *et al*. Formation and control of N_2O in nitric acid production: where do we stand today? *Appl Catal B-Environ* 2003; **44**: 117-51.

190. Someshwar AV. Compilation of 'air toxic'and total hydrocarbon emissions data for sources at kraft, sulfite and non-chemical pulp mills. 2003.

191. Gao LZ, Au CT. Studies on the redox behaviour of La 1.867 Th 0.100 CuO_4 and its catalytic performance for NO decomposition. *Catal Lett* 2000; **65**: 91-8.

192. Zhu Y, Wang D, Yuan F *et al*. Direct NO decomposition over La_2-xBaxNiO_4 catalysts

containing $BaCO_3$ phase. *Appl Catal B-Environ* 2008; **82**: 255-63.

193. Yashnik SA, Anufrienko VF, Sazonov VA *et al*. Low-temperature activation of nitrogen oxide on Cu-ZSM-5 catalysts. *Kinet Catal* 2012; **53**: 363-73.

194. Li Y, Armor JN. Catalytic decomposition of nitrous oxide on metal exchanged zeolites. *Appl Catal B-Environ* 1992; **1**: L21-9.

195. Amrousse R, Tsutsumi A, Bachar A *et al*. N_2O catalytic decomposition over nano-sized particles of co-substituted Fe_3O_4 substrates. *Appl Catal A-Gen* 2013; **450**: 253-60.

196. Kumar S, Teraoka Y, Joshi AG *et al*. Ag promoted $La_{0.8}Ba_{0.2}MnO_3$ type perovskite catalyst for N_2O decomposition in the presence of O_2, NO and H_2O. *J Mol Catal A-Chem* 2011; **348**: 42-54.

197. Rauscher M. Preparation of a highly active Fe-ZSM-5 catalyst through solid-state ion exchange for the catalytic decomposition of N2O. *Appl Catal A-Gen* 1999; **184**: 249-56.

198. Zhang T, Qiu Y, Liu G *et al*. Nature of active Fe species and reaction mechanism over high-efficiency Fe/CHA catalysts in catalytic decomposition of N2O. *J Catal* 2020; **392**: 322-35.

199. Park J, Ahn J, Kim K *et al*. Historic and futuristic review of electron beam technology for the treatment of SO_2 and NO_x in flue gas. *Chem Eng J* 2019; **355**: 351-66.

200. Lee DH, Kim SD, Kim BN *et al*. Microwave effect in removal process of NO by electron beam irradiation and quantitative prediction of the removed NO. *Korean J Chem Eng* 2009; **26**: 1601-7.

201. Radoiu MT, Martin DI, Calinescu I. Emission control of SO_2 and NO_x by irradiation methods. *J Hazard Mater* 2003; **97**: 145-58.

202. Ighigeanu D, Martin D, Zissulescu E *et al*. SO_2 and NO_x removal by electron beam and electrical discharge induced non-thermal plasmas. *Vacuum* 2005; **77**: 493-500.

203. Kwon YK, Han DH. *M*icrowave Effect in the Simultaneous Removal of NO_x and SO_2 under Electron Beam Irradiation and Kinetic Investigation of NO_x Removal Rate. *Ind Eng Chem Res* 2010; **49**: 8147-56.

204. Ping L, Wang Y, Lee L *et al*. Cost-Benefit analysis of synergistic CO_2 and NO_x energy-efficient technologies for the road transport sector in China. *Atmosphere (Basel)* 2022; **13**: 1540.

205. Dėdelė A, Miškinytė A. Seasonal variation of indoor and outdoor air quality of nitrogen dioxide in homes with gas and electric stoves. *Environ Sci Pollut Res Int* 2016; **23**: 17784-92.

206. Nong G, Shen S, Chen T. Evaluation of ventilation effectiveness in the kitchens of Beijing flats. *Indoor Built Environ* 1996; **5**: 358-63.

207. Park Y, Lee K, Rhee Y. Removal characteristics of nitrogen oxide of high temperature catalytic filters for simultaneous removal of fine particulate and NOx. *J Ind Eng Chem* 2009; **15**: 36-9.

208. Mateus G, Meiller M, Soukup K *et al*. Dust filtration influence on the performance of catalytic filters for NO_x reduction. *Emission Control Sci Tech* 2018; **4**: 300-11.

209. Ozgen S, Cernuschi S, Caserini S. An overview of nitrogen oxides emissions from

biomass combustion for domestic heat production. *Renew Sust Energ Rev* 2021; **135**: 110113.

210. Wood A, Blackhurst M, Hawkins T *et al*. Cost-effectiveness of nitrogen mitigation by alternative household wastewater management technologies. *J Environ Manage* 2015; **150**: 344-54.

211. Zhao Y, Zhou Q, Hidetoshi K *et al*. Nitrogen flow characteristics of solid waste in China. *Ecotoxicol Environ Saf* 2021; **208**: 111596.

212. van Puijenbroek PJTM, Beusen AHW, Bouwman AF *et al*. Quantifying future sanitation scenarios and progress towards SDG targets in the shared socioeconomic pathways. *J Environ Manage* 2023; **346**: 118921.

213. Billen G, Aguilera E, Einarsson R *et al*. Reshaping the European agro-food system and closing its nitrogen cycle: The potential of combining dietary change, agroecology, and circularity. *One Earth* 2021; **4**: 839-50.

214. Bodirsky BL, Popp A, Lotze-Campen H *et al*. Reactive nitrogen requirements to feed the world in 2050 and potential to mitigate nitrogen pollution. *Nat Commun* 2014; **5**: 3858.

215. Qazi WA, Abushammala MF, Younes MK. Waste-to-energy technologies: a literature review. *The Journal of Solid Waste Technology and Management* 2018; **44**: 387-409.

216. Xiong J, Zheng Z, Yang X *et al*. Recovery of NH_3-N from mature leachate via negative pressure steam-stripping pretreatment and its benefits on MBR systems: A pilot scale study. *J Clean Prod* 2018; **203**: 918-25.

217. Ye Z, Hong Y, Pan S *et al*. Full-scale treatment of landfill leachate by using the mechanical vapor recompression combined with coagulation pretreatment. *Waste Manage* 2017; **66**: 88-96.

218. Shah TM, Ramaswami S, Behrendt J *et al*. Simultaneous removal of organics and ammonium-nitrogen from reverse osmosis concentrate of mature landfill leachate. *J Water Process Eng* 2017; **19**: 126-32.

219. Pradhan S, Fan L, Roddick FA. Removing organic and nitrogen content from a highly saline municipal wastewater reverse osmosis concentrate by UV/H_2O_2 – BAC treatment. *Chemosphere* 2015; **136**: 198-203.

220. Duan H, van den Akker B, Thwaites BJ *et al*. Mitigating nitrous oxide emissions at a full-scale wastewater treatment plant. *Water Res* 2020; **185**: 116196.

221. Yang Q, Liu X, Peng C *et al*. N_2O production during nitrogen removal via nitrite from domestic wastewater: main sources and control method. *Environ Sci Technol* 2009; **43**: 9400-6.

222. Karri RR, Sahu JN, Chimmiri V. Critical review of abatement of ammonia from wastewater. *J Mol Liq* 2018; **261**: 21-31.

223. Schmidt I, Sliekers O, Schmid M *et al*. New concepts of microbial treatment processes for the nitrogen removal in wastewater. *FEMS Microbiol Rev* 2003; **27**: 481-92.

224. Ashrafizadeh SN, Khorasani Z. Ammonia removal from aqueous solutions using hollow-fiber membrane contactors. *Chem Eng J* 2010; **162**: 242-9.

225. Wong RCK, Maini BB. Gas bubble growth in heavy oil-filled sand packs under

undrained unloading. *J Pet Sci Eng* 2007; **55**: 259-70.

226. Wang Z, Luo G, Li J *et al*. Response of performance and ammonia oxidizing bacteria community to high salinity stress in membrane bioreactor with elevated ammonia loading. *Bioresour Technol* 2016; **216**: 714-21.

227. Massara TM, Malamis S, Guisasola A *et al*. A review on nitrous oxide (N_2O) emissions during biological nutrient removal from municipal wastewater and sludge reject water. *Sci Total Environ* 2017; **596-597**: 106-23.

228. Zhu X, Chen Y, Chen H *et al*. Minimizing nitrous oxide in biological nutrient removal from municipal wastewater by controlling copper ion concentrations. *Appl Microbiol Biotechnol* 2013; **97**: 1325-34.

229. Chen Y, Wang D, Zhu X *et al*. Long-term effects of copper nanoparticles on wastewater biological nutrient removal and N_2O generation in the activated sludge process. *Environ Sci Technol* 2012; **46**: 12452-8.

230. Uludag-Demirer S, Demirer GN, Chen S. Ammonia removal from anaerobically digested dairy manure by struvite precipitation. *Process Biochem* 2005; **40**: 3667-74.

231. Tünay O, Kabdaşli I. Struvite precipitation from industrial and landfill waste streams. *Second International Conference on Recovery of Phosphates from sewage and animal wastes*. 2001. Pub Place; Year Published.

232. Zhang T, Ding L, Ren H *et al*. Ammonium nitrogen removal from coking wastewater by chemical precipitation recycle technology. *Water Res* 2009; **43**: 5209-15.

233. El Diwani G, El Rafie S, El Ibiari NN *et al*. Recovery of ammonia nitrogen from industrial wastewater treatment as struvite slow releasing fertilizer. *Desalination* 2007; **214**: 200-14.

234. Ryu H, Kim D, Lee S. Application of struvite precipitation in treating ammonium nitrogen from semiconductor wastewater. *J Hazard Mater* 2008; **156**: 163-9.

235. Yetilmezsoy K, Sapci-Zengin Z. Recovery of ammonium nitrogen from the effluent of UASB treating poultry manure wastewater by MAP precipitation as a slow release fertilizer. *J Hazard Mater* 2009; **166**: 260-9.

236. Gabelman A, Hwang S. Hollow fiber membrane contactors. *J Membr Sci* 1999; **159**: 61-106.

237. Turovskiy IS, Mathai PK. *Wastewater sludge processing*: John Wiley & Sons, 2006.

238. Quan X, Ye C, Xiong Y *et al*. Simultaneous removal of ammonia, P and COD from anaerobically digested piggery wastewater using an integrated process of chemical precipitation and air stripping. *J Hazard Mater* 2010; **178**: 326-32.

239. Pawlowski L, Lacy WJ, Uchrin CG *et al*. *Chemistry for the Protection of the Environment 2*: Springer Science & Business Media, 2012.

240. Huang H, Liu J, Zhang P *et al*. Investigation on the simultaneous removal of fluoride, ammonia nitrogen and phosphate from semiconductor wastewater using chemical precipitation. *Chem Eng J* 2017; **307**: 696-706.

241. Huang H, Liu J, Ding L. Recovery of phosphate and ammonia nitrogen from the

anaerobic digestion supernatant of activated sludge by chemical precipitation. *J Clean Prod* 2015; **102**: 437-46.

242. Jorgensen TC, Weatherley LR. Ammonia removal from wastewater by ion exchange in the presence of organic contaminants. *Water Res* 2003; **37**: 1723-8.

243. Kurama H. The application of membrane filtration for the removal of ammonium ions from potable water. *Water Res* 2002; **36**: 2905-9.

244. Wang Y, Liu S, Xu Z *et al*. Ammonia removal from leachate solution using natural Chinese clinoptilolite. *J Hazard Mater* 2006; **136**: 735-40.

245. Miladinovic N, Weatherley LR. Intensification of ammonia removal in a combined ion-exchange and nitrification column. *Chem Eng J* 2008; **135**: 15-24.

246. Edgerton BD, Mcnevin D, Wong CH *et al*. Strategies for dealing with piggery effluent in Australia: the sequencing batch reactor as a solution. *Water Sci Technol* 2000; **41**: 123-6.

247. Lahav O, Schwartz Y, Nativ P *et al*. Sustainable removal of ammonia from anaerobic-lagoon swine waste effluents using an electrochemically-regenerated ion exchange process. *Chem Eng J* 2013; **218**: 214-22.

248. Hussain S, Aziz H, Isa M *et al*. Physico-chemical method for ammonia removal from synthetic wastewater using limestone and GAC in batch and column studies. *Bioresour Technol* 2007; **98**: 874-80.

249. Okoniewska E, Lach J, Kacprzak M *et al*. The removal of manganese, iron and ammonium nitrogen on impregnated activated carbon. *Desalination* 2007; **206**: 251-8.

250. Halim AA, Aziz HA, Johari MAM *et al*. Comparison study of ammonia and COD adsorption on zeolite, activated carbon and composite materials in landfill leachate treatment. *Desalination* 2010; **262**: 31-5.

251. Vassileva P, Tzvetkova P, Nickolov R. Removal of ammonium ions from aqueous solutions with coal-based activated carbons modified by oxidation. *Fuel (Lond)* 2009; **88**: 387-90.

252. Marañón E, Ulmanu M, Fernández Y *et al*. Removal of ammonium from aqueous solutions with volcanic tuff. *J Hazard Mater* 2006; **137**: 1402-9.

253. Al-Haddad A, Chmielewska E, Al-Radwan S. A brief comparable lab examination for oil refinery wastewater treatment using the zeolitic and carbonaceous adsorbents. *Petroleum & Coal* 2007; **49**: 21-6.

254. Huang H, Xiao X, Yan B *et al*. Ammonium removal from aqueous solutions by using natural Chinese (Chende) zeolite as adsorbent. *J Hazard Mater* 2010; **175**: 247-52.

255. Le Leuch LM, Bandosz TJ. The role of water and surface acidity on the reactive adsorption of ammonia on modified activated carbons. *Carbon N Y* 2007; **45**: 568-78.

256. Liu H, Dong Y, Wang H *et al*. Ammonium adsorption from aqueous solutions by strawberry leaf powder: Equilibrium, kinetics and effects of coexisting ions. *Desalination* 2010; **263**: 70-5.

257. Pressley TA, Bishop DF, Roan SG. Ammonia-nitrogen removal by breakpoint chlorination. *Environ Sci Technol* 1972; **6**: 622-8.

258. Christianson LE, Cooke RA, Hay CH *et al*. Effectiveness of denitrifying bioreactors on water pollutant reduction from agricultural areas. *Trans ASABE* 2021; **64**: 641-58.

259. White SA, Morris SA, Wadnerkar PD *et al*. Anthropogenic nitrate attenuation versus nitrous oxide release from a woodchip bioreactor. *Environ Pollut* 2022; **300**: 118814.

260. Wadnerkar PD, White SA, Morris SA *et al*. Nitrate removal and nitrous oxide production from hothouse effluent draining to a pipe bioreactor. *Ecol Eng* 2022; **178**: 106561.

261. Hartfiel LM, Soupir ML, Rosentrater KA. Techno-economic analysis of constant-flow woodchip bioreactors. *Trans ASABE* 2021; **64**: 1545-54.

262. Schipper LA, Robertson WD, Gold AJ *et al*. Denitrifying bioreactors—An approach for reducing nitrate loads to receiving waters. *Ecol Eng* 2010; **36**: 1532-43.

263. Peng L, Carvajal-Arroyo JM, Seuntjens D *et al*. Smart operation of nitritation/denitritation virtually abolishes nitrous oxide emission during treatment of co-digested pig slurry centrate. *Water Res* 2017; **127**: 1-10.

264. Chen H, Zeng L, Wang D *et al*. Recent advances in nitrous oxide production and mitigation in wastewater treatment. *Water Res* 2020; **184**: 116168.

265. Shang HSYC, Peng YPBE, Zhang J *et al*. Effect of C/N Ratio on nitrous oxide production during denitrification with different electron acceptors. *Huanjing Kexue* 2009; **30**: 2007-12.

266. Frutos OD, Arvelo IA, Pérez R *et al*. Continuous nitrous oxide abatement in a novel denitrifying off-gas bioscrubber. *Appl Microbiol Biotechnol* 2015; **99**: 3695-706.

267. Frutos OD, Quijano G, Pérez R *et al*. Simultaneous biological nitrous oxide abatement and wastewater treatment in a denitrifying off-gas bioscrubber. *Chem Eng J* 2016; **288**: 28-37.

268. Joseph D, Edwards PE. Industrial wastewater engineering. *Seattle, WA* 1995; **98103**.

269. Park J, Jin H, Lim B *et al*. Ammonia removal from anaerobic digestion effluent of livestock waste using green alga Scenedesmus sp. *Bioresour Technol* 2010; **101**: 8649-57.

270. Koren DW, Gould WD, Bédard P. Biological removal of ammonia and nitrate from simulated mine and mill effluents. *Hydrometallurgy* 2000; **56**: 127-44.

271. Sabumon PC. Anaerobic ammonia removal in presence of organic matter: A novel route. *J Hazard Mater* 2007; **149**: 49-59.

272. Davidson J, Helwig N, Summerfelt ST. Fluidized sand biofilters used to remove ammonia, biochemical oxygen demand, total coliform bacteria, and suspended solids from an intensive aquaculture effluent. *Aquac Eng* 2008; **39**: 6-15.

273. Crab R, Kochva M, Verstraete W *et al*. Bio-flocs technology application in over-wintering of tilapia. *Aquac Eng* 2009; **40**: 105-12.

274. Aurelio Jr A, Lawson TB. Combination of a bead filter and rotating biological contactor in a recirculating fish culture system. *Aquac Eng* 1996; **15**: 27-39.

275. Avnimelech Y. Carbon/nitrogen ratio as a control element in aquaculture systems. *Aquaculture* 1999; **176**: 227-35.

276. Shnel N, Barak Y, Ezer T *et al*. Design and performance of a zero-discharge tilapia recirculating system. *Aquac Eng* 2002; **26**: 191-203.

277. Lin Y, Jing S, Lee D *et al*. Nutrient removal from aquaculture wastewater using a constructed wetlands system. *Aquaculture* 2002; **209**: 169-84.
278. Nora Aini A, Wahab Mohammad A, Jusoh A *et al*. Treatment of aquaculture wastewater using ultra-low pressure asymmetric polyethersulfone (PES) membrane. *Desalination* 2005; **185**: 317-26.
279. Brazil BL. Performance and operation of a rotating biological contactor in a tilapia recirculating aquaculture system. *Aquac Eng* 2006; **34**: 261-74.
280. Liu CCK, Xia W, Park JW. A wind-driven reverse osmosis system for aquaculture wastewater reuse and nutrient recovery. *Desalination* 2007; **202**: 24-30.
281. Sindilariu P, Schulz C, Reiter R. Treatment of flow-through trout aquaculture effluents in a constructed wetland. *Aquaculture* 2007; **270**: 92-104.
282. Xu X, Zhang X, Zhang S *et al*. Synergies of reducing greenhouse gases and atmospheric nitrogen pollutants in China.: Preprint at https://doi.org/10.21203/rs.3.rs-3282490/v1; 2023.
283. China Statistical Yearbook. China Statistical Publishing House: China Statistics Press; 2021.
284. Xu R, Tian H, Pan S *et al*. Global ammonia emissions from synthetic nitrogen fertilizer applications in agricultural systems: Empirical and process ‐ based estimates and uncertainty. *Glob Chang Biol* 2019; **25**: 314-26.
285. Huang X, Song Y, Li M *et al*. A high ‐ resolution ammonia emission inventory in China. *Global Biogeochem Cycles* 2012; **26**.
286. Luo Z, Lam SK, Fu H *et al*. Temporal and spatial evolution of nitrous oxide emissions in China: Assessment, strategy and recommendation. *J Clean Prod* 2019; **223**: 360-7.
287. Xie G, Han D, Wang X *et al*. Harvest index and residue factor of cereal crops in China. *Journal of China Agricultural University* 2011; **16**: 1-8.
288. *Foreign Agricultural Service*. https://fas.usda.gov/data (07 May 2024, date last accessed).
289. Gao Q, Zhang X, Liu L *et al*. A database of atmospheric inorganic nitrogen deposition fluxes in China from satellite monitoring. *Sci Data* 2023; **10**: 698.
290. Jia YL, Wang QF, Zhu JX *et al*. A spatial and temporal dataset of atmospheric inorganic nitrogen dry deposition in China (2006 ‐ 2015). *China Sci Data* 2021; **6**: 2021-4.
291. Jia Y, Wang Q, Zhu J *et al*. A spatial and temporal dataset of atmospheric inorganic nitrogen wet deposition in China (1996 ‐ 2015). *China Sci Data* 2019; **4**: 1-10.
292. Liu X, Zhang Y, Han W *et al*. Enhanced nitrogen deposition over China. *Nature* 2013; **494**: 459-62.
293. Walker JT, Bell MD, Schwede D *et al*. Aspects of uncertainty in total reactive nitrogen deposition estimates for North American critical load applications. *Sci Total Environ* 2019; **690**: 1005-18.
294. Peng L, Zhang Q, He K. Emissions inventory of atmospheric pollutants from open burning of crop residues in China based on a national questionnaire. *Research of Environmental Sciences* 2016; **29**: 1109-18.

295. FAOSTAT. FAO: FAO; 2023.

296. Zheng JY, Yin SS, Kang DW *et al.* Development and uncertainty analysis of a high-resolution NH_3 emissions inventory and its implications with precipitation over the Pearl River Delta region, China. *Atmos Chem Phys* 2012; **12**: 7041-58.

297. Zhang Y. Discussion on estimating nitrogen and phosphorus pollution loads in aquaculture. *Journal-Xiamen University Natural Science* 2003; **42**: 227-31.

298. Herbert RA. Nitrogen cycling in coastal marine ecosystems. *FEMS Microbiol Rev* 1999; **23**: 563-90.

299. Fu H, Luo Z, Hu S. A temporal-spatial analysis and future trends of ammonia emissions in China. *Sci Total Environ* 2020; **731**: 138897.

300. Luo Z, Hu S, Chen D. The trends of aquacultural nitrogen budget and its environmental implications in China. *Sci Rep* 2018; **8**.

301. He J, Wang L, Flynn DFB *et al.* Leaf nitrogen: phosphorus stoichiometry across Chinese grassland biomes. *Oecologia* 2008; **155**: 301-10.

302. Administration SF. National forest resources statistics: the eighth national forest resources inventory. China Forestry Publishing House, Beijing. Beijing: State Forestry Administration; 2014.

303. Cai ZC, Gu B, Ti CP *et al.* Guidelines for nitrogen flow analysis in China. *Science Press* 2018.

304. Wang F, Li J, Wang X *et al.* Nitrogen and phosphorus addition impact soil N_2O emission in a secondary tropical forest of South China. *Sci Rep* 2014; **4**: 5615.

305. Chen Y, Xu C, Ge Y *et al.* A 100-m gridded population dataset of China's seventh census using ensemble learning and geospatial big data. *Earth Syst Sci Data Discuss* 2024; **2024**: 1-19.

306. Song G, Li M, Semakula HM *et al.* Food consumption and waste and the embedded carbon, water and ecological footprints of households in China. *Sci Total Environ* 2015; **529**: 191-7.

307. Baker LA, Hope D, Xu Y *et al.* Nitrogen balance for the Central Arizona – Phoenix (CAP) ecosystem. *Ecosystems* 2001; **4**: 582-602.

308. Wei X, Wang X, Li L *et al.* Temporal and spatial characteristics of municipal solid waste generation and treatment in China from 1979 to 2016. *China Environ Sci* 2018; **38**: 3833-43.

309. Xu A, Wu Y, Chen Z *et al.* Towards the new era of wastewater treatment of China: Development history, current status, and future directions. *Water Cycle* 2020; **1**: 80-7.

310. Liu X, Beusen AH, van Puijenbroek PJ *et al.* Exploring wastewater nitrogen and phosphorus flows in urban and rural areas in China for the period 1970 to 2015. *Sci Total Environ* 2024; **907**: 168091.

311. Li J, Wang Y, Liu J *et al.* Intensified nitrogen removal by endogenous denitrification in a full-scale municipal wastewater treatment plant. *Environ Res* 2022; **205**: 112564.

312. Tallec G, Garnier J, Billen G *et al.* Nitrous oxide emissions from denitrifying activated sludge of urban wastewater treatment plants, under anoxia and low oxygenation. *Bioresour*

Technol 2008; **99**: 2200-9.

313. Gu B, Chang J, Min Y *et al*. The role of industrial nitrogen in the global nitrogen biogeochemical cycle. *Sci Rep* 2013; **3**: 2579.
